# Multivalent RNA‐Cleaving Agents on a Cubic Octameric Silsesquioxane Core

**DOI:** 10.1002/chem.71524

**Published:** 2026-08-08

**Authors:** Hanni Haapsaari, Iris Tuomi, Pasi Virta

**Affiliations:** ^1^ Department of Chemistry University of Turku Turku Finland

**Keywords:** artificial nucleases, azacrown, guanidine, RNA cleavage, silsesquioxanes

## Abstract

Selective RNA cleavage could provide versatile options in therapeutic applications. In this study, multivalent RNA‐cleaving agents, featuring 1,5,9‐triazacyclododecane or guanidine groups as catalytic centers, were synthesized on a cubic octameric silsesquioxane (COSS) core. Their catalytic efficiencies were first evaluated and compared in the presence and absence of metal ions Zn^2+^, Cu^2+^, and Pd^2+^ using hexaribonucleotide models. COSS–lysine(N_3_)–TACD–Zn^2+^_8_ exhibited base moiety selectivity for uracil bases, resulting in half‐lives of 2–11 h for the cleavage of the uracil‐containing hexaribonucleotides. Then, the cleavage of a biologically relevant HER2 mRNA model sequence was studied with the most effective catalysts. Surprisingly, an organocatalyst (COSS–lysine(N_3_)–arginine) outperformed a metal–organic catalyst (COSS–lysine(N_3_)–TACD–Zn^2+^_8_) in catalytic efficiency, the half‐lives being 14 and 31 h, respectively. In addition, the cleavage sites of the HER2 mRNA model were determined. While the COSS–lysine(N_3_)–TACD–Zn^2+^_8_ produced high background cleavage, the organic catalysts COSS–lysine(N_3_)–arginine and COSS–lysine(N_3_)–guanidine exhibited clear site‐specificity in the HER2 mRNA cleavage. Additionally, a correlation between the reaction rates and the amount of the catalysts was verified by varying the catalyst concentrations. Fast reaction rates and site‐specificity of the cleavage indicate good potential for further development of these cleaving agents.

## Graphical abstract

RNA‐cleaving agents on a cubic octameric silsesquioxane core were synthesized and characterized. Several catalytic systems of these structures were screened using hexaribonucleotide models. The best systems were then further used to study their catalytic efficiency in cleaving a HER2 mRNA model sequence. The induced cleavage sites were also determined. The cleavage activities were good, and site‐specificity of the cleavage was observed.

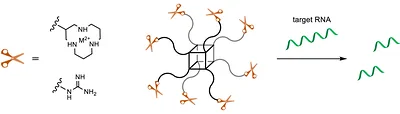

## 1 Introduction

Artificial ribonucleases have attracted interest as potential gene silencing therapeutics [1]. They are structures that consist of a phosphodiester cleaving agent and an oligonucleotide strand (the guide) that accelerates the phosphodiester cleavage by a hybridization‐driven sequence‐specific proximity effect between the cleaving agent and the RNA target [2]. In comparison to antisense oligonucleotides (ASOs) and small interfering RNAs (siRNAs), the mechanism of action of artificial ribonucleases is not dependent on intracellular enzymes. This is an advantage that can open possibilities for their structural modifications, as no substrate requirements for RNase H or RISC‐Ago2 machinery are needed. The hydrolytic cleavage of RNA occurs via transesterification [3, 4, 5]. The 2’‐OH group attacks the vicinal phosphate group, leading to a pentacoordinate phosphorane intermediate. Subsequent formation of a 2’,3’‐cyclic phosphate and the departure of the 5’‐*O*‐linked nucleoside causes phosphodiester bond cleavage, and the cyclic phosphate is rapidly hydrolyzed to a mixture of 2’‐ and 3’‐phosphates. The mechanism can be accelerated by assisting the proton transfer from the attacking 2’‐oxy‐nucleophile to the leaving 5’‐OH group. This can be achieved by (a) facilitating the deprotonation of 2’‐OH, (b) stabilizing the phosphorane intermediate, or (c) facilitating the protonation of the leaving 5’‐OH [6, 7].

These rules are utilized in the structural design of potential cleaving agents, which can be either metal‐ion complexes or purely organic catalysts based on Lewis acid/base interactions around the target phosphodiester bond [2]. Generally, organic catalysts are less effective than metal ion‐based catalysts [8], but they have one advantage: The activity of most metal ion‐based catalysts depends on the external metal ion concentration, which can pose a challenge in intracellular metal‐deficient environments [9]. On the other hand, the leaking of metal ions may cause unwanted degradation of non‐target nucleic acids or other biopolymers [10, 11, 12, 13]. Therefore, the independence of metal ions would make the use of organic cleaving agents advantageous.

Several natural enzymes catalyzing the cleavage of phosphodiester bonds contain one or more metal ions in their catalytic centers [14, 15, 16, 17]. Similarly, the most effective artificial catalysts contain complexes of metal ions such as lanthanides or 3d transition metals [2, 8, 18]. For instance, highly efficient PNAzymes utilize Zn(II)‐ and Cu(II)‐2,9‐dimethylphenanthroline for the cleavage [19, 20, 21, 22, 23]. Metal‐independent enzymes are also known. For example, the guanidine group is the active component in the catalytic centers of staphylococcal nuclease [24] and topoisomerase [25], as well as hammerhead [26, 27] and hairpin [28] ribozymes, whereas imidazole is the active component in the catalytic center of RNase A [29]. These functional groups have guided the design of organic cleaving agents. The most effective organic artificial nucleases comprise imidazole [30] or tris(2‐aminobenzimidazole) moieties [31, 32, 33, 34].

As recognized in natural ribonucleases and ribozymes, several catalytically active groups can lead to multiple simultaneous catalytic events or cooperation of the functions [17, 24, 25, 26, 27, 28, 29, 35, 36, 37]. Therefore, assembling multiple ligands around a suitable scaffold, or nanoparticle, may be beneficial for effective artificial ribonuclease activity. This has stimulated research on gold nanoparticles and metal‐oligonucleotide frameworks, called nanozymes, which aim for cooperative cleavage activity organized by multiple catalytic centers [38]. Besides components for RNA‐specific cleavage, the nanoparticles can also contain ligands (e.g., aptamers and glycans) for cell‐specific delivery [39, 40, 41, 42, 43]. However, most of such smart multifunctional nanoparticles are polydisperse. This complicates the in‐depth characterization and tuning of the structure‐activity relationships in these constructs. Cubic octameric silsesquioxanes (COSS, (RSiO_1.5_)_8_) [44, 45, 46, 47] with a fully condensed and highly symmetrical cage‐like structure could serve as particularly attractive scaffolds for artificial nucleases and their nanoparticle constructs. The inorganic silicate core can be combined with pendant functionalities, making it highly tailorable. Additionally, silsesquioxanes exhibit low toxicity and high biocompatibility, as their hydrolytic degradation leads to well‐known primary silsesquioxanes [48, 49].

We have previously shown that COSS is a suitable scaffold for the assembly of RNA‐cleaving agents, but the catalytic activity remained relatively low [50]. In this study, three different octavalent COSS cores as multivalent cleaving agents (**1**, **2**, and **3**) were synthesized (Scheme 1), and their catalytic RNA cleavage activity was examined. In addition to catalytic functionalities, the core units contained eight azide handles, enabling the potential future use of a robust click‐conjugation with biomolecules and/or a guide oligonucleotide strand, and hence, cell‐ and RNA‐specific targeting. One of the structures (**1**) contained 1,5,9‐triazacyclododecane as a metal‐coordinating catalytic center, whereas the other two (**2** and **3**) octavalent guanidium clusters represent organic metal‐independent cleaving agents. The efficiency of these cleaving agents was studied with four different hexaribonucleotide models, all of them in the presence and absence of metal ions (Zn^2+^, Cu^2+^, and Pd^2+^). With the hexaribonucleotide models, **1**‐(Zn^2+^)_8_ was the most efficient cleaving agent. It exhibited base moiety selectivity in cleavage and proved to be more efficient than previously reported di‐ and trivalent 1,5,9‐triazacyclododecane‐based cleaving agents. The cleavage activities of **1**‐(Zn^2+^)_8_ and the organic cleaving agents **2** and **3** were also studied with a biologically relevant HER2 (Human Epidermal Growth Factor Receptor 2) mRNA model sequence. Interestingly, site‐specific cleavage was observed with organocatalysts **2** and **3**. Furthermore, organocatalyst **3** was twice as efficient a cleaving agent as **1**‐(Zn^2+^)_8_.

**SCHEME 1:**
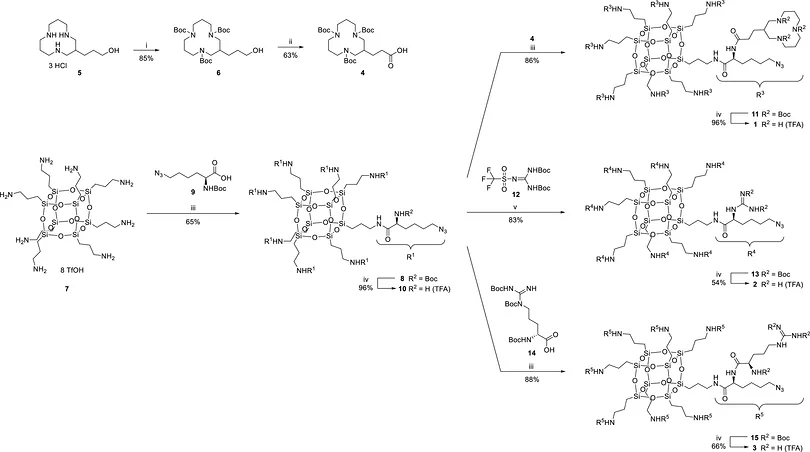
Synthesis of cleaving agents **1**, **2**, and **3**. Conditions: i) di‐*tert*‐butyl dicarbonate, Na_2_CO_3_, MeCN/H_2_O 1:1 (*v*/*v*), 24 h, r.t.; ii) CrO_3_, aq H_2_SO_4_, acetone, 1.5 h, 0°C, r.t.; iii) BOP, DIPEA, DMSO, 24 h, r.t.; iv) TFA/DCM 1:3 (*v*/*v*), 2 h, r.t.; v) Et_3_N, MeOH, 3 d, r.t.

## 2 Results and Discussion

### 2.1 Syntheses

3‐(1,5,9‐Tris(*tert*‐butyloxycarbonyl)‐1,5,9‐triazacyclododecan‐3‐yl) propanoic acid (**4**) was synthesized from 3‐(3‐hydroxypropyl)‐1,5,9‐triazacyclododecane HCl salt (**5**) in 54% yield by protecting the amino groups with Boc protecting groups and converting the primary alcohol (**6**) to the carboxylic acid by Jones oxidation. The aminopropyl‐functionalized COSS **7** was prepared by hydrolytic condensation of 3‐aminopropyltriethoxysilane (APTES) in acidic conditions. The octameric T8 cage was formed as the main product (24%), and the decameric T10 cage was formed as a by‐product (15%). COSS–lysine(N_3_) (**8**) was prepared in a 65% yield by amide coupling between **7** and an azido‐modified lysine (**9**) using BOP as a coupling reagent in the presence of DIPEA. The Boc protecting groups were removed with TFA in dichloromethane, affording the product **10** as a TFA salt. Amide coupling between **4** and **10**, followed by Boc removal of **11**, gave COSS–lysine(N_3_)–TACD (**1**) as a TFA salt. Nucleophilic substitution between **10** and Goodman's reagent (**12**), followed by Boc deprotection of **13**, gave COSS–lysine(N_3_)–guanidine (**2**) as a TFA salt. Amide coupling between **10** and Boc‐protected arginine **14**, and subsequent Boc removal, afforded COSS–lysine(N_3_)–arginine (**3**) as a TFA salt. Overall yields of **1**, **2**, and **3** from the aminopropyl‐functionalized COSS **7** were 52%, 28%, and 36%, respectively.

### 2.2 Cleavage of Hexaribonucleotide Models

Metal ions contribute to the cleavage of RNA by (a) decreasing the p*K*_a_ of the 2’‐OH group, thereby promoting the formation of the anionic nucleophile; (b) stabilizing the transition state by coordinating the non‐bridging oxygens; and (c) lowering the p*K*_a_ of the leaving group, thereby making it a better leaving group [51]. Multinuclear complexes are attractive due to the possible double Lewis acid activation, where both non‐bridging oxygens participate in the metal ion coordination [7, 52, 53]. Guanidines, in turn, benefit from cooperative catalysis of two guanidine groups, for example, as guanidine‐guanidium dyads [54, 55, 56, 57, 58, 59]. They may enhance the cleavage by (a) acting as general bases, thereby deprotonating the 2’‐OH group, (b) stabilizing the transition state through hydrogen bonding and positive charges, and (c) acting as general acids, thereby protonating the leaving 5’‐OH group [6, 60, 61].

The most effective divalent metal ions, Zn^2+^ and Cu^2+^, are attractive options as they are naturally present in intracellular environments [62, 63, 64, 65]. Their intracellular concentrations are considerable enough that ligands binding them can be expected to exist as complexes. Out of transition metal‐based catalysts, azacrowns are among the most efficient [2, 8, 18] and, therefore, were chosen for this study. Pd^2+^ is only a soft Lewis acid and not naturally present in intracellular environments, but it was used due to its potential to selectively bind nucleobases [66]. Guanidine was chosen to represent the organic catalytic center in **2** and **3**. The practical difference between **2** and **3** is the spacer length between the guanidine groups and the core, which likely affects the catalytic activity. These different structures also demonstrate the modularity of the COSS‐based cleaving agents, in which catalytic centers and their orientation can be varied by simple amide couplings.

Azacrown complexes of Zn^2+^ have been shown to recognize nucleobases [67, 68, 69, 70]. The strongest selectivity is for uracil and thymine and, to a lesser extent, for guanine. In terms of RNA cleavage, zinc complexes of azacrowns can simultaneously interact with both the phosphate and base moieties of the target RNA [71, 72, 73]. This accelerates the reaction as long as there are more azacrowns than uracil bases at the cleavage site. If all azacrown moieties are consumed in binding the uracil bases, none are left to induce cleavage. Laine et al. have previously [73] demonstrated that di‐ and tri‐nuclear 1,5,9‐triazacyclododecane‐based complexes (Zn^2+^)_2_ **L** ^1^ and (Zn^2+^)_3_ **L** ^3^ (Figure 1) are able to cleave short RNA strands site‐specifically when there are UX (X = A, C, and G) and UU (only in the case of (Zn^2+^)_3_ **L** ^3^) sections in the target sequence. However, the activity of the trinuclear cleaving agent was significantly reduced when the two uracil bases in the sequence were not adjacent but further apart.

**FIGURE 1:**
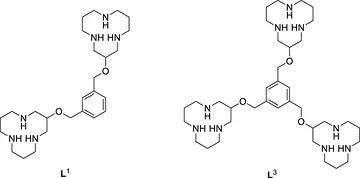
Azacrown structures studied by Laine et al. [73].

The intention was that increasing the number of azacrown moieties in **1** would improve the catalytic efficiency even further. For direct comparison, we used the same hexaribonucleotide models (shown in Figure 2) and the same reaction conditions as Laine et al. used in their study. Also, to allow direct comparison, the organic cleaving agents **2** and **3** were studied with the same hexaribonucleotide models in the same conditions, including in the presence of metal ions. The effective catalytic center concentration was matched to that of (Zn^2+^)_2_ **L** ^1^ of the previous study, that is, a lower cleaving agent concentration, to ensure that the results can be properly compared. The effective catalytic center concentration of (Zn^2+^)_3_ **L** ^3^ in the previous study was 1.5 × higher than that of (Zn^2+^)_2_ **L** ^1^ and our cleaving agents.

**FIGURE 2:**
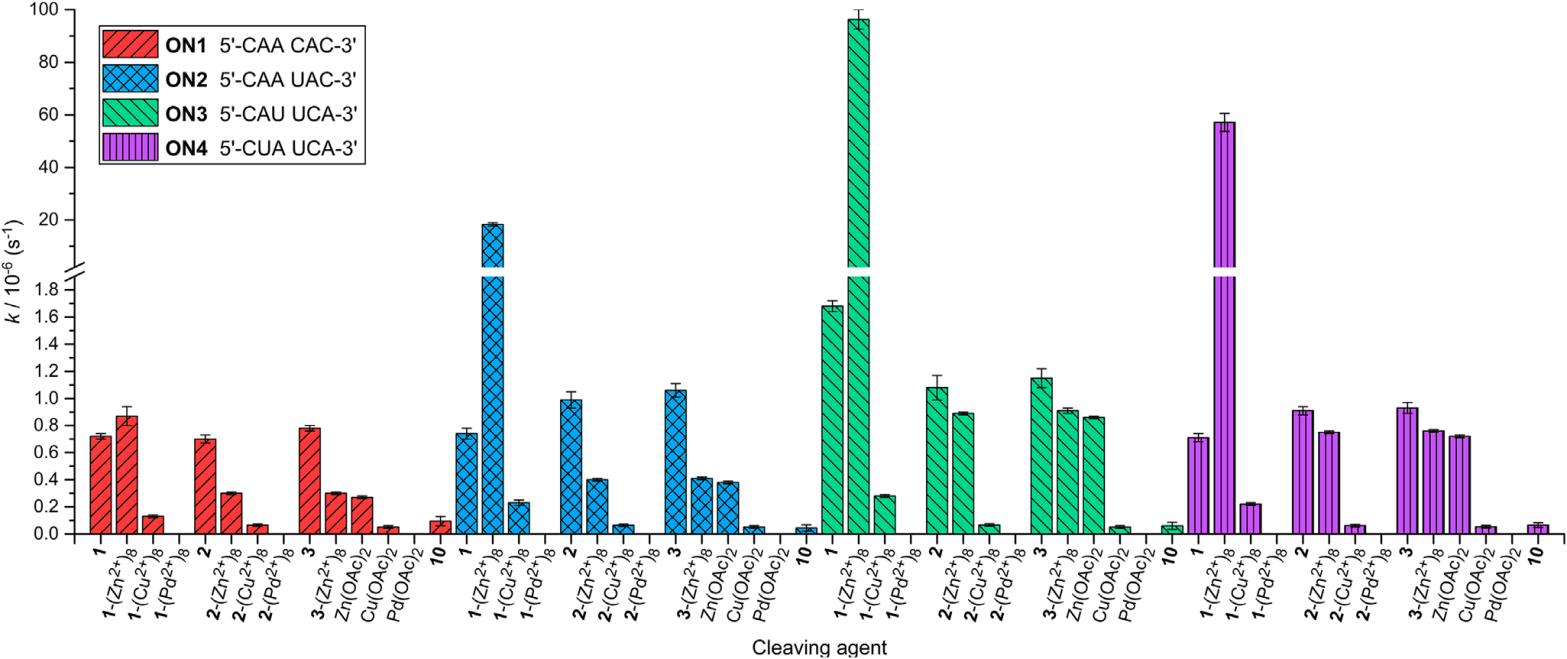
A comparison of the first‐order rate constants for the cleavage of the hexaribonucleotides (50 µM) by **1**, **2**, and **3** (25 µM) in the presence and absence of metal ions Zn^2+^, Cu^2+^, and Pd^2+^ (200 µM) in HEPES buffer (0.10 M, *I* = 0.10 M) at 35°C and pH 7.5 based on the data in Table S1.

For the reactions in the presence of metal ions, **1**, **2**, and **3** were pre‐mixed with 8 equivalents of the metal precursors. The degradation of model hexaribonucleotides was analyzed by RP‐HPLC to obtain *t*_1/2_ values of the decay. The cleavage was followed at 35°C and pH 7.5, and aliquots were withdrawn from the reaction solution at suitable time intervals. 4‐Nitrobenzenesulfonic acid was used as an internal standard (0.25 mM). All reactions were carried out in HEPES buffer (0.10 M, *I* = 0.10 M) using 25 µM cleaving agent **1**/**2**/**3** (*c*_eff_ = 200 µM) and 50 µM target hexaribonucleotide (**ON1**–**ON4**). The disappearance of the starting materials followed first‐order kinetics. For reference, we also followed the potential RNA cleavage with **10**. The reactions were also performed in the presence of metal ions (Zn(OAc)_2_, Cu(OAc)_2_, and Pd(OAc)_2_) only, and their amount (8 equivalents relative to **1**/**2**/**3**) was matched to the same reaction conditions. The reactions were followed for three half‐lives or up to 3 weeks (500 h). Two to four replicates of each reaction were performed to ensure the reliability of the results. Numerical values for the rate constants and the half‐lives of the cleavage are provided in the Supporting Information (Table S1).

Compound **1** worked best as a zinc complex. The copper complex (**1**‐(Cu^2+^)_8_) exhibited slow reaction rates, and the palladium complex (**1**‐(Pd^2+^)_8_) did not induce any observable cleavage within 3 weeks. The reaction rates induced by the metal complexes appear to be in the order of the Lewis acidity of the metal ions. The low activity of **1**‐(Cu^2+^)_8_ could also be attributed to the potential formation of intramolecular Cu─OH─Cu bridges. All the uracil‐containing sequences (**ON2–ON4**) cleaved relatively quickly (*t*_1/2_ = 10.5, 2.0, and 3.4 h, respectively) by **1**‐(Zn^2+^)_8_, and the structures are big enough that the activity was retained even in the case of two uracil bases further apart (**ON4**). All sequences, even the one without any uracil bases, were cleaved more efficiently by **1**‐(Zn^2+^)_8_ than observed with (Zn^2+^)**L** ^1^ or (Zn^2+^)**L** ^3^. Both **2** and **3** worked best without metal ions, as expected. In fact, the organocatalytic activity of **2** and **3** seems to be destroyed by the addition of metal ions, as the reaction rates are practically the same as with only the metal ions. A potential reason for this could be that the guanidine moieties might coordinate these metal ions at least to some extent, which then blocks the interactions required for the RNA cleavage. Although guanidinates, the anionic form of guanidines, have better coordination ability, metal coordination by neutral guanidines has also been reported [74, 75, 76]. There is no similar base moiety selectivity with **2** and **3**, but the cleavage rate of **ON1** is at a comparable level with **1**‐(Zn^2+^)_8_. Cleaving agent **3** was slightly more efficient than **2**. The rate constants from all reactions are visualized in Figure 2, showcasing the relative efficiencies of the cleaving agents.

### 2.3 Cleavage of HER2 mRNA Model

The cleavage activities of **1**‐(Zn^2+^)_8_, **2**, and **3** were next studied with a biologically relevant HER2 mRNA model sequence (**ON5**, 5’‐6FAM‐GUG AGC ACC AUG GAG‐3’). This sequence spans the HER2 mRNA region responsible for translation initiation, containing the start codon AUG [77, 78, 79]. Silencing of HER2 could potentially inhibit cancer cell growth [80]. **ON5** was labelled with fluorescein, and its degradation was analyzed by denaturing polyacrylamide gel electrophoresis (PAGE) to obtain *t*_1/2_ values of the decay. The reaction conditions were the same as above. A fluorescein‐labelled 2’‐OMe‐stabilized oligoribonucleotide (5’‐6FAM‐AAA AAA AAA AAA AAA AAA AAA‐3’) was used as an internal standard (25 µM). Three replicates of each reaction were performed to ensure the authenticity of the results. The disappearance of the starting material followed first‐order kinetics, as exemplified by Figure 3. In the absence of any cleaving agent in otherwise the same reaction conditions, **ON5** remains stable, and no cleavage can be observed in 3 weeks.

**FIGURE 3:**
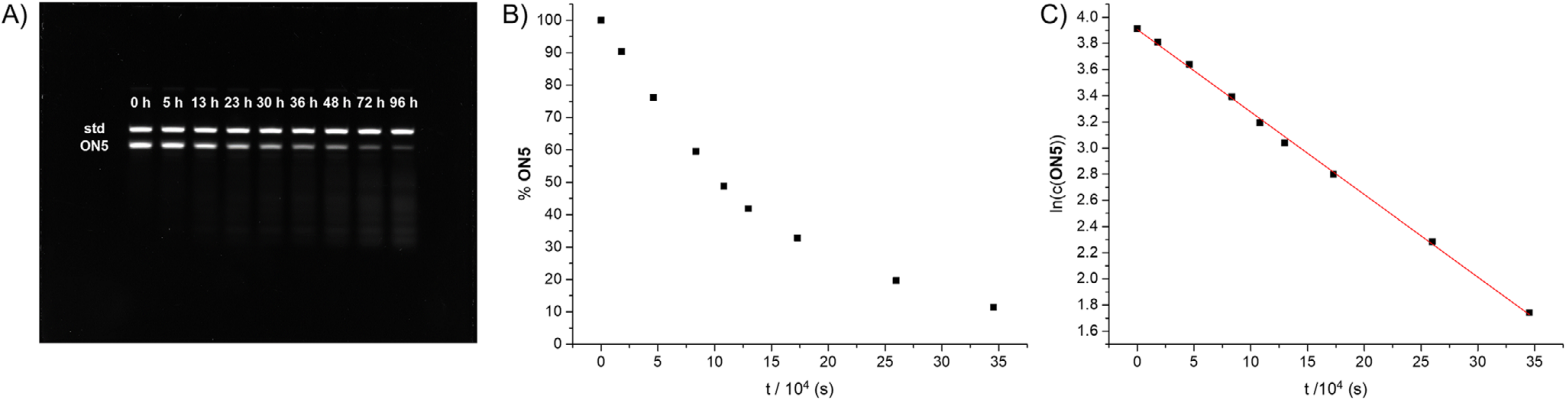
Cleavage of **ON5** (50 µM) by **1**‐(Zn^2+^)_8_ (25 µM) in HEPES buffer (0.10 M, *I* = 0.10 M) at 35°C and pH 7.5. (A) Example of a denaturing gel electrophoresis analysis of one of the replicates. (B) Decrease of the amount of intact target RNA **ON5** over time quantified from the gel bands of (A). (C) The reaction follows first‐order kinetics, demonstrated by plotting the natural logarithm of **ON5** concentration (µM) vs time (s).

Complex **1**‐(Zn^2+^)_8_ induced **ON5** decay with a half‐life of 30.6 ± 0.5 h (*k* = (6.3 ± 0.1) × 10^─6^ s^─1^). This is a slower decay than observed with the uracil‐containing hexaribonucleotides. The target HER2 mRNA sequence does not contain any UU or UXU (X = A, C, and G) sections, but GG and GXG (X = A, C, and U) sections, as well as GU and UG sections, as potential binding sites for **1**‐(Zn^2+^)_8_, are present in the target. However, the multiple potential binding sites could also lower the activity if these limit the availability of Zn^2+^‐azacrown complexes for bond cleavage. This might explain the lower activity of **1**‐(Zn^2+^)_8_ with the HER2 target. UPLC–MS analyses of the reaction mixtures (Figure 4) were used to determine the cleavage sites of the fragmentation (after approximately two half‐lives). With **1**‐(Zn^2+^)_8_, one cleavage site between U and G is a slightly more favored one (cf. fragments **13a** and **13b**, Table 1), but relatively high background cleavage (as opposed to site‐specific cleavage), resulting in multiple various‐length fragments, can be observed. This was expected, as the rate of the background cleavage is proportional to the number of phosphodiester bonds. Interestingly, the organic catalyst **3** exhibited a relatively fast cleavage of **ON5** with a half‐life of 14.2 ± 0.3 h (*k* = (13.5 ± 0.3) × 10^−6^ s^−1^), surpassing the efficiency of **1**‐(Zn^2+^)_8_.

**FIGURE 4:**
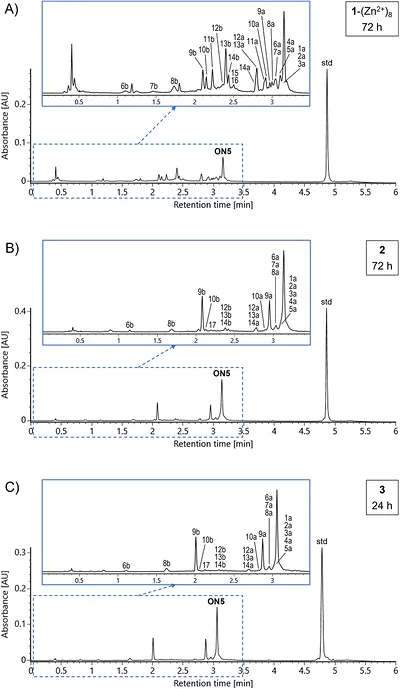
UV chromatograms and the corresponding **ON5** fragments identified by UPLC–MS analyses of the reaction mixtures with (A) **1**‐(Zn^2+^)_8_, (B) **2**, and (C) **3** after 72, 72, and 24 h, respectively.

**TABLE 1:** Cleavage sites of **ON5** induced by **1**‐(Zn^2+^)_8_, **2**, and **3** determined by UPLC–MS analyses of the reaction mixtures after 72, 72, and 24 h, respectively. The main cleavage sites induced by each catalyst are highlighted.

Cleaving agent	Fragment		Cleavage site
	**ON5**	5’‐6FAM GUG AGC ACC AUG GAG‐3’	
	**1a**	5’‐6FAM GUG AGC ACC AUG GA‐P‐3’	5’‐6FAM GUG AGC ACC AUG GA↓G‐3’
	**2a**	5’‐6FAM GUG AGC ACC AUG G‐P‐3’	5’‐6FAM GUG AGC ACC AUG G↓AG‐3’
	**3a**	5’‐6FAM GUG AGC ACC AUG‐P‐3’	5’‐6FAM GUG AGC ACC AUG ↓ GAG‐3’
	**4a**	5’‐6FAM GUG AGC ACC AU‐P‐3’	5’‐6FAM GUG AGC ACC AU↓G GAG‐3’
	**5a**	5’‐6FAM GUG AGC ACC A‐P‐3’	5’‐6FAM GUG AGC ACC A↓UG GAG‐3’
	**6a**	5’‐6FAM GUG AGC ACC‐P‐3’	5’‐6FAM GUG AGC ACC ↓ AUG GAG‐3’
	**6b**	5’‐AUG GAG‐3’	
	**7a**	5’‐6FAM GUG AGC AC‐P‐3’	5’‐6FAM GUG AGC AC↓C AUG GAG‐3’
	**7b**	5’‐C AUG GAG‐3’	
	**8a**	5’‐6FAM GUG AGC A‐P‐3’	5’‐6FAM GUG AGC A↓CC AUG GAG‐3’
	**8b**	5’‐CC AUG GAG‐3’	
**1**‐(Zn^2+^)_8_	**9a**	5’‐6FAM GUG AGC‐P‐3’	5’‐6FAM GUG AGC ↓ ACC AUG GAG‐3’
	**9b**	5’‐ACC AUG GAG‐3’	
	**10a**	5’‐6FAM GUG AG‐P‐3’	5’‐6FAM GUG AG↓C ACC AUG GAG‐3’
	**10b**	5’‐C ACC AUG GAG‐3’	
	**11a**	5’‐6FAM GUG A‐P‐3’	5’‐6FAM GUG A↓GC ACC AUG GAG‐3’
	**11b**	5’‐GC ACC AUG GAG‐3’	
	**12a**	5’‐6FAM GUG‐P‐3’	5’‐6FAM GUG ↓ AGC ACC AUG GAG‐3’
	**12b**	5’‐AGC ACC AUG GAG‐3’	
	**13a**	5’‐6FAM GU‐P‐3’	**5’‐6FAM GU↓G AGC ACC AUG GAG‐3’**
	**13b**	5’‐G AGC ACC AUG GAG‐3’	
	**14a**	5’‐6FAM G‐P‐3’	5’‐6FAM G↓UG AGC ACC AUG GAG‐3’
	**14b**	5’‐UG AGC ACC AUG GAG‐3’	
	**15**	5’‐G AGC ACC AUG GA‐P‐3’	5’‐6FAM GU↓G AGC ACC AUG GA↓G‐3’
	**16**	5’‐AGC ACC AUG GA‐P‐3’	5’‐6FAM GUG ↓ AGC ACC AUG GA↓G‐3’
**2** and **3**	**ON5**	5’‐6FAM GUG AGC ACC AUG GAG‐3’	
	**1a**	5’‐6FAM GUG AGC ACC AUG GA‐P‐3’	5’‐6FAM GUG AGC ACC AUG GA↓G‐3’
	**2a**	5’‐6FAM GUG AGC ACC AUG G‐P‐3’	5’‐6FAM GUG AGC ACC AUG G↓AG‐3’
	**3a**	5’‐6FAM GUG AGC ACC AUG‐P‐3’	5’‐6FAM GUG AGC ACC AUG ↓ GAG‐3’
	**4a**	5’‐6FAM GUG AGC ACC AU‐P‐3’	5’‐6FAM GUG AGC ACC AU↓G GAG‐3’
	**5a**	5’‐6FAM GUG AGC ACC A‐P‐3’	5’‐6FAM GUG AGC ACC A↓UG GAG‐3’
	**6a**	5’‐6FAM GUG AGC ACC‐P‐3’	5’‐6FAM GUG AGC ACC ↓ AUG GAG‐3’
	**6b**	5’‐AUG GAG‐3’	
	**7a**	5’‐6FAM GUG AGC AC‐P‐3’	5’‐6FAM GUG AGC AC↓C AUG GAG‐3’
	**8a**	5’‐6FAM GUG AGC A‐P‐3’	5’‐6FAM GUG AGC A↓CC AUG GAG‐3’
	**8b**	5’‐CC AUG GAG‐3’	
	**9a**	5’‐6FAM GUG AGC‐P‐3’	**5’‐6FAM GUG AGC ↓ ACC AUG GAG‐3’**
	**9b**	5’‐ACC AUG GAG‐3’	
	**10a**	5’‐6FAM GUG AG‐P‐3’	5’‐6FAM GUG AG↓C ACC AUG GAG‐3’
	**10b**	5’‐C ACC AUG GAG‐3’	
	**12a**	5’‐6FAM GUG‐P‐3’	5’‐6FAM GUG ↓ AGC ACC AUG GAG‐3’
	**12b**	5’‐AGC ACC AUG GAG‐3’	
	**13a**	5’‐6FAM GU‐P‐3’	5’‐6FAM GU↓G AGC ACC AUG GAG‐3’
	**13b**	5’‐G AGC ACC AUG GAG‐3’	
	**14a**	5’‐6FAM G‐P‐3’	5’‐6FAM G↓UG AGC ACC AUG GAG‐3’
	**14b**	5’‐UG AGC ACC AUG GAG‐3’	
	**17**	5’‐UG AGC ACC AU‐P‐3’	5’‐6FAM G↓UG AGC ACC AU↓G GAG‐3’

Moreover, a remarkable preference for site‐specific cleavage (cf. fragments **9a** and **9b**) can be observed, according to the UPLC–MS analysis of the reaction mixture. The same preferred cleavage site could also be observed with **2**, but the overall cleavage rate with a half‐life of 47.3 ± 1.2 h (*k* = (4.0 ± 0.1) × 10^−6^ s^−1^) remained at a similar level to that observed with **1**‐(Zn^2+^)_8_. However, it should be noted that the overall cleavage by **1**‐(Zn^2+^)_8_ consists of relatively high random background cleavage. Thus, the cleavage rates and the site‐specificity by both **2** and **3** were notable observations, showing potential for further development of the organic catalyst COSS cores. The reason for this site‐specificity is still unclear and requires further investigation. Cleavage and its site‐specificity of different RNA targets, including long biologically relevant RNA models, should be studied in detail. RNA transesterification requires alignment of the 2’‐OH nucleophile, the phosphorus, and the 5’‐OH leaving group [6]. Polyguanidine systems can bind to multiple phosphate oxygens simultaneously, which perhaps induces a specific RNA conformation that enables this in‐line geometry and, thus, exposes one particular phosphodiester bond for cleavage. The behavior might be similar to what can be found in biology, where many RNA‐binding proteins contain arginine‐rich motifs, such as arginine forks, that are responsible for RNA recognition [81, 82, 83, 84].

The cleavage of **ON5** (50 µM) was also explored with varying concentrations of the cleaving agents **1**‐(Zn^2+^)_8_ and the more efficient organic catalyst **3**. This was to confirm that the reaction rates are dependent on the concentration of the cleaving agents. Tested concentrations were 25, 18.75, 12.5, 6.25, and 3.125 µM (i.e., 4, 3, 2, 1, and 0.5 equivalents of catalytic centers relative to **ON5**, respectively). Upon lowering the concentrations of the catalysts, the cleavage of RNA is not expected to fully obey first‐order kinetics anymore. However, the correlation between ln(*c*(**ON5**)) and reaction time is assumed to be practically linear in a short enough time frame. On that basis, the rate constants for this purpose were also calculated from the integrated first‐order rate law. As can be seen from Figure 5, the reaction rates correlate with the concentration of the catalysts **1**‐(Zn^2+^)_8_ and **3**. **ON5** cleavage can be observed even at sub‐stoichiometric amounts of the catalysts.

**FIGURE 5:**
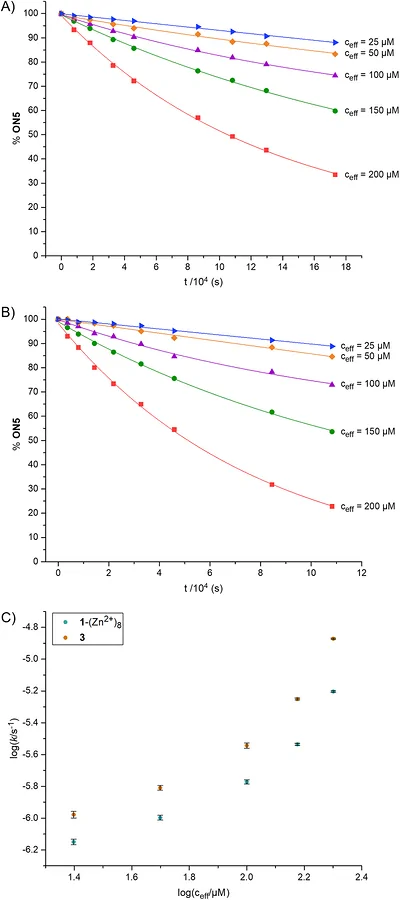
Cleavage of **ON5** with lower concentrations of (A) **1**‐(Zn^2+^)_8_ and (B) **3** in HEPES buffer (0.10 M, *I* = 0.10 M) at 35°C and pH 7.5. (C) Logarithmic rate constants for **ON5** cleavage as a function of logarithmic effective catalytic center concentration of **1**‐(Zn^2+^)_8_ and **3**.

## 3 Conclusion

Three different cleaving agents (**1**–**3**) were synthesized and characterized. Initially, cleavage of hexaribonucleotides was studied with all synthesized cleaving agents in the absence and presence of metal ions Zn^2+^, Cu^2+^, and Pd^2+^. This allowed direct comparison of the effect of the catalytic center and the effect of the metal ion on the cleavage. The structure with 1,5,9‐triazacyclododecane moieties as the catalytic centers (**1**) worked best as a zinc complex and exhibited base moiety selectivity to uracil nucleobases. The reaction rates even exceeded those of similar di‐ and trivalent compounds reported previously, even though the same effective catalytic center concentration was used (i.e., regarding the number of azacrown metal complexes). Even the sequence with non‐adjacent uracil bases was cleaved efficiently, which proves the intended superior and synergistic cleavage activity of **1**‐(Zn^2+^)_8_. The structures **2** and **3** with guanidine moieties worked best without any metal ions, compound **3** being a slightly more efficient cleaving agent. In contrast to **1**‐(Zn^2+^)_8_, **2** and **3** do not exhibit similar base moiety selectivity to uracil (or guanine) that could accelerate the cleavage via the proximity effect. Similar cleavage rates by **1**‐(Zn^2+^)_8_, **2**, and **3** can be observed with the hexaribonucleotide model, where uracil selectivity cannot be utilized.

The capability of **1**‐(Zn^2+^)_8_, **2**, and **3** to cleave a biologically relevant HER2 mRNA model sequence was studied. The cleavage activities of all three catalysts far exceeded the reaction rates of COSS–metallosalen–mannose described in our previous study [50]. **1**‐(Zn^2+^)_8_ cleaved **ON5** with a half‐life of 31 h, but with modest site‐specificity. Interestingly, high site‐specific cleavage of **ON5** could be observed with organocatalysts **2** and **3**. In addition, **3** turned out to be twice as efficient a cleaving agent as **1**‐(Zn^2+^)_8_. Overexpression of HER2 plays a vital role in the proliferation of breast cancer cells [85, 86], so **1**‐(Zn^2+^)_8_ and **3** have potential for further development and may be useful in cancer therapeutic applications in the future. Research on the guide strand conjugates of these COSS‐based cleaving agents is under way.

## 4 Experimental Section

### 4.1 General Methods and Materials

All reported reaction rate and half‐life values are from one replicate per reaction. Azido‐modified lysine (Boc‐Lys(N_3_)‐OH · CHA, **9** · CHA) was purchased from Biosynth, and Boc‐protected arginine (Boc‐Arg(Boc)_2_‐OH, **14**) was purchased from BLD Pharm. All plasticware and water used in the kinetic experiments were certified nuclease‐free. Oligonucleotides were synthesized by a conventional phosphoramidite strategy on a solid support using commercial phosphoramidite blocks. Deprotection and cleavage were performed with a 1:1 (*v*/*v*) mixture of 25% (*w*/*w*) aq ammonia and 40% (*w*/*w*) aq methylamine. 2’‐*O*‐TBDMS protecting groups of the RNAs were removed with triethylamine trishydrofluoride (Et_3_N · 3HF) and the oligonucleotides were precipitated from sodium acetate in 2‐propanol. The crude oligonucleotides were purified by RP‐HPLC using a Phenomenex Clarity Oligo‐RP (5 µm, 250 x 10 mm) semipreparative column. The yields of oligonucleotides were determined according to their UV absorbances at 260 nm. The mass spectra were recorded using a Waters Acquity UPLC‐DAD RDa MS electrospray ionization time‐of‐flight (ESI‐TOF) spectrometer, which was also used for the **ON5** cleavage site determination. The ^1^H, ^13^C, and ^29^Si NMR spectra were recorded on a Bruker Avance‐III 500 MHz instrument equipped with a Prodigy BBO CryoProbe. Characterizations were made with additional information from DEPT‐135, DQF‐COSY, multiplicity edited HSQC, and HMBC experiments. The reported ^1^H and ^13^C signals are referenced to the residual solvent signals [87], and the reported ^29^Si signals are referenced to TMS (*δ*_Si_ = 0.00 ppm) added to the samples.

### 4.2 Synthesis of 3‐(3‐Hydroxypropyl)‐1,5,9‐tris(*tert*‐butyloxycarbonyl)‐1,5,9‐triazacyclododecane (**6**)

3‐(3‐Hydroxypropyl)‐1,5,9‐triazacyclododecane HCl salt [88] **5** (0.60 g, 1 equivalent) and sodium carbonate (1.88 g, 10 equivalents) were dissolved in water (20 mL). Di*‐tert*‐butyl dicarbonate (1.28 g, 3.3 equivalents), separately dissolved in MeCN (20 mL), was added. The reaction mixture was stirred at room temperature overnight and then evaporated to dryness. Water (20 mL) and DCM (50 mL) were added, and the layers were separated. The aqueous layer was extracted with DCM (2 × 50 mL), and the combined organic layers were evaporated to dryness. The crude product was purified by silica gel column chromatography (EtOAc/Et_3_N 99:1 (*v*/*v*)), affording the product **6** as a white solid (0.80 g, 85%). ^1^H NMR (500 MHz, CDCl_3_, 25°C): *δ* = 3.61 (m, 6H), 3.30 (m, 4H), 2.96 (m, 4H), 1.99 (m, 3H), 1.85 (m, 2H), 1.60 (m, 2H), 1.45 (s, 27H), 1.41 ppm (m, 2H); ^13^C{^1^H} NMR (126 MHz, CDCl_3_, 25°C): *δ* = 156.5, 79.9, 62.9, 49.3, 47.9, 44.0, 36.6, 30.1, 29.1, 28.6, 26.9 ppm. HRMS (ESI) *m/z* calcd for C_27_H_52_N_3_O_7_^+^: 530.3800 [*M*+H]^+^, found 530.3791; *m/z* calcd for C_27_H_51_N_3_O_7_Na^+^: 552.3619 [*M*+Na]^+^, found 552.3639; *m/z* calcd for C_27_H_51_N_3_O_7_K^+^: 568.3359 [*M*+K]^+^, found 568.3431.

### 4.3 Synthesis of 3‐(1,5,9‐Tris(*tert*‐butyloxycarbonyl)‐1,5,9‐triazacyclododecan‐3‐yl) Propanoic Acid (**4**)

Starting material **6** (0.68 g, 1 equivalent) was dissolved in acetone (20 mL). Separately, concentrated sulfuric acid (0.64 mL) was added to chromium(VI) oxide (0.64 g), and the minimum possible amount of water (1.80 mL) was added until the CrO_3_ was just dissolved. In an ice‐water bath, small amounts of CrO_3_ solution (0.245 mL, 0.5 equivalents) were gradually added to the reaction mixture, and the reaction was followed by TLC (EtOAc) 5 min after every addition. CrO_3_ solution was added until the starting material had disappeared (in total 0.98 mL, 2 equivalents). After 30 min, the ice‐water bath was removed, and the reaction mixture was stirred at room temperature for an additional 1.5 h. The reaction mixture was neutralized with pyridine (10 mL), and the reaction was quenched with the addition of 2‐propanol (20 mL). The reaction mixture was evaporated to dryness. The evaporation residue was dissolved in DCM (30 mL), and the organic layer was washed with water (3 × 30 mL) and saturated NaCl (3 × 30 mL). The organic layer was evaporated to dryness. The crude product was purified by silica gel column chromatography (EtOAc), affording the product **4** as a white solid (0.44 g, 63%). ^1^H NMR (500 MHz, CDCl_3_, 25°C): *δ* = 3.58 (m, 4H), 3.30 (m, 4H), 2.96 (m, 4H), 2.40 (t, *J* = 7.5 Hz, 2H), 1.99 (m, 3H), 1.82 (m, 2H), 1.65 (m, 2H), 1.44 ppm (s, 27H); ^13^C{^1^H} NMR (126 MHz, CDCl_3_, 25°C): *δ* = 178.0, 156.5, 156.4, 80.2, 79.9, 49.3, 47.7, 44.1, 36.3, 31.1, 29.9, 28.6, 28.5, 26.3 ppm. HRMS (ESI) *m/z* calcd for C_27_H_48_N_3_O_8_^–^: 542.3447 [*M*–H]^–^, found 542.3441; *m/z* calcd for C_28_H_50_N_3_O_10_^–^: 588.3502 [*M*+HCOO]^–^, found 588.3448.

### 4.4 Synthesis of Aminopropyl‐COSS 8TfOH (**7**)

The synthesis and purification process were modified from procedures in the literature [89, 90]. Trifluoromethanesulfonic acid (2.6 mL, 1.5 equivalents) was added to water (58 mL), and the aqueous acidic solution was slowly added to (3‐aminopropyl)triethoxysilane (4.7 mL, 1 equivalent). The reaction mixture was stirred at room temperature overnight. After 12 h, the reaction mixture was transferred to a big, 800 mL beaker, and the open beaker was heated at 100°C until all solvent had evaporated. The beaker was cooled to room temperature, and acetone (60 mL) was added. The crude product was isolated by suction filtration and washed with acetone. A mixture of EtOH/MeOH 3:1 (*v*/*v*) (40 mL) was added to the dry precipitate, and the suspension was stirred at room temperature for 3 h. The mixture was transferred to a conical centrifuge tube and centrifuged. The supernatant was removed (but collected), and the precipitate was washed with acetone, MeCN, and DCM in suction filtration. The EtOH/MeOH and acetone/acetonitrile/DCM washings of the precipitate were repeated two more times. The precipitate was dried in vacuo, affording the octameric T8 product **7** as a white solid (1.26 g, 24%). The collected EtOH/MeOH supernatants were combined and evaporated to dryness. 2‐Propanol was added, and the suspension was stirred at room temperature for 1 h. The mixture was transferred to a conical centrifuge tube and centrifuged. The supernatant was removed, and the precipitate was dried in vacuo to afford the decameric T10 by‐product **16** as a white solid (0.80 g, 15%). The collected 2‐propanol supernatant was evaporated to dryness, but no dodecameric T12 by‐product had formed in the reaction. T8 product **7**: ^1^H NMR (500 MHz, DMSO‐*d6*, 25°C): *δ* = 7.57 (br s, 24 H), 2.77 (m, 16H), 1.57 (16H), 0.67 ppm (m, 16H); ^13^C{^1^H} NMR (126 MHz, DMSO‐*d6*, 25°C): *δ* = 120.6 (q, *J*_C,F_ = 322.8 Hz), 41.1, 20.6, 8.1 ppm; ^29^Si{^1^H} NMR (99 MHz, DMSO‐*d6*, 25°C): *δ* –66.55 ppm. HRMS (ESI) *m/z* calcd for C_24_H_65_N_8_O_12_Si_8_^+^: 881.2871 [*M*+H]^+^, found 881.2898; *m/z* calcd for C_24_H_66_N_8_O_12_Si_8_^2+^: 441.1472 [*M*+2H]^2+^, found 441.1434. T10 by‐product **16**: ^1^H NMR (500 MHz, DMSO‐*d6*, 25°C): *δ* = 7.56 (br s, 30H), 2.77 (m, 20H), 1.54 (m, 20H), 0.64 ppm (m, 20H); ^13^C{^1^H} NMR (126 MHz, DMSO‐*d6*, 25°C): *δ* = 120.6 (q, *J*_C,F_ = 321.6 Hz), 41.2, 20.7, 8.6 ppm; ^29^Si{^1^H} NMR (99 MHz, DMSO‐*d6*, 25°C): *δ* = –68.48 ppm. HRMS (ESI) *m/z* calcd for C_30_H_81_N_10_O_15_Si_10_^+^: 1101.3570 [*M*+H]^+^, found 1101.3624; *m/z* calcd for C_30_H_82_N_10_O_15_Si_10_^2+^: 551.1822 [*M*+2H]^2+^, found 551.1829; *m/z* calcd for C_30_H_83_N_10_O_15_Si_10_^3+^: 367.7905 [*M*+3H]^3+^, found 367.7883; *m/z* calcd for C_30_H_84_N_10_O_15_Si_10_^4+^: 276.0947 [*M*+4H]^4+^, found 276.0870.

### 4.5 Synthesis of COSS–lysine(N_3_)–Boc (**8**)

Dowex 50 W x 8 H^+^ (200–400 mesh) ion exchange resin was washed with 1 M aqueous HCl, water, and MeOH. The resin was rinsed with plenty of pyridine and dried in vacuo. Boc‐lysine(N_3_)‐OH cyclohexylammonium salt (0.35 g) was dissolved in MeOH (60 mL), and pyridinium Dowex was added. The mixture was stirred at room temperature for 4 h, and the resin was filtered out. Evaporation in vacuo afforded the Boc‐lysine(N_3_)‐OH (**9**) as a pale‐yellow oil (0.24 g, 93 %). Boc‐lysine(N_3_)‐OH (**9**, 0.2353 g, 12 equivalents), aminopropyl‐COSS (**7**, 0.1500 g, 1 equivalent), and benzotriazole‐1‐yl‐oxy‐tris‐(dimethylamino)‐phosphonium hexafluorophosphate (BOP) (0.4204 g, 1.1 equivalents relative to **9**) were dissolved in DMSO (10 mL), and *N*,*N*‐diisopropylethylamine (DIPEA) (0.497 mL, 3.3 equivalents relative to **9**) was added. The reaction mixture was stirred at room temperature overnight. After 24 h, the reaction mixture was lyophilized to dryness. The crude product was purified by silica gel column chromatography (DCM/MeOH 95:5 (*v*/*v*)). Subsequently, the crude product was dissolved in DCM and precipitated from MeCN. The precipitate was re‐purified by silica gel column chromatography (DCM/MeOH 97:3 (*v*/*v*)). Reprecipitation from MeCN and subsequent drying in vacuo afforded the product **8** as a white solid (0.1374 g, 65%). ^1^H NMR (500 MHz, CDCl_3_, 25°C): *δ* = 7.67 (br s, 8H), 5.92 (br s, 8H), 4.28 (m, 8H), 3.30 (m, 8H), 3.24 (t, *J* = 6.6 Hz, 16H), 3.06 (m, 8H), 1.68 (m, 16H), 1.61 (m, 32H), 1.47 (m, 16H), 1.41 (s, 72H), 0.65 ppm (m, 16H); ^13^C{^1^H} NMR (126 MHz, CDCl_3_, 25°C): *δ* = 173.0, 156.4, 79.8, 54.4, 51.4, 42.0, 32.9, 28.7, 28.5, 23.2, 23.0, 9.3 ppm; ^29^Si{^1^H} NMR (99 MHz, CDCl_3_, 25°C): *δ* = –66.66 ppm. HRMS (ESI) *m/z* calcd for C_112_H_209_N_40_O_36_Si_8_^+^: 2914.3902 [*M*+H]^+^, found 2914.3861; *m/z* calcd for C_112_H_210_N_40_O_36_Si_8_^2+^: 1457.6988 [*M*+2H]^2+^, found 1457.6965; *m/z* calcd for C_112_H_211_N_40_O_36_Si_8_^3+^: 972.1349 [*M*+3H]^3+^, found 972.1302.

### 4.6 Synthesis of COSS–lysine(N_3_) 8TFA (**10**)

Starting material **8** (0.1374 g) was dissolved in DCM (15 mL), and trifluoroacetic acid (TFA) (5 mL) was added. The reaction mixture was stirred at room temperature and followed by TLC (DCM/MeOH 90:10 (*v*/*v*)). After two hours, all the starting material had reacted. The volume of the reaction mixture was reduced by evaporation in vacuo. The remaining reaction mixture was added dropwise to cold diethyl ether. The suspension was centrifuged, and the supernatant was removed. The precipitate was dissolved in MeOH and evaporated in vacuo, affording the product **10** as a white solid (0.1369 g, 96%). ^1^H NMR (500 MHz, CD_3_OD, 25°C): *δ* = 3.88 (t, *J* = 6.5 Hz, 8H), 3.34 (t, *J* = 6.6 Hz, 16H), 3.29 (m, 8H), 3.19 (m, 8H), 1.88 (m, 16H), 1.64 (m, 32H), 1.49 (m, 16H), 0.68 ppm (m, 16H); ^13^C{^1^H} NMR (126 MHz, CD_3_OD, 25°C): *δ* = 170.1, 162.8 (q, *J*_C,F_ = 34.9 Hz), 118.1 (q, *J*_C,F_ = 292.7 Hz), 54.4, 52.1, 43.1, 32.2, 29.5, 23.7, 23.2, 10.1 ppm; ^29^Si{^1^H} NMR (99 MHz, CD_3_OD, 25°C): *δ* = –66.56 ppm. HRMS (ESI) *m/z* calcd for C_72_H_145_N_40_O_20_Si_8_^+^: 2113.9707 [*M*+H]^+^, found 2113.9764; *m/z* calcd for C_72_H_146_N_40_O_20_Si_8_^2+^: 1057.4890 [*M*+2H]^2+^, found 1057.4934; *m/z* calcd for C_72_H_147_N_40_O_20_Si_8_^3+^: 705.3284 [*M*+3H]^3+^, found 705.3219; *m/z* calcd for C_72_H_148_N_40_O_20_Si_8_^4+^: 529.2482 [*M*+4H]^4+^, found 529.2389.

### 4.7 Synthesis of COSS–lysine(N_3_)–TACD–3Boc (**11**)

Starting material **10** (0.0700 g, 1 equivalent), starting material **4** (0.1503 g, 12 equivalents), and BOP (0.1350 g, 1.1 equivalents relative to **4**) were dissolved in DMSO (7 mL), and DIPEA (0.160 mL, 3.3 equivalents relative to **4**) was added. The reaction mixture was stirred at room temperature overnight. Water (100 mL) was added, and the aqueous phase was extracted with diethyl ether (5 × 70 mL). The combined organic layer was washed with saturated aqueous NaCl (2 × 100 mL) and evaporated to dryness. The crude product was purified by silica gel column chromatography (EtOAc/MeOH/Et_3_N 89:10:1 (*v*/*v*/*v*)), affording the product **11** as a pale‐yellow solid (0.1269 g, 86%). ^1^H NMR (500 MHz, CDCl_3_, 25°C): *δ* = 7.51 (br s, 8H), 6.99 (br s, 8H), 4.50 (m, 8H), 3.58 (m, 32H), 3.28 (m, 32H), 3.24 (t, *J* = 6.6 Hz, 16H), 3.14 (m, 16H), 2.96 (m, 32H), 2.25 (m, 16H), 1.99 (m, 24H), 1.81 (m, 24H), 1.61 (m, 24H), 1.57 (m, 32H), 1.45 (s, 216H), 1.39 (m, 16H), 0.62 ppm (m, 16H); ^13^C{^1^H} NMR (126 MHz, CDCl_3_, 25°C): *δ* = 173.5, 172.1, 156.4, 156.3, 79.9, 79.8, 52.9, 51.2, 49.2, 47.7, 44.4, 42.0, 37.0, 33.1, 32.5, 29.8, 28.6, 27.4, 23.0, 22.9, 9.2 ppm; ^29^Si{^1^H} NMR (99 MHz, CDCl_3_, 25°C): *δ* = – 66.82 ppm. HRMS (ESI) *m/z* calcd for C_288_H_523_N_64_O_76_Si_8_^3+^: 2106.2388 [*M*+3H]^3+^, found 2106.2326; *m/z* calcd for C_288_H_524_N_64_O_76_Si_8_^4+^: 1579.9310 [*M*+4H]^4+^, found 1579.9043.

### 4.8 Synthesis of COSS–lysine(N_3_)–TACD 24TFA (**1**)

Starting material **11** (0.1258 g) was dissolved in DCM (3 mL), and TFA (1 mL) was added. The reaction mixture was stirred at room temperature for 2 h. The reaction mixture was added dropwise to cold diethyl ether. The suspension was centrifuged, and the supernatant was removed. The precipitate was dissolved in MeOH, and evaporation in vacuo afforded the product **1** as a pale‐yellow solid (0.1278 g, 96%). ^1^H NMR (500 MHz, CD_3_OD, 25°C): *δ* = 4.31 (m, 8H), 3.29 (m, 48H), 3.20 (m, 40H), 3.13 (m, 24H), 3.05 (m, 16H), 2.39 (t, *J* = 7.1 Hz, 16H), 2.09 (m, 40H), 1.79 (m, 8H), 1.69 (m, 24H), 1.61 (m, 32H), 1.46 (m, 16H), 0.65 ppm (m, 16H); ^13^C{^1^H} NMR (126 MHz, CD_3_OD, 25°C): *δ* = 175.0, 174.3, 162.1 (q, *J*_C,F_ = 36.3 Hz), 117.6 (q, *J*_C,F_ = 290.4 Hz), 55.0, 52.3, 51.2, 45.9, 45.0, 43.1, 35.0, 33.6, 32.9, 29.5, 27.5, 24.3, 23.7, 23.2, 10.1 ppm; ^29^Si{^1^H} NMR (99 MHz, CD_3_OD, 25°C): *δ* = –66.47 ppm. HRMS (ESI) *m/z* calcd for C_168_H_330_N_64_O_28_Si_8_^2+^: 1958.2255 [*M*+2H]^2+^, found 1958.2328; *m/z* calcd for C_168_H_331_N_64_O_28_Si_8_^3+^: 1305.8194 [*M*+3H]^3+^, found 1305.8217; *m/z* calcd for C_168_H_332_N_64_O_28_Si_8_^4+^: 979.6164 [*M*+4H]^4+^, found 979.5906; *m/z* calcd for C_168_H_333_N_64_O_28_Si_8_^5+^: 783.8946 [*M*+5H]^5+^, found 783.8787; *m/z* calcd for C_168_H_334_N_64_O_28_Si_8_^6+^: 653.4133 [*M*+6H]^6+^, found 653.3947.

### 4.9 Synthesis of COSS–lysine(N_3_)–guanidine–2Boc (**13**)

Starting material **10** (0.0781 g, 1 equivalent) and 1,3‐di‐Boc‐2‐(trifluoromethylsulfonyl)guanidine (**12**, 0.1616 g, 16 equivalents) were dissolved in MeOH (5 mL), and triethylamine (0.115 mL, 32 equivalents) was added. The reaction mixture was stirred at room temperature and followed by TLC (DCM/MeOH/TEA 94:5:1 and 89:10:1 (*v*/*v*/*v*)). After 3 days, the starting material had disappeared, and the reaction mixture was evaporated to dryness. The evaporation residue was dissolved in DCM (20 mL) and washed with water (20 mL), and the organic layer was evaporated to dryness. The crude product was purified by silica gel column chromatography (DCM/MeOH/Et_3_N 97:2:1 (*v*/*v*/*v*)). Dissolution in MeCN led to leftover impurity precipitating, which was then filtered out. Evaporation in vacuo afforded the product **13** as a white solid (0.0876 g, 83%). ^1^H NMR (500 MHz, CDCl_3_, 25°C): *δ* = 11.36 (s, 8H), 8.79 (d, *J* = 7.3 Hz, 8H), 7.22 (t, *J* = 5.4 Hz, 8H), 4.58 (m, 8H), 3.25 (m, 16H), 3.19 (m, 16H), 1.96 (m, 8H), 1.75 (m, 8H), 1.58 (m, 32H), 1.47 (s, 72H), 1.45 (s, 72H), 1.42 (m, 16H), 0.60 ppm (m, 16H); ^13^C{^1^H} NMR (126 MHz, CDCl_3_, 25°C): *δ* = 171.0, 163.3, 155.8, 152.9, 83.5, 79.3, 54.0, 51.3, 42.2, 31.6, 28.7, 28.4, 28.2, 22.8, 22.5, 9.3 ppm; ^29^Si{^1^H} NMR (99 MHz, CDCl_3_, 25°C): *δ* = –66.96 ppm. HRMS (ESI) *m/z* calcd for C_160_H_290_N_56_O_52_Si_8_^2+^: 2025.9957 [*M*+2H]^2+^, found 2026.0139; *m/z* calcd for C_160_H_291_N_56_O_52_Si_8_^3+^: 1350.9995 [*M*+3H]^3+^, found 1350.9853; *m/z* calcd for C_160_H_292_N_56_O_52_Si_8_^4+^: 1013.5015 [*M*+4H]^4+^, found 1013.5035; *m/z* calcd for C_160_H_293_N_56_O_52_Si_8_^5+^: 881.0026 [*M*+5H]^5+^, found 810.9999.

### 4.10 Synthesis of COSS–lysine(N_3_)–guanidine 16TFA (**2**)

Starting material **13** (0.0876 g) was dissolved in DCM (6 mL), and TFA (2 mL) was added. The reaction mixture was stirred at room temperature for 2 h. The reaction mixture was added dropwise to cold diethyl ether. The suspension was centrifuged, and the supernatant was removed. The precipitate was dissolved in MeOH, and evaporation in vacuo afforded the product **2** as a white solid (0.0502 g, 54%). The precipitation was not quantitative. ^1^H NMR (500 MHz, CD_3_OD, 25°C): *δ* = 4.18 (m, 8H), 3.32 (m, 16H), 3.29 (m, 8H), 3.15 (m, 8H), 1.91 (m, 8H), 1.79 (m, 8H), 1.62 (m, 32H), 1.46 (m, 16H), 0.65 ppm (m, 16H); ^13^C{^1^H} NMR (126 MHz, CD_3_OD, 25°C): *δ* = 172.1, 163.2 (q, *J*_C,F_ = 32.3 Hz), 158.5, 118.2 (q, *J*_C,F_ = 296.5 Hz), 56.3, 52.2, 43.2, 33.4, 29.5, 23.7, 23.6, 10.1 ppm; ^29^Si{^1^H} NMR (99 MHz, CD_3_OD, 25°C): *δ* = –66.48 ppm. HRMS (ESI) *m/z* calcd for C_80_H_162_N_56_O_20_Si_8_^2+^: 1225.5762 [*M*+2H]^2+^, found 1225.5765; *m/z* calcd for C_80_H_163_N_56_O_20_Si_8_^3+^: 817.3866 [*M*+3H]^3+^, found 817.3924.

### 4.11 Synthesis of COSS–lysine(N_3_)–arginine–3Boc (**15**)

Starting material **10** (0.0298 g, 1 equivalent), Boc‐Arg(Boc)_2_‐OH (**14**, 0.0560 g, 12 equivalents), and BOP (0.0574 g, 1.1 equivalents relative to **14**) were dissolved in DMSO (10 mL), and DIPEA (0.068 mL, 3.3 equivalents relative to **14**) was added. The reaction mixture was stirred overnight at room temperature. Water (20 mL) was added to the reaction mixture, and the aqueous layer was extracted with Et_2_O (10 mL). The organic layer was evaporated to dryness, and the crude product was purified by silica gel column chromatography (DCM/MeOH 98:2 (*v*/*v*) → DCM/MeOH 95:5 (*v*/*v*)), affording the product **15** as a white solid (0.0503 g, 88%). ^1^H NMR (500 MHz, CDCl_3_, 25°C): *δ* = 11.47 (s, 8H), 8.35 (t, *J* = 5.1 Hz, 8H), 7.49 (s, br, 8H), 7.28 (s, br, 8H), 5.87 (s, br, 8H), 4.54 (m, 8H), 4.12 (m, 8H), 3.39 (m, 16H), 3.24 (t, *J* = 6.8 Hz, 16H), 3.16 (m, 16H), 1.82 (m, 16H), 1.67 (m, 32H), 1.59 (m, 32H), 1.49 (s, 72H), 1.48 (s, 72H), 1.42 (s, 72H), 1.40 (m, 16H), 0.64 ppm (m, 16H); ^13^C{^1^H} NMR (126 MHz, CDCl_3_, 25°C): *δ* = 172.6, 171.6, 163.6, 156.4, 156.0, 153.3, 83.2, 80.1, 79.4, 55.0, 53.0, 51.3, 42.2, 40.5, 32.5, 29.1, 28.7, 28.49, 28.45, 28.2, 25.9, 22.8, 9.3 ppm; ^29^Si{^1^H} NMR (99 MHz, CDCl_3_, 25°C): *δ* = –66.85 ppm. HRMS (ESI) *m/z* calcd for C_240_H_436_N_72_O_76_Si_8_^4+^: 1441.7650 [*M*+4H]^4+^, found 1441.7784; *m/z* calcd for C_240_H_437_N_72_O_76_Si_8_^5+^: 1153.6134 [*M*+5H]^5+^, found 1153.6149; *m/z* calcd for C_240_H_438_N_72_O_76_Si_8_^6+^: 961.5124 [*M*+6H]^6+^, found 961.5031.

### 4.12 Synthesis of COSS–lysine(N_3_)–arginine 24TFA (**3**)

Starting material **15** (0.0503 g) was dissolved in DCM (6 mL), and TFA (2 mL) was added. The reaction mixture was stirred at room temperature for 2 h. The reaction mixture was added dropwise to cold diethyl ether. The suspension was centrifuged, and the supernatant was removed. The precipitate was dissolved in MeOH, and evaporation in vacuo afforded the product **3** as a white solid (0.0351 g, 66%). The precipitation was not quantitative. ^1^H NMR (500 MHz, CD_3_OD, 25°C): *δ* = 4.37 (m, 8H), 4.02 (t, *J* = 6.3 Hz, 8H), 3.31 (m, 24H), 3.23 (t, *J* = 6.8 Hz, 16H), 3.09 (m, 8H), 1.95 (m, 16H), 1.82 (m, 8H), 1.74 (m, 24H), 1.63 (m, 32H), 1.48 (m, 16H), 0.67 ppm (m, 16H); ^13^C{^1^H} NMR (126 MHz, CD_3_OD, 25°C): *δ* = 173.8, 170.0, 163.1 (q, *J*_C,F_ = 34.7 Hz), 158.8, 118.1 (q, *J*_C,F_ = 292.6 Hz), 55.1, 53.8, 52.3, 43.2, 41.8, 33.0, 29.7, 29.6, 25.2, 24.2, 23.7, 10.2 ppm; ^29^Si{^1^H} NMR (99 MHz, CD_3_OD, 25°C): *δ* = –66.54 ppm. HRMS (ESI) *m/z* calcd for C_120_H_242_N_72_O_28_Si_8_^2+^: 1681.8935 [*M*+2H]^2+^, found 1681.8850; *m/z* calcd for C_120_H_243_N_72_O_28_Si_8_^3+^: 1121.5981 [*M*+3H]^3+^, found 1121.6057; *m/z* calcd for C_120_H_244_N_72_O_28_Si_8_^4+^: 841.4504 [*M*+4H]^4+^, found 841.4512; *m/z* calcd for C_120_H_245_N_72_O_28_Si_8_^5+^: 673.3617 [*M*+5H]^5+^, found 673.3648.

### 4.13 Kinetic Experiments of Hexaribonucleotides

Cleaving agents **1**/**2**/**3** (1 equivalent) were dissolved in water, and metal precursors Zn(OAc)_2_ · 2H_2_O, Cu(OAc)_2_ · H_2_O, or Pd(OAc)_2_ (8 equivalents) dissolved in water were added. The solutions were mixed overnight, and water was added until the cleaving agent concentration was 100 µM. The reactions were carried out in microcentrifuge tubes and heated at 35°C with a dry heat block. 50 µL HEPES buffer (0.40 M in H_2_O, pH 7.5, ionic strength adjusted to 0.40 M with NaCl), 50 µL 4‐nitrobenzenesulfonic acid (1 mM in H_2_O), 50 µL target oligonucleotide (200 µM in H_2_O), and 50 µL cleaving agent (100 µM in H_2_O) were added to a tube. Consequently, the total reaction volume was 200 µL (*c*(HEPES) = 0.10 M), *I*(HEPES) = 0.1 M, *c*(std) = 0.25 mM, *c*(target‐ON) = 50 µM, *c*(cat.) = 25 µM) with a pH of 7.5. In the reference reactions, 50 µL Zn(OAc)_2_ · 2H_2_O (800 µM), 50 µL Cu(OAc)_2_ (800 µM), or 50 µL Pd(OAc)_2_ (800 µM) was added instead of the cleaving agent to match the reaction conditions. 20 µL aliquots were withdrawn at suitable time intervals and cooled immediately on an ice‐water bath. The reaction in the samples was quenched by the addition of 3 µL 1 M aq HCl. 80 µL of water was added to the samples, and they were analyzed by RP‐HPLC as soon as possible. The column used was an analytical Thermo Scientific ODS Hypersil column (dim. (mm) 250 × 4.6, particle size (µm) 5), and 50 mM triethylammonium acetate buffers in H_2_O and MeCN were used. The integral values obtained for the target hexaribonucleotides were corrected by dividing them by the integral values obtained for the standard and then normalized to the starting point. First‐order rate constants were calculated for the disappearance of the target oligonucleotide by applying the integrated first‐order rate law to the diminution of the signal from the intact hexaribonucleotide.

### 4.14 Kinetic Experiments of **ON5**

The experiments were executed similarly to those previously reported [50]. The reactions were carried out in microcentrifuge tubes and heated at 35°C with a dry heat block. 12 µL HEPES buffer (0.40 M in H_2_O, pH 7.5, ionic strength adjusted to 0.40 M with NaCl), 6 µL internal standard oligonucleotide (200 µM in H_2_O), 6 µL H_2_O, 12 µL target oligonucleotide (200 µM in H_2_O), and 12 µL cleaving agent (100 µM in H_2_O) were added to a tube. Consequently, the total reaction volume was 48 µL (*c*(HEPES) = 0.10 M), *I*(HEPES) = 0.1 M, *c*(std‐ON) = 25 µM, *c*(target‐ON) = 50 µM, *c*(cat.) = 25 µM) with a pH of 7.5. In the experiments with lower cleaving agent concentrations, 9, 6, 3, or 1.5 µL of cleaving agent was added, and the remaining volume was replaced with water. 3 µL aliquots were withdrawn at suitable time intervals and cooled immediately on an ice‐water bath. The aliquots were diluted 10‐fold with H_2_O, and a 3 µL sample from the dilution was mixed with 3 µL Novex TBE‐Urea 2× sample buffer. The samples were frozen with liquid N_2_ and kept in a freezer until denaturing polyacrylamide gel electrophoresis (PAGE) was performed to analyze the cleavage of **ON5**. 4 µL of the samples were applied to gel wells. The gel used was a TBE‐Urea gel (15% acrylamide, 1 × TBE, 7 M urea) fixed into a vertical electrophoresis chamber, which was filled with the running buffer (90 mM Tris, 90 mM borate, 2 mM EDTA, pH 8.3). The gels were run at 200 V and 45 mA for 45 min, and they were imaged by the fluorescence labels in the standard oligonucleotide and the target oligonucleotide. The bands on the gels were quantified by processing the images into 2D density profiles and integrating them using ImageJ software. The values obtained for **ON5** were corrected by dividing them by the values obtained for the standard and then normalized to the starting point. First‐order rate constants were calculated for the disappearance of the target oligonucleotide by applying the integrated first‐order rate law to the diminution of the signal from **ON5**.

## Conflicts of Interest

The authors declare no conflicts of interest.

## Supporting information




**Supporting File 1**: chem71524‐sup‐0001‐SuppMat.pdf

## Data Availability

The data that support the findings of this study are available from the corresponding author upon reasonable request.
