# Supplementary material for: Multivalent RNA‐Cleaving Agents on a Cubic Octameric Silsesquioxane Core
Source: Chemistry. 2026 Aug 8;32(41):e71524. doi: 10.1002/chem.71524 (PMC13729976; doi:10.1002/chem.71524)
Supplement: Supplementary file 1 [file CHEM-32-e71524-s001.pdf]

Supporting Information (SI)

Multivalent RNA-Cleaving Agents on a Cubic Octameric Silsesquioxane Core

Hanni Haapsaari,[a] Iris Tuomi,[a] and Pasi Virta*[a]

[a] H. Haapsaari, I. Tuomi, Prof. P. Virta
Department of Chemistry
University of Turku
Henrikinkatu 2, 20500 Turku, Finland
E-mail: pamavi@utu.fi

Table of contents

1 Kinetic Data of Hexaribonucleotide Cleavage S2
2 NMR Spectra S3
3 Mass Spectra S20

1 Kinetic Data of Hexaribonucleotide Cleavage

Table S1. First-order rate constants and half-lives for the cleavage of hexaribonucleotides (50 µM) by **1**, **2**, and **3** (25 µM) in the presence and absence of metal ions Zn^{2+}, Cu^{2+}, and Pd^{2+} (200 µM) in HEPES buffer (0.10 M, *I* = 0.10 M) at 35 °C and pH 7.5.

Cleaving agent	k / 10^{-6} (s^{-1})<br>$t_{1/2}$ (h)			
	**ON1** 5’-CAA CAC-3’	**ON2** 5’-CAA UAC-3’	**ON3** 5’-CAU UCA-3’	**ON4** 5’-CUA UCA-3’
**1**	0.72 ± 0.02<br>265 ± 7	0.74 ± 0.04<br>259 ± 14	1.68 ± 0.04<br>115 ± 3	0.71 ± 0.03<br>271 ± 9
**1**-$(Zn^{2+})_8$	0.87 ± 0.07<br>221 ± 17	18.3 ± 0.6<br>10.5 ± 0.3	96.3 ± 3.7<br>2.0 ± 0.2	57.1 ± 3.4<br>3.4 ± 0.2
**1**-$(Cu^{2+})_8$	0.13 ± 0.01<br>1475 ± 80	0.23 ± 0.02<br>834 ± 42	0.28 ± 0.01<br>685 ± 24	0.22 ± 0.01<br>841 ± 20
**1**-$(Pd^{2+})_8$	stable	stable	stable	stable
**2**	0.70 ± 0.03<br>274 ± 11	0.99 ± 0.06<br>194 ± 11	1.08 ± 0.09<br>179 ± 15	0.91 ± 0.03<br>212 ± 5
**2**-$(Zn^{2+})_8$	0.30 ± 0.01<br>642 ± 10	0.40 ± 0.01<br>473 ± 10	0.89 ± 0.01<br>216 ± 2	0.75 ± 0.01<br>256 ± 3
**2**-$(Cu^{2+})_8$	~ 3000 h	~ 3000 h	~ 3000 h	~ 3000 h
**2**-$(Pd^{2+})_8$	stable	stable	stable	stable
**3**	0.78 ± 0.02<br>247 ± 5	1.06 ± 0.05<br>180 ± 8	1.15 ± 0.07<br>167 ± 9	0.93 ± 0.04<br>206 ± 8
**3**-$(Zn^{2+})_8$	0.30 ± 0.01<br>636 ± 14	0.41 ± 0.01<br>471 ± 10	0.91 ± 0.02<br>211 ± 3	0.76 ± 0.01<br>251 ± 3
$Zn(OAc)_2$	0.27 ± 0.01<br>689 ± 7	0.38 ± 0.01<br>497 ± 8	0.86 ± 0.01<br>224 ± 3	0.72 ± 0.01<br>267 ± 3
$Cu(OAc)_2$	~ 4000 h	~ 4000 h	~ 4000 h	~ 4000 h
$Pd(OAc)_2$	stable	stable	stable	stable
**10**	~ 3000 h	~ 3000 h	~ 3000 h	~ 3000 h

2 NMR Spectra

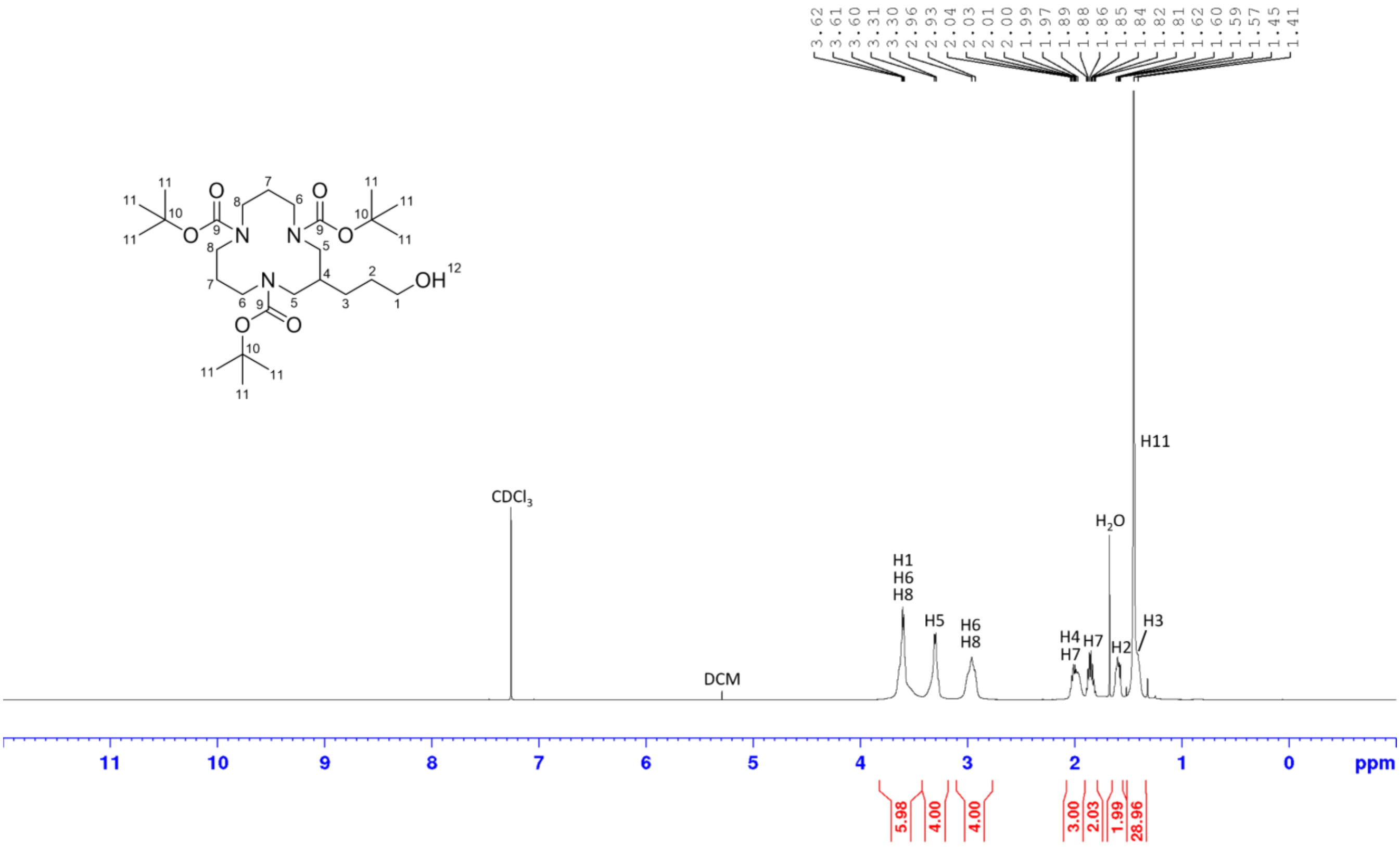

Figure S1. ^{1}H NMR spectrum of **6** ($CDCl_3$, 500 MHz, 25 °C).

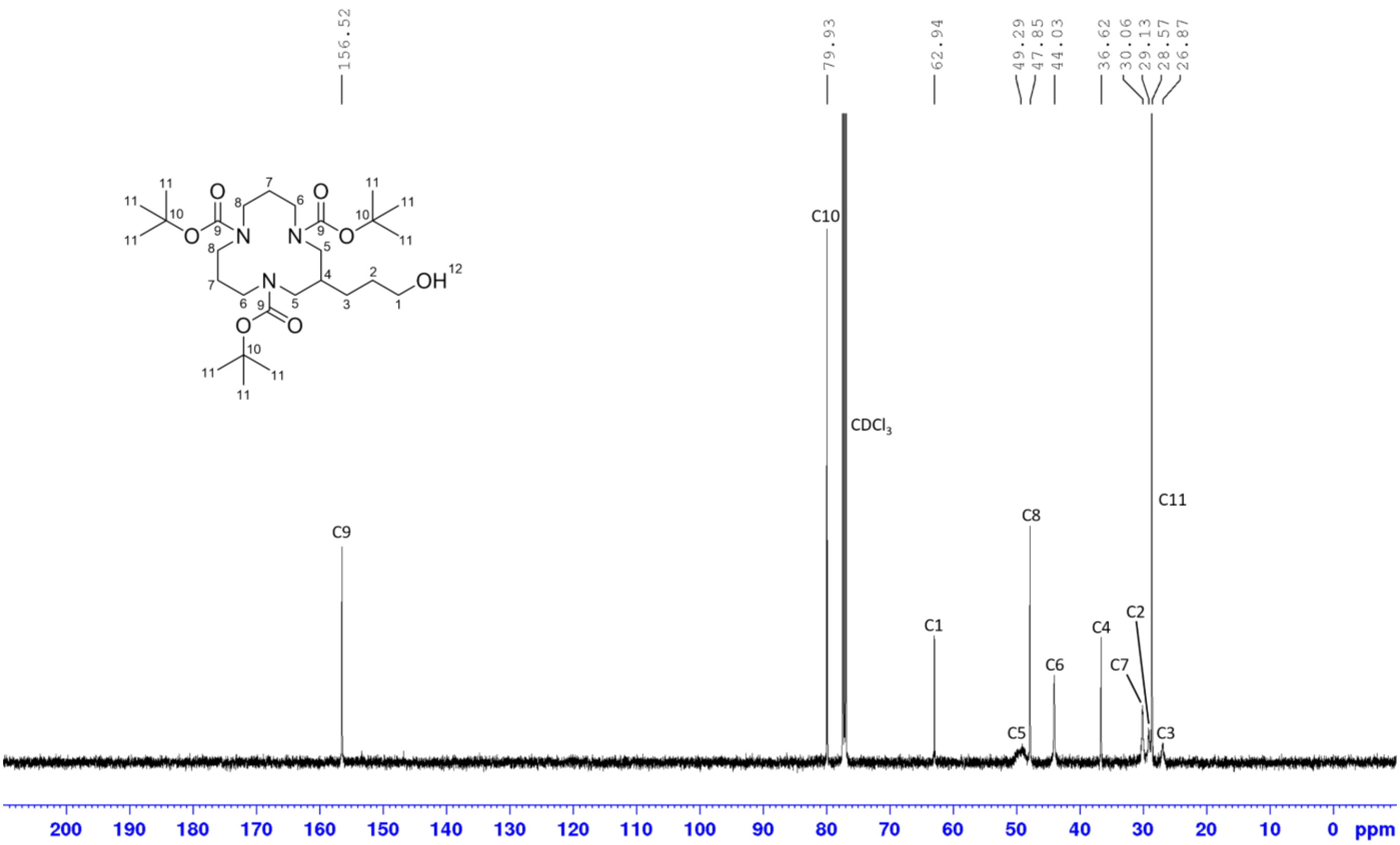

Figure S2. $^{13}C\{^{1}H\}$ NMR spectrum of **6** ($CDCl_3$, 126 MHz, 25 °C).

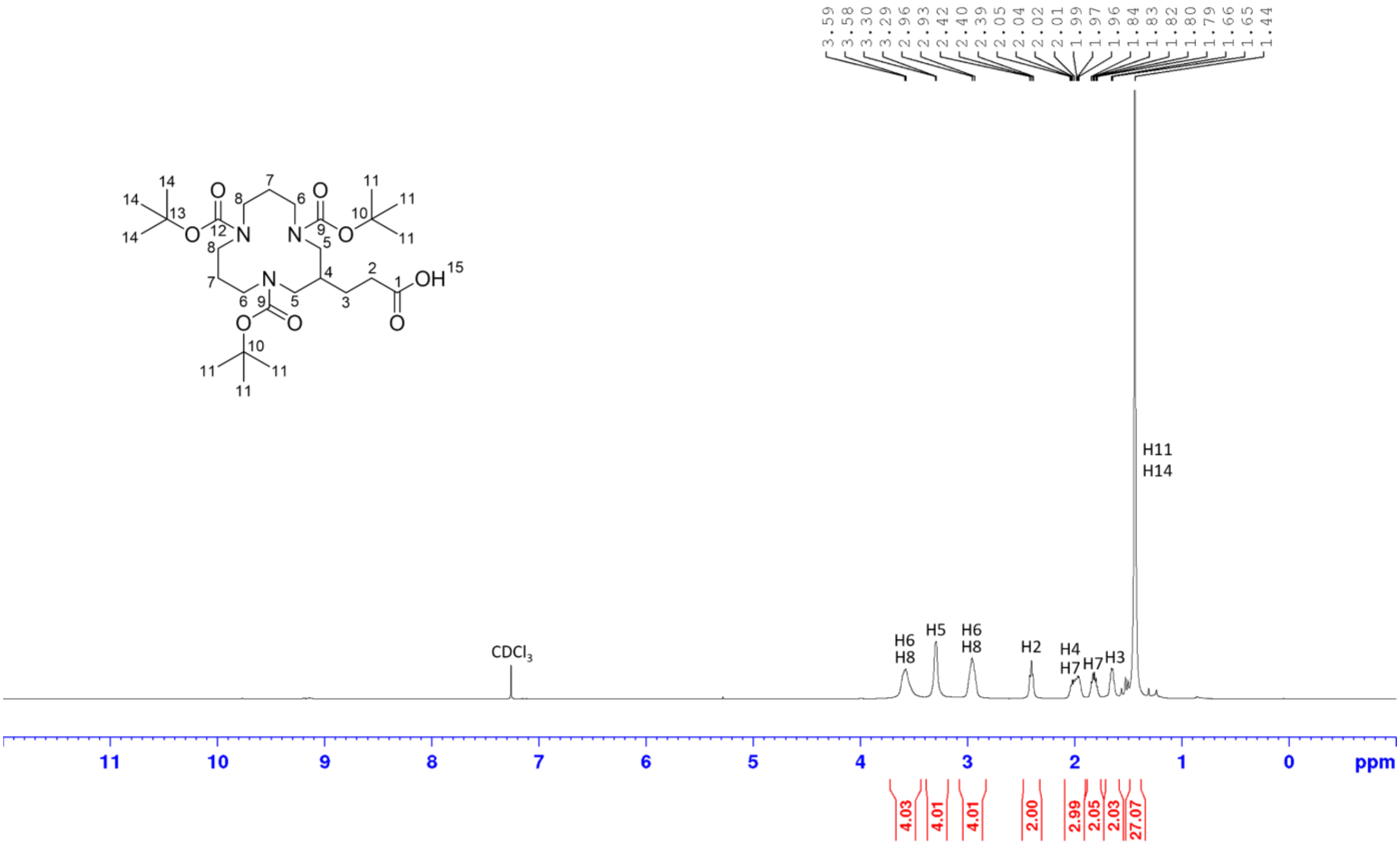

Figure S3. ^{1}H NMR spectrum of **4** ($CDCl_3$, 500 MHz, 25 °C).

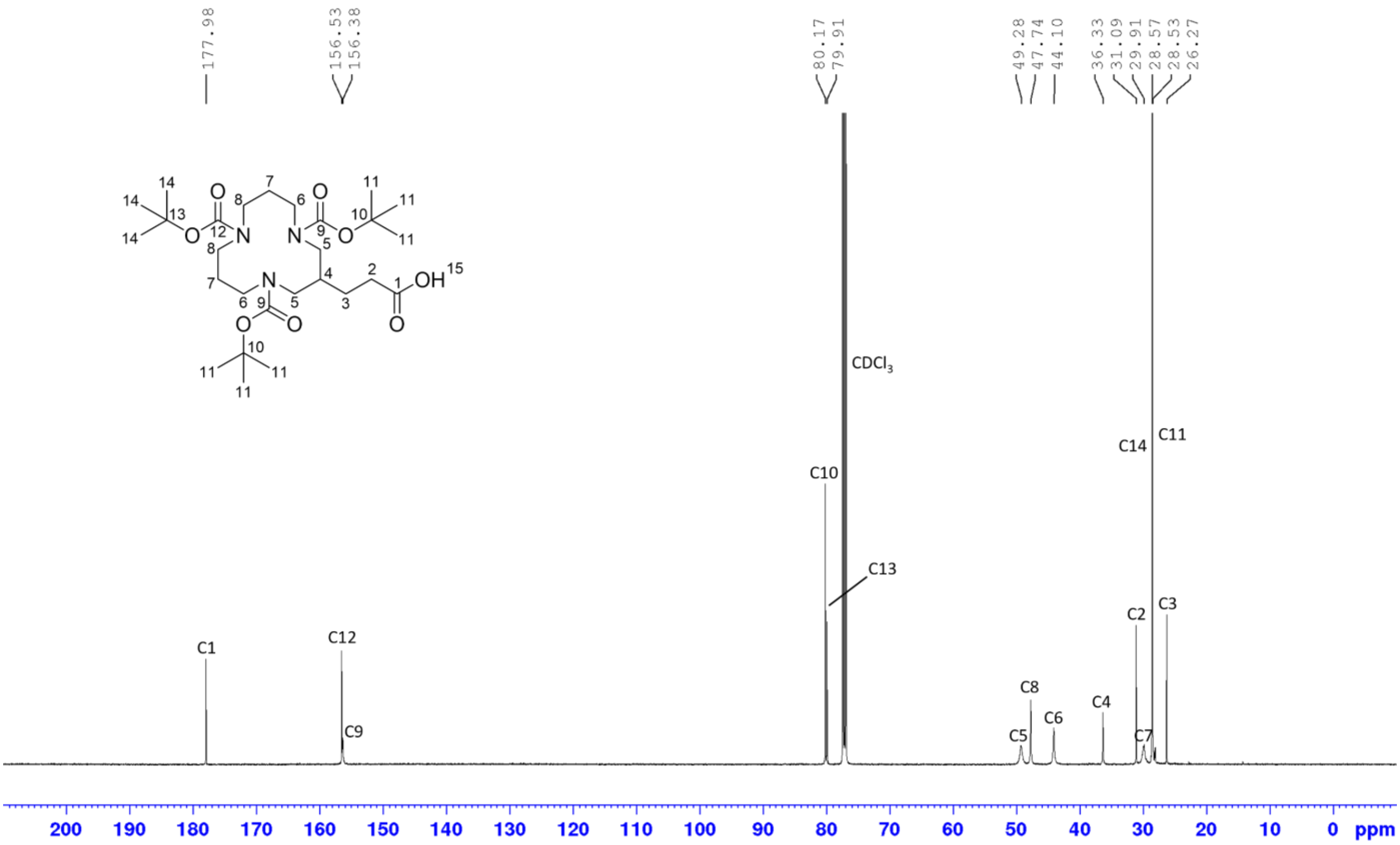

Figure S4. $^{13}C\{^{1}H\}$ NMR spectrum of **4** ($CDCl_3$, 126 MHz, 25 °C).

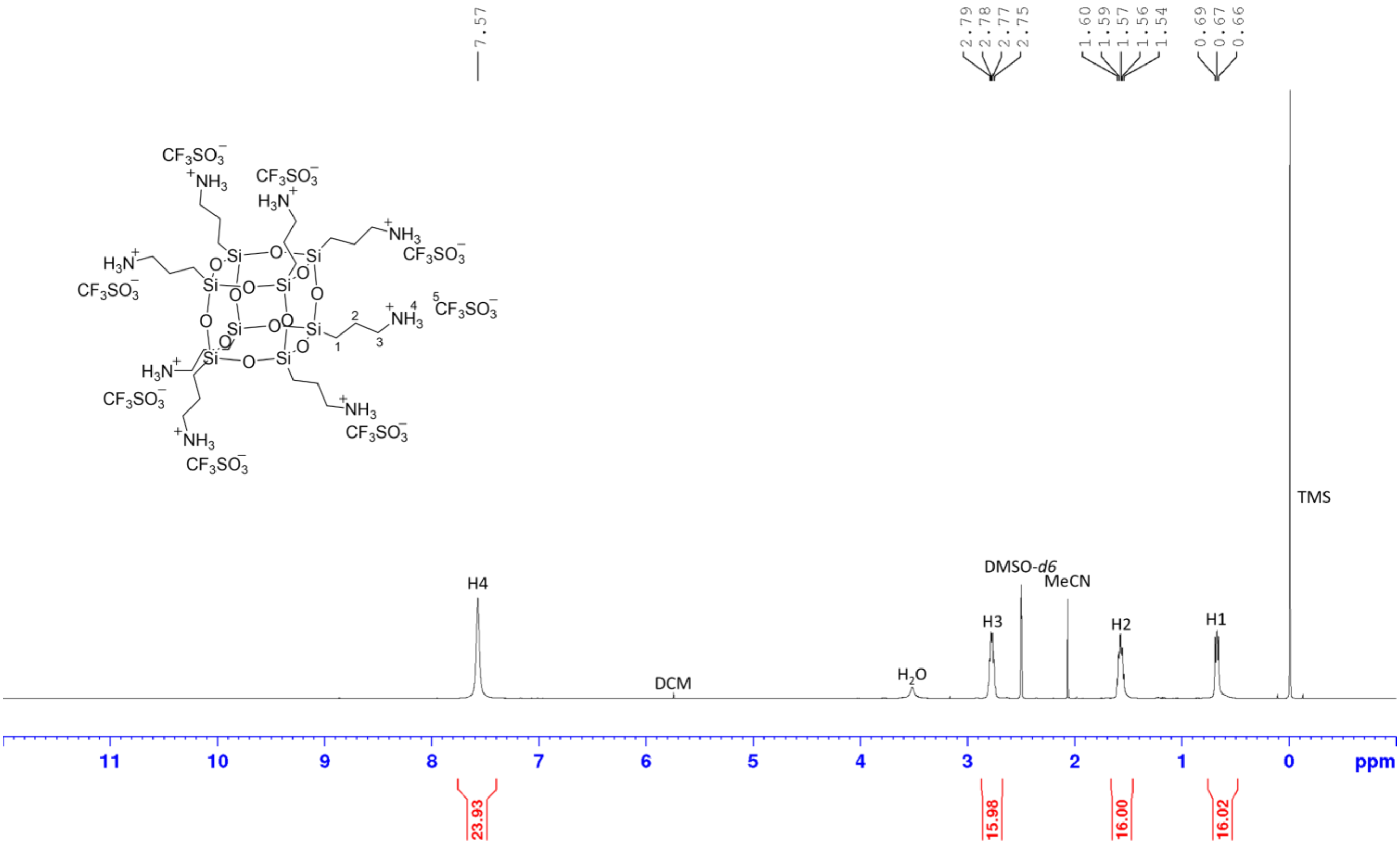

Figure S5. ^{1}H NMR spectrum of **7** (DMSO-*d6*, 500 MHz, 25 °C).

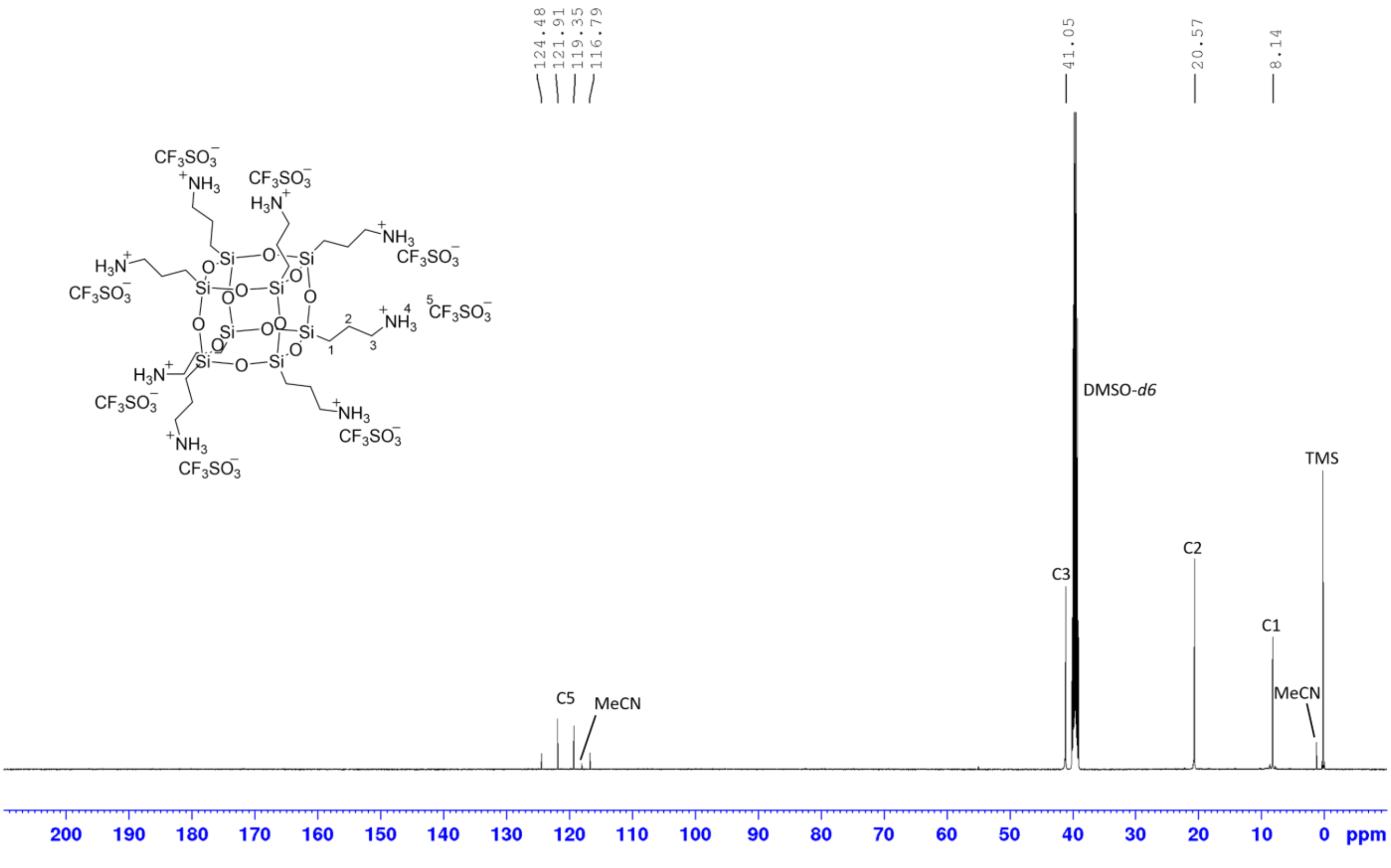

Figure S6. $^{13}C\{^{1}H\}$ NMR spectrum of **7** (DMSO-*d6*, 126 MHz, 25 °C).

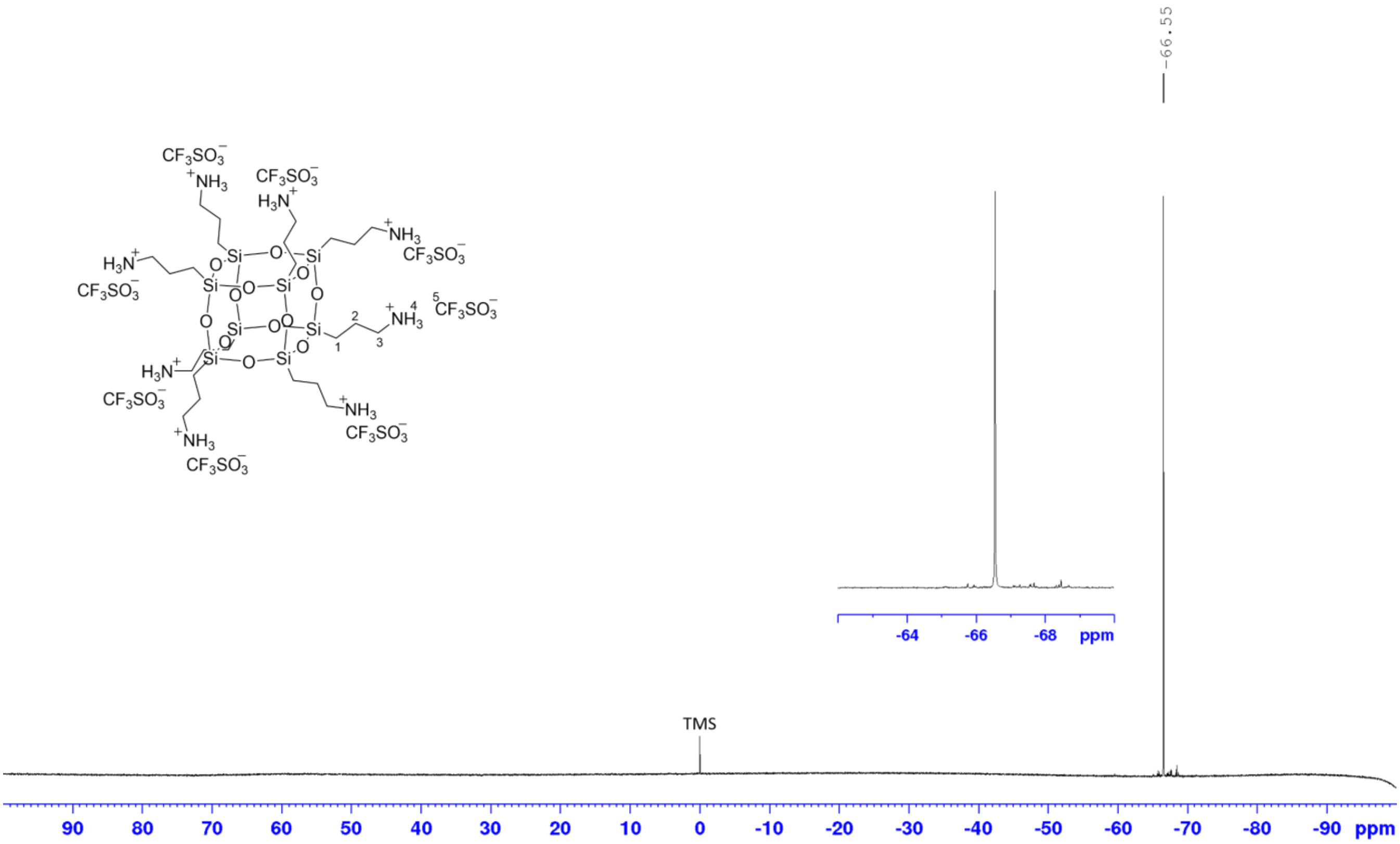

Figure S7. $^{29}Si\{^{1}H\}$ NMR spectrum of **7** (DMSO-*d6*, 99 MHz, 25 °C).

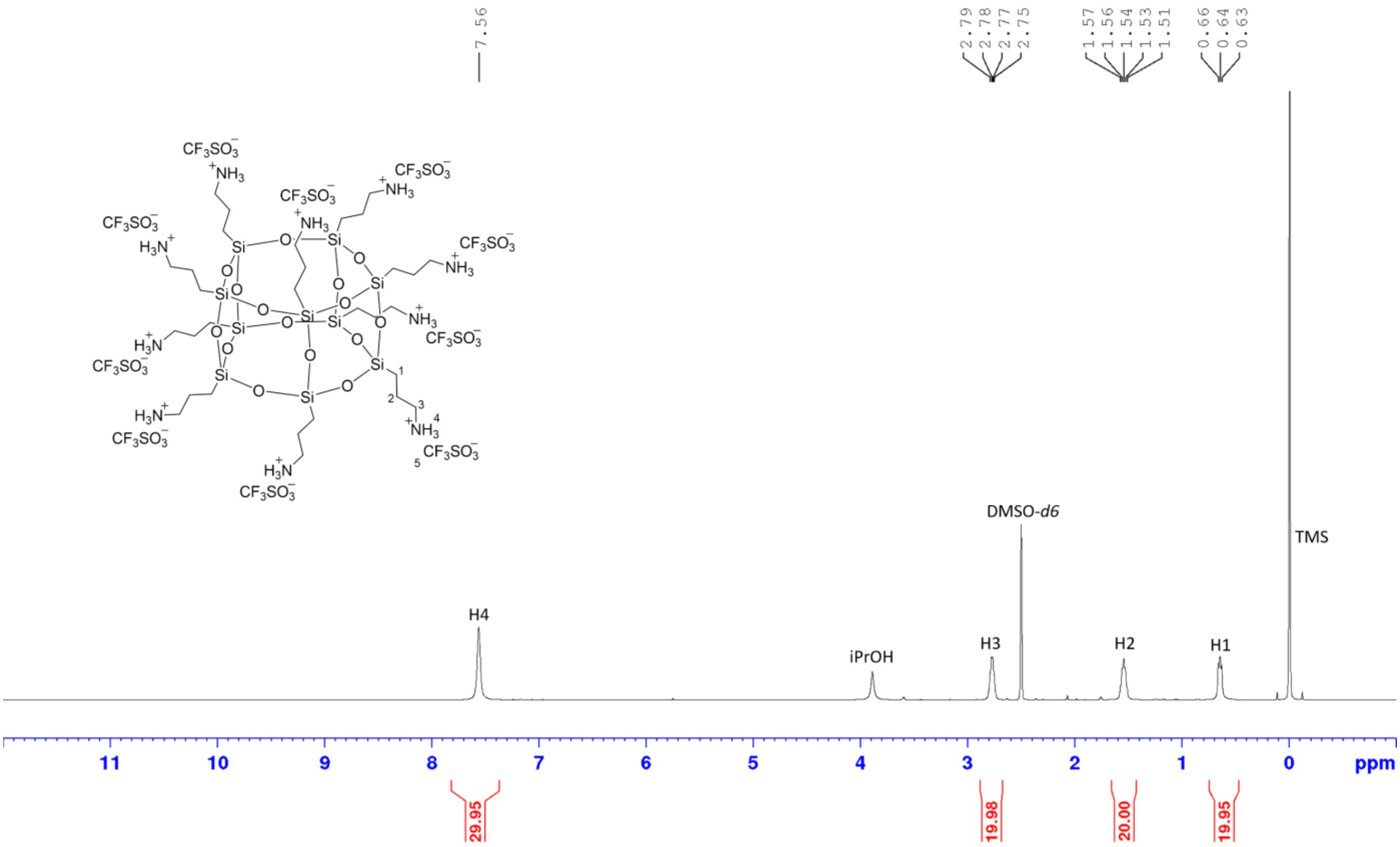

Figure S8. ^{1}H NMR spectrum of **16** (DMSO-*d6*, 500 MHz, 25 °C).

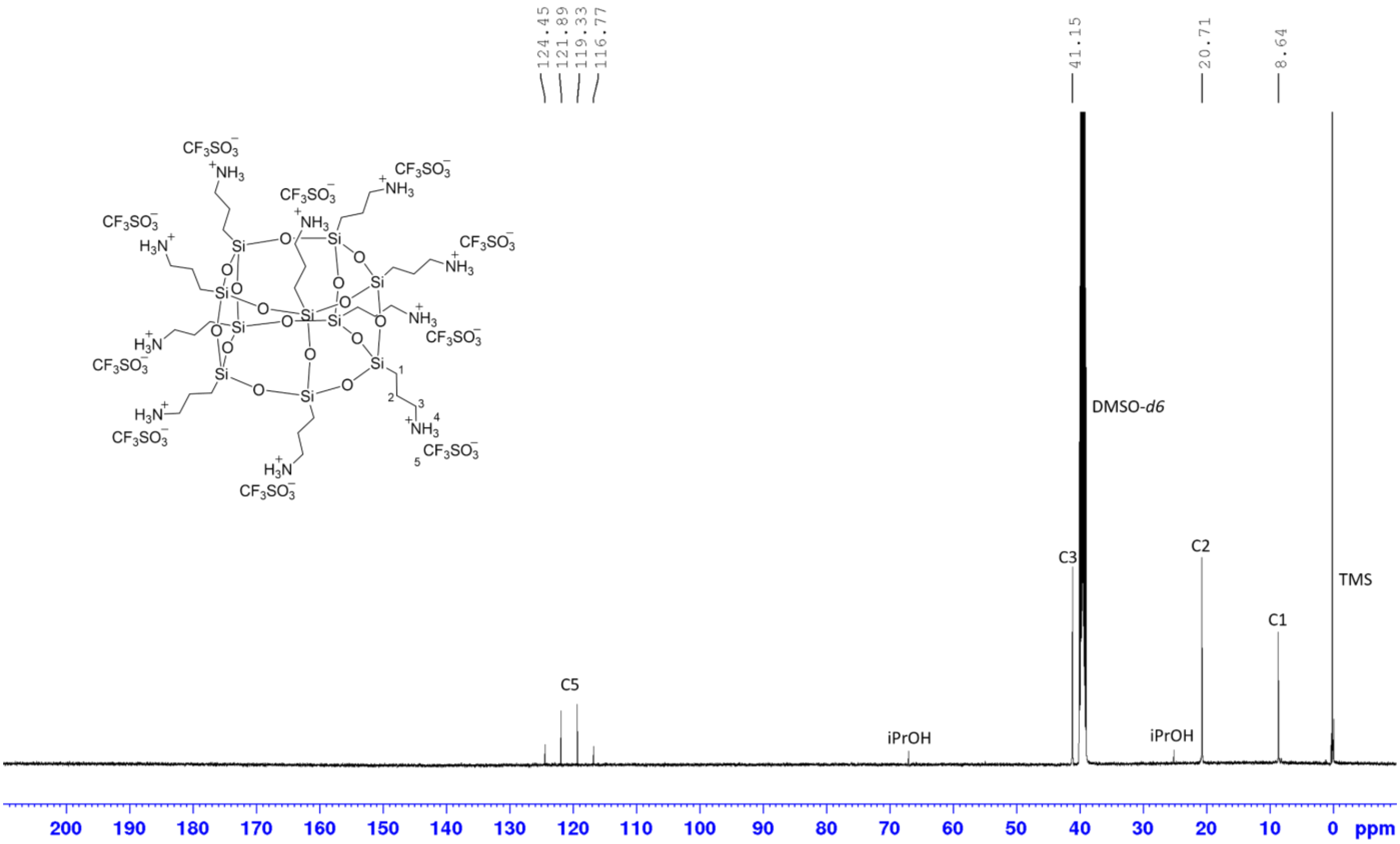

Figure S9. $^{13}C\{^1H\}$ NMR spectrum of **16** (DMSO-*d6*, 126 MHz, 25 °C).

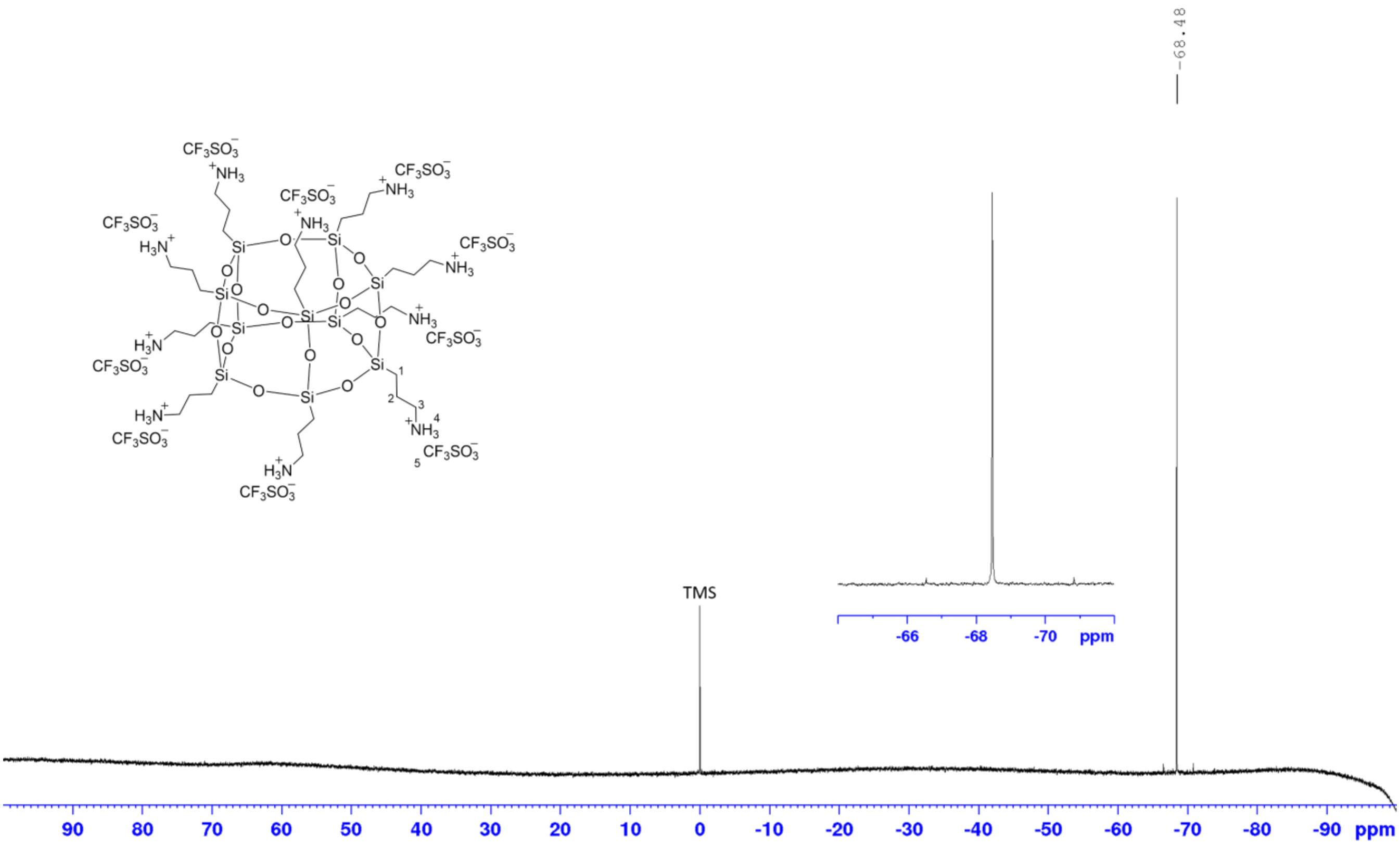

Figure S10. $^{29}Si\{^1H\}$ NMR spectrum of **16** (DMSO-*d6*, 99 MHz, 25 °C).

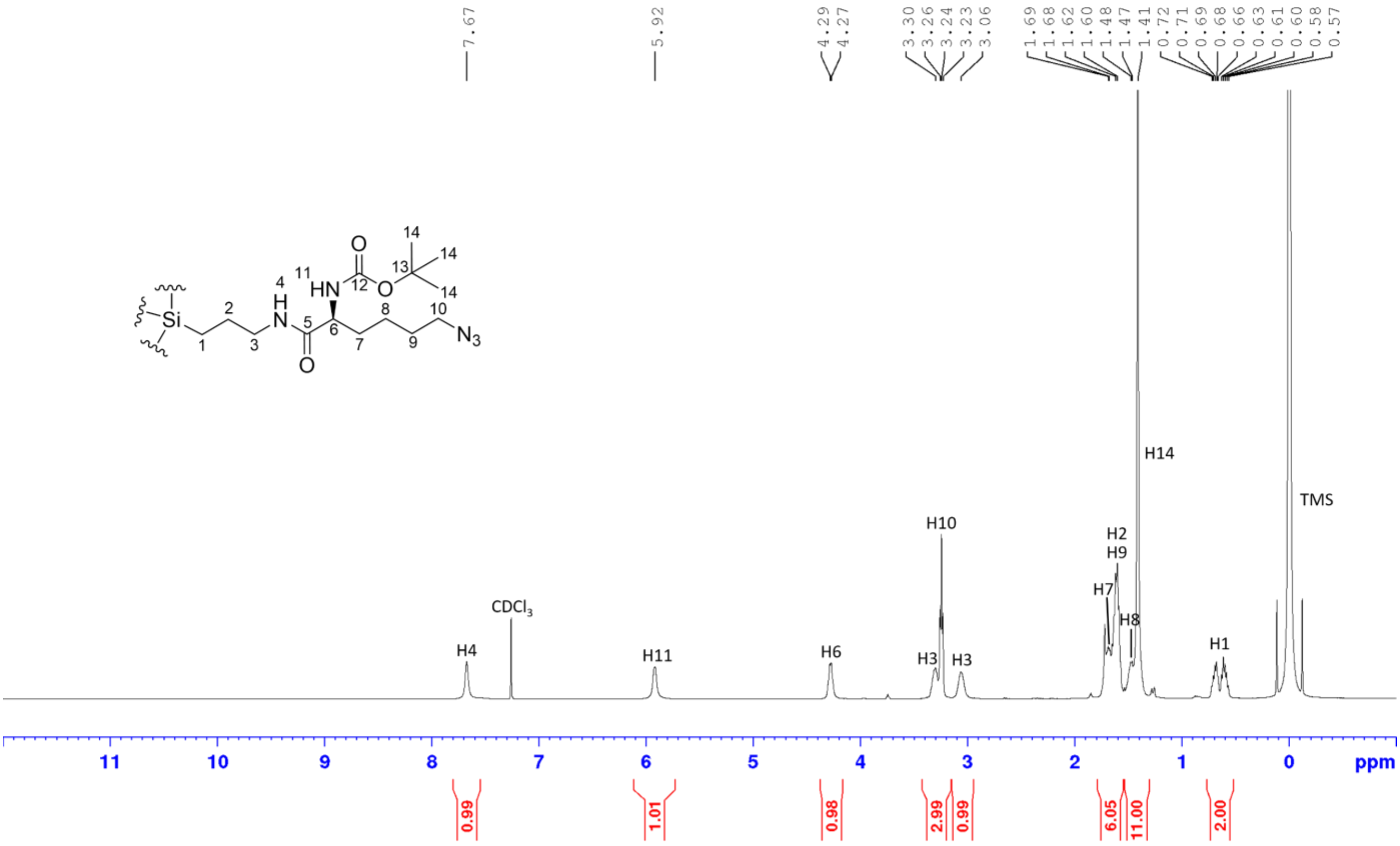

Figure S11. ^{1}H NMR spectrum of **8** ($CDCl_3$, 500 MHz, 25 °C).

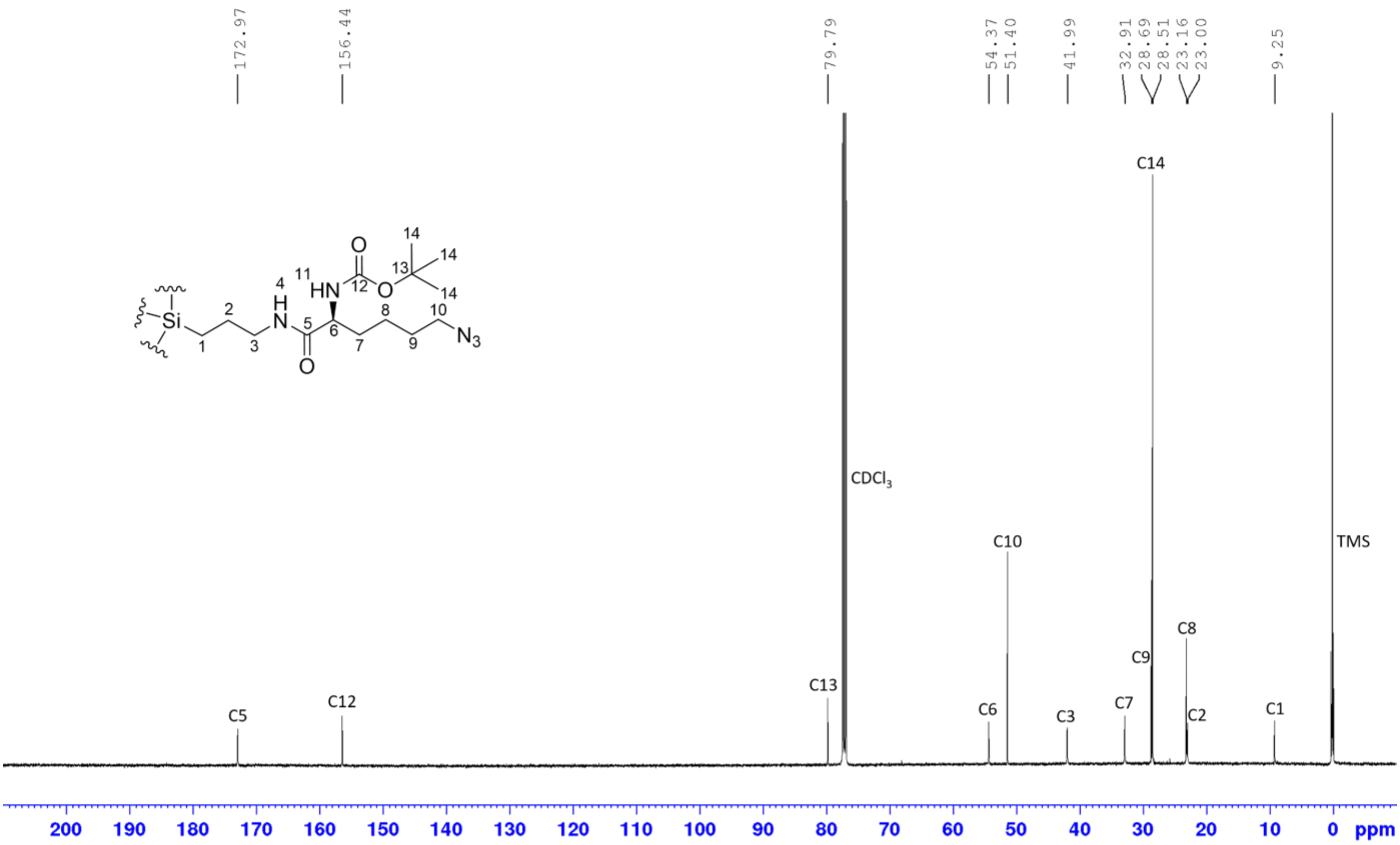

Figure S12. $^{13}C\{^{1}H\}$ NMR spectrum of **8** ($CDCl_3$, 126 MHz, 25 °C).

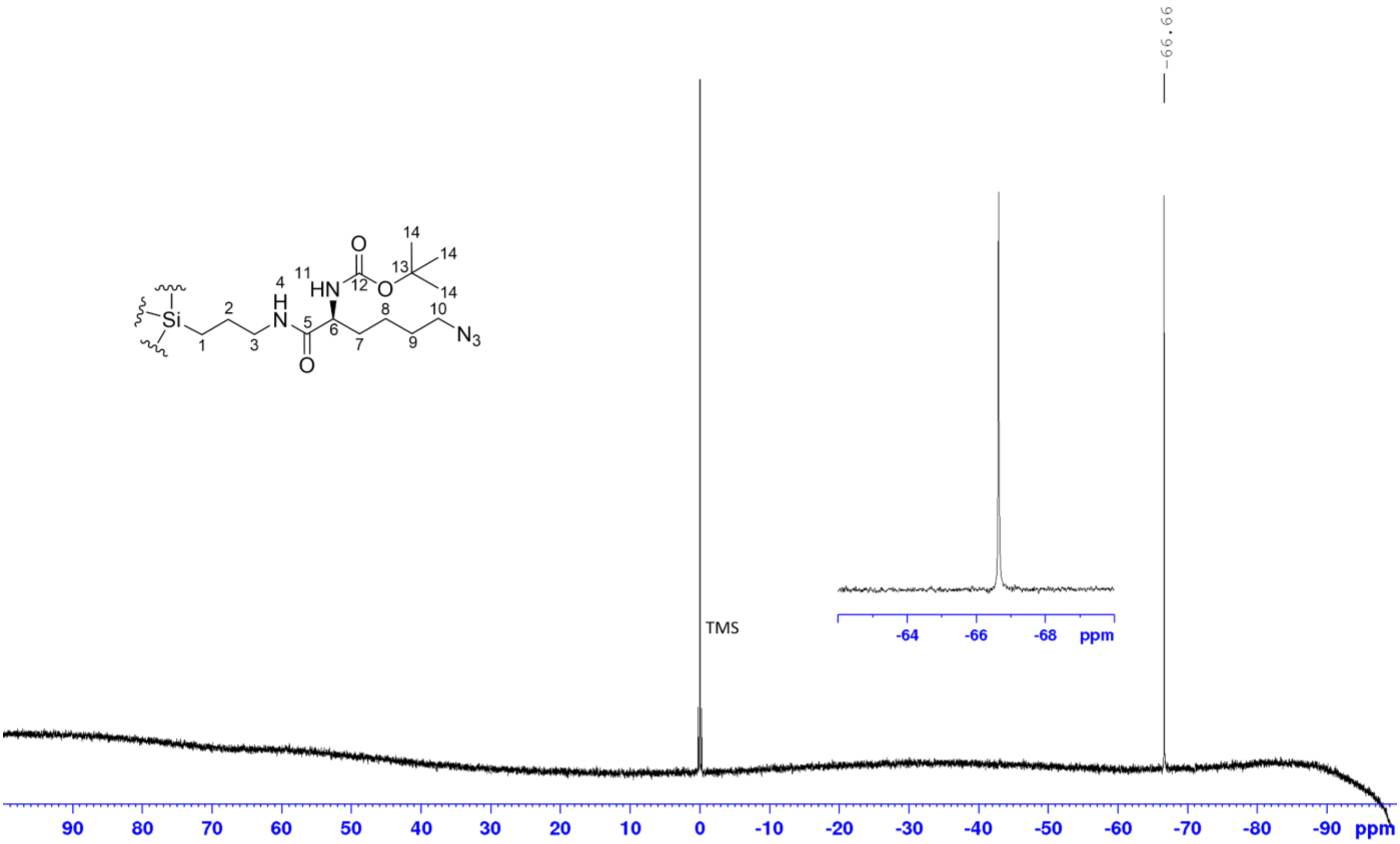

Figure S13. $^{29}Si\{^{1}H\}$ NMR spectrum of **8** ($CDCl_3$, 99 MHz, 25 °C).

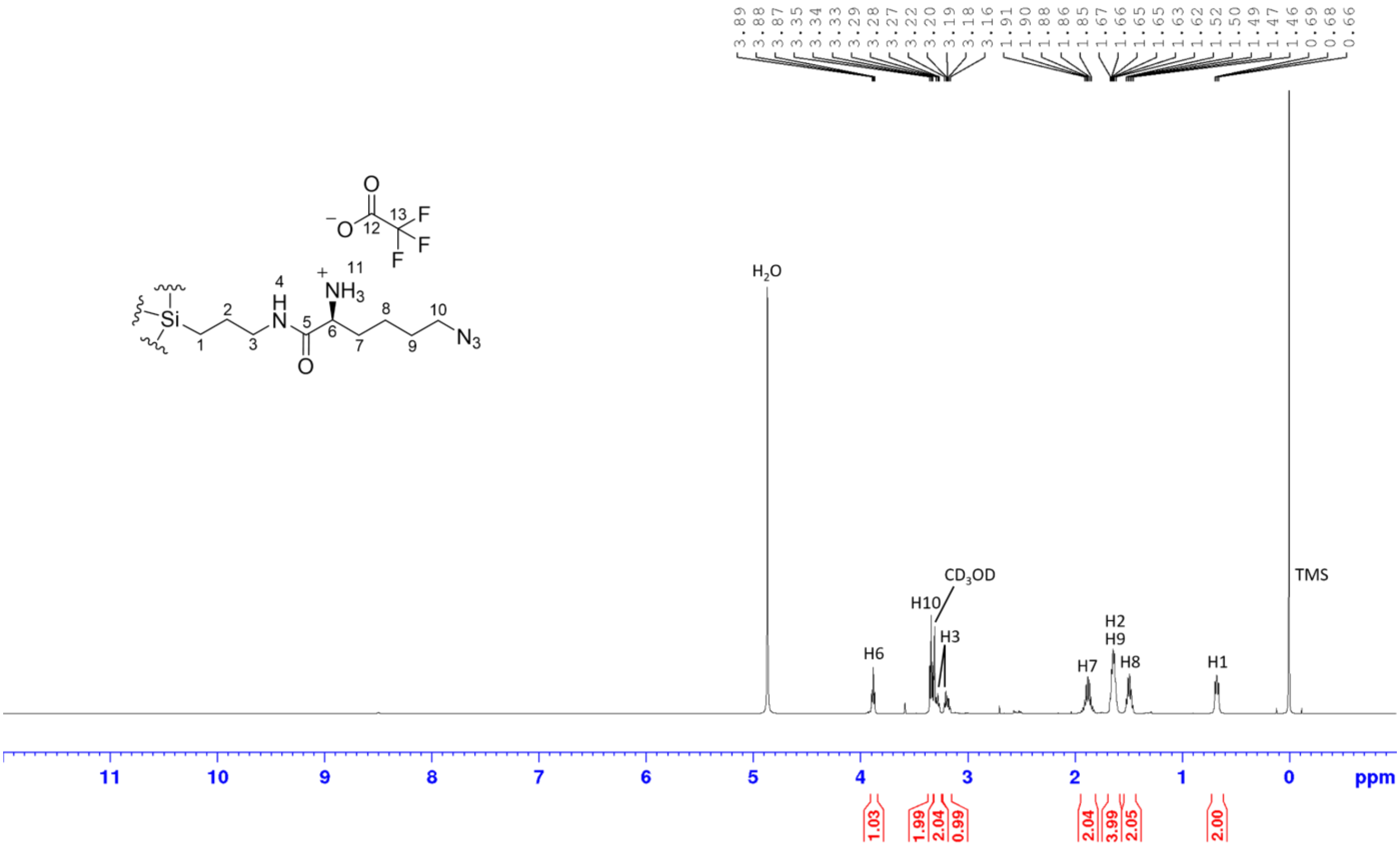

Figure S14. ^{1}H NMR spectrum of **10** (CD_3OD, 500 MHz, 25 °C).

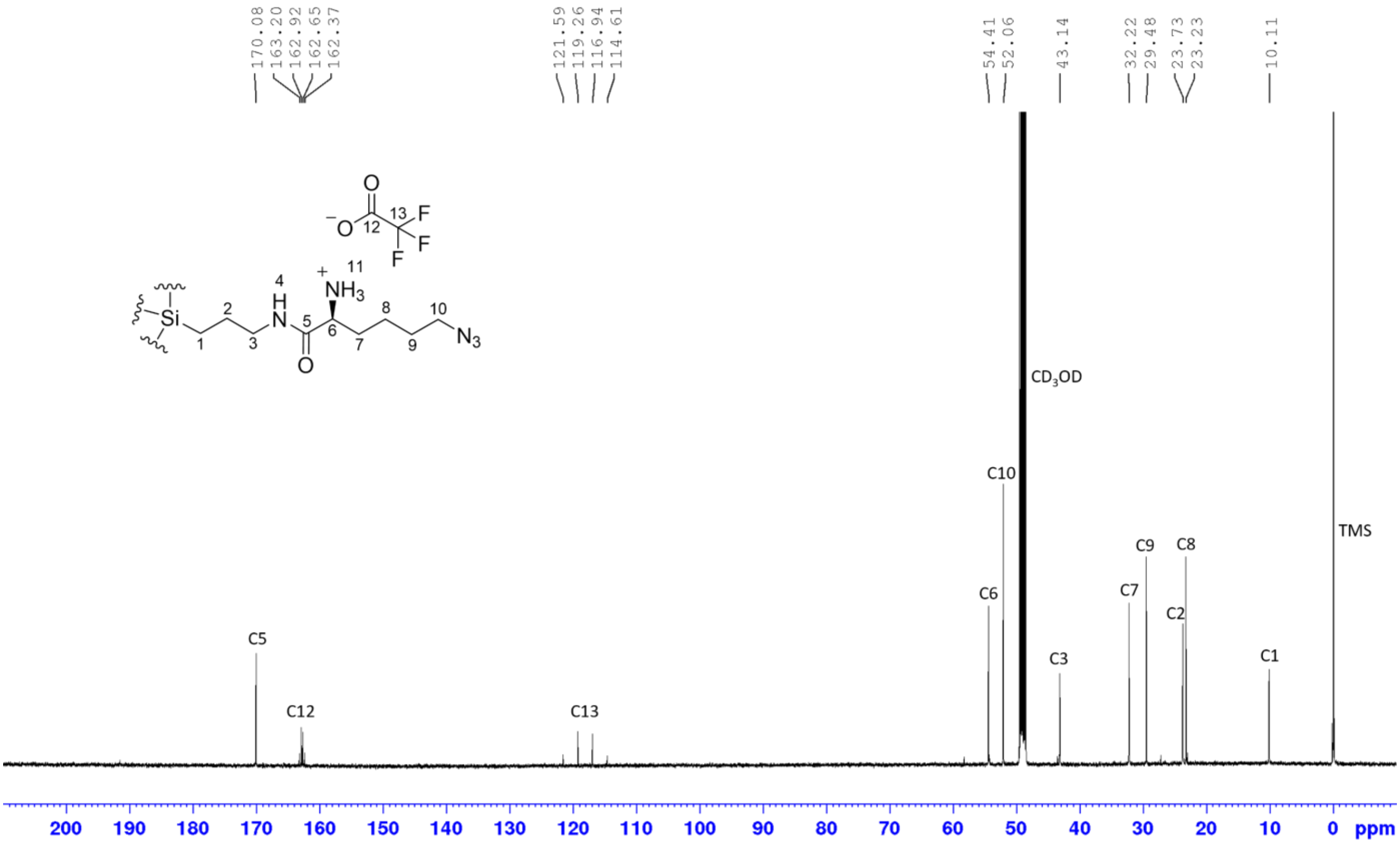

Figure S15. $^{13}C\{^1H\}$ NMR spectrum of **10** (CD_3OD, 126 MHz, 25 °C).

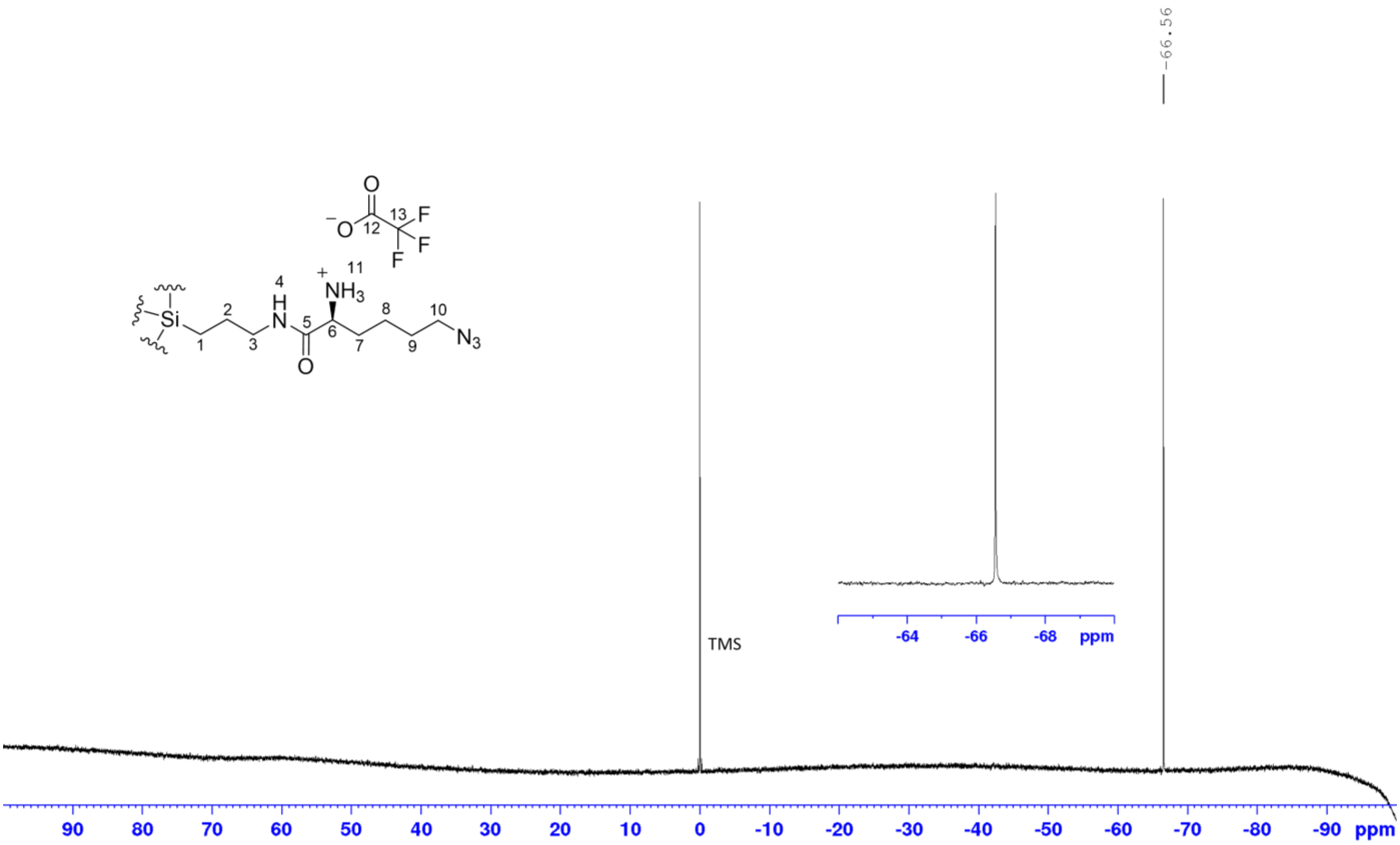

Figure S16. $^{29}Si\{^1H\}$ NMR spectrum of **10** (CD_3OD, 99 MHz, 25 °C).

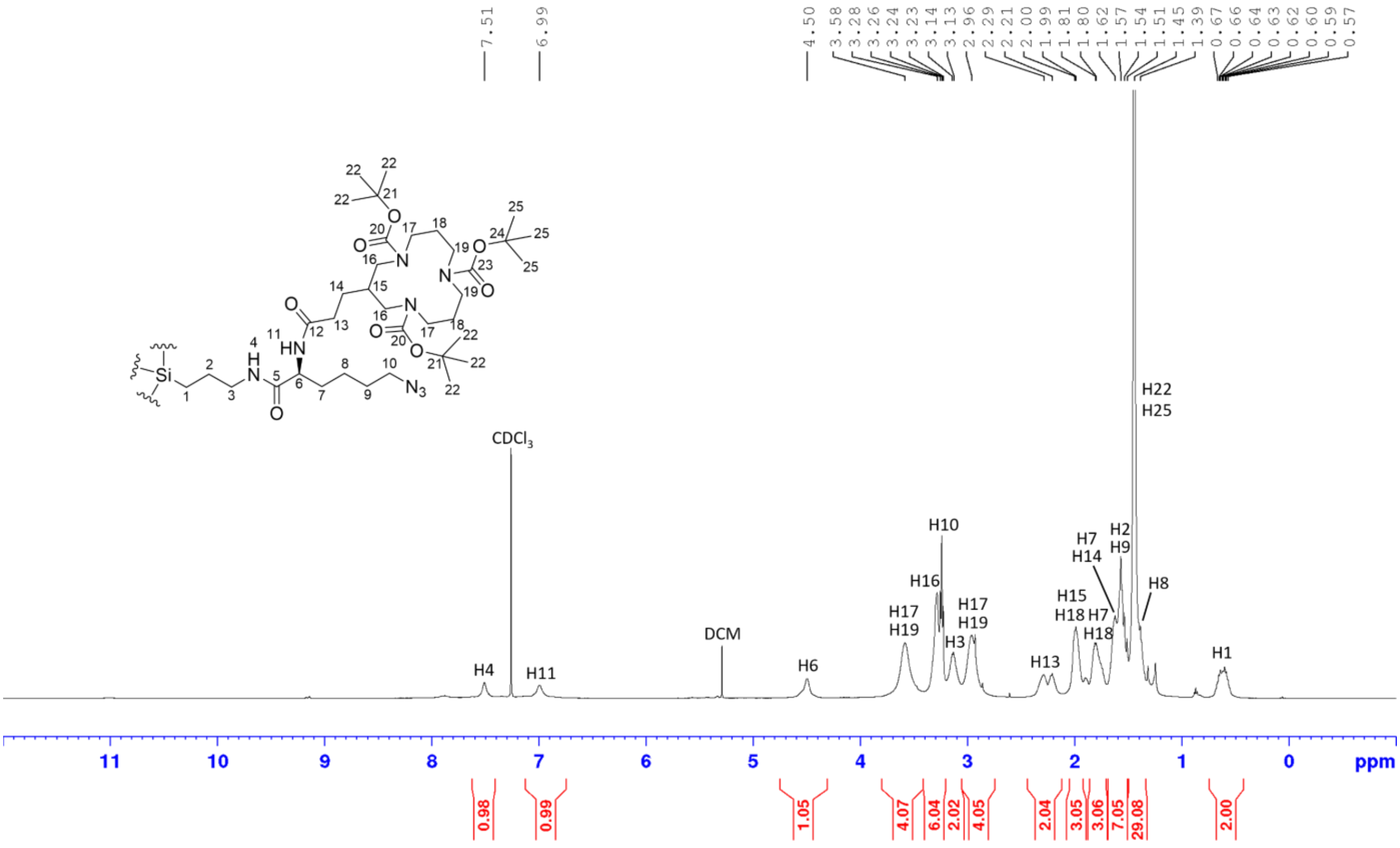

Figure S17. ^{1}H NMR spectrum of **11** ($CDCl_3$, 500 MHz, 25 °C).

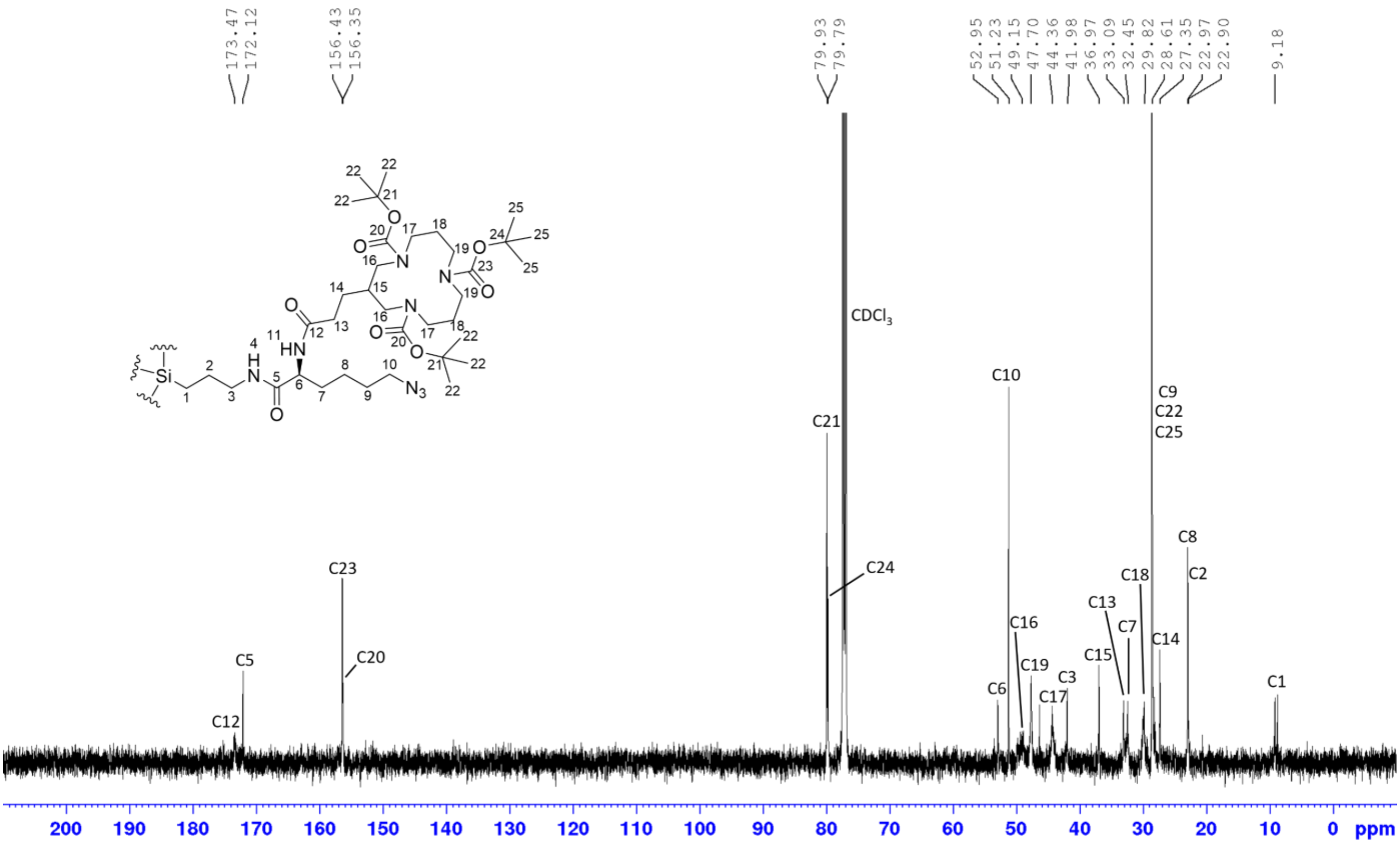

Figure S18. $^{13}C\{^{1}H\}$ NMR spectrum of **11** ($CDCl_3$, 126 MHz, 25 °C).

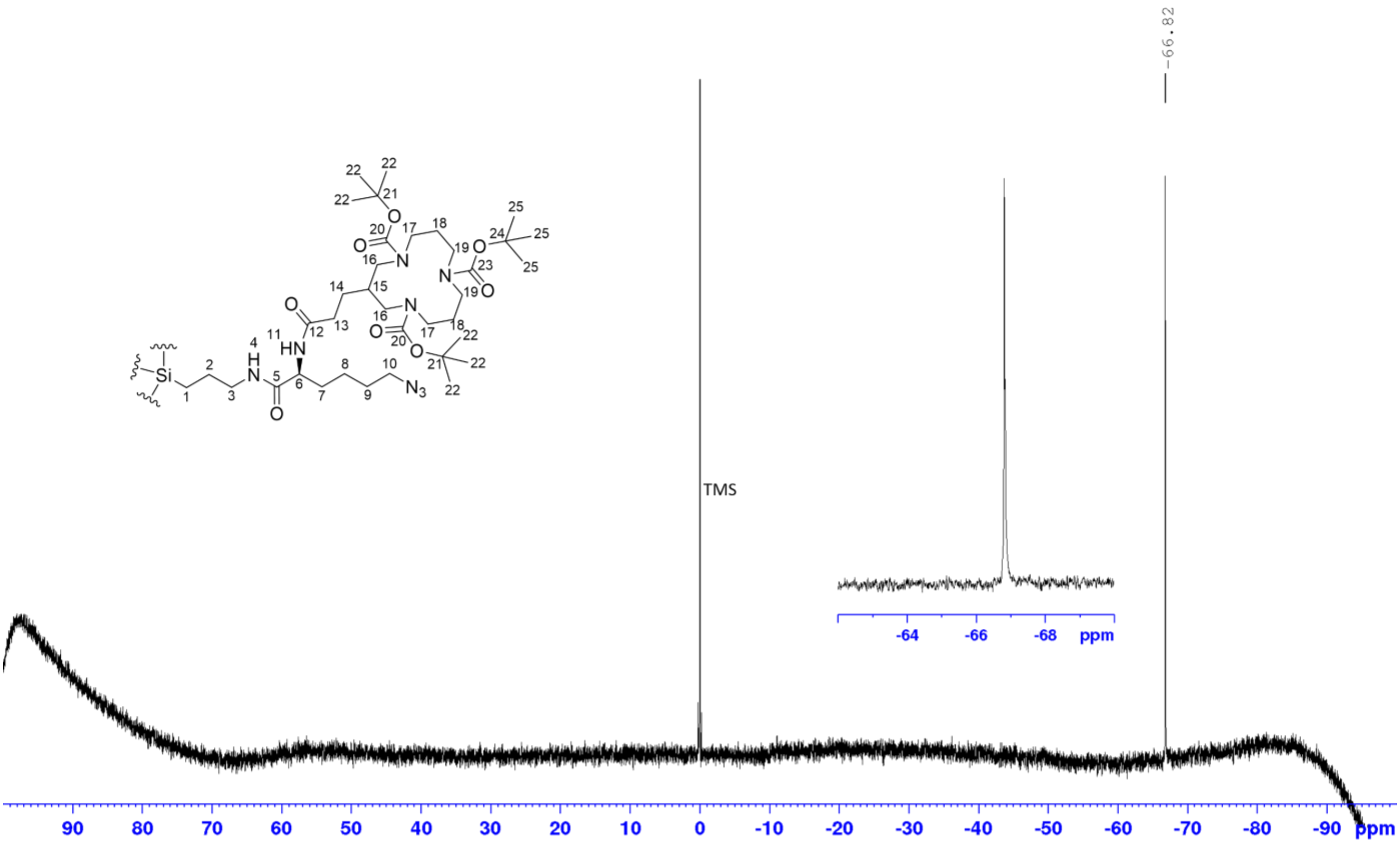

Figure S19. $^{29}Si\{^{1}H\}$ NMR spectrum of **11** ($CDCl_3$, 99 MHz, 25 °C).

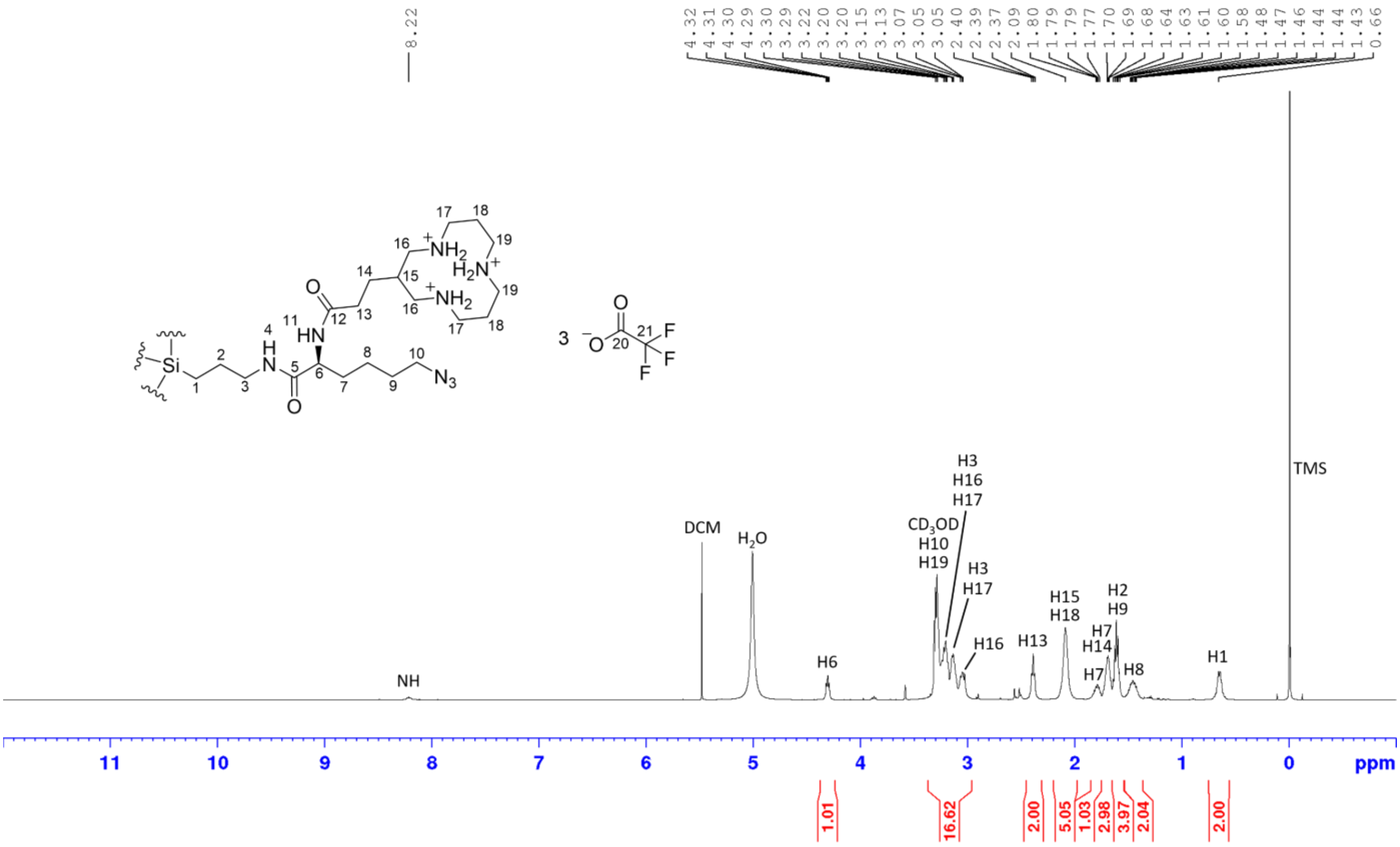

Figure S20. ^{1}H NMR spectrum of **1** (CD_3OD, 500 MHz, 25 °C).

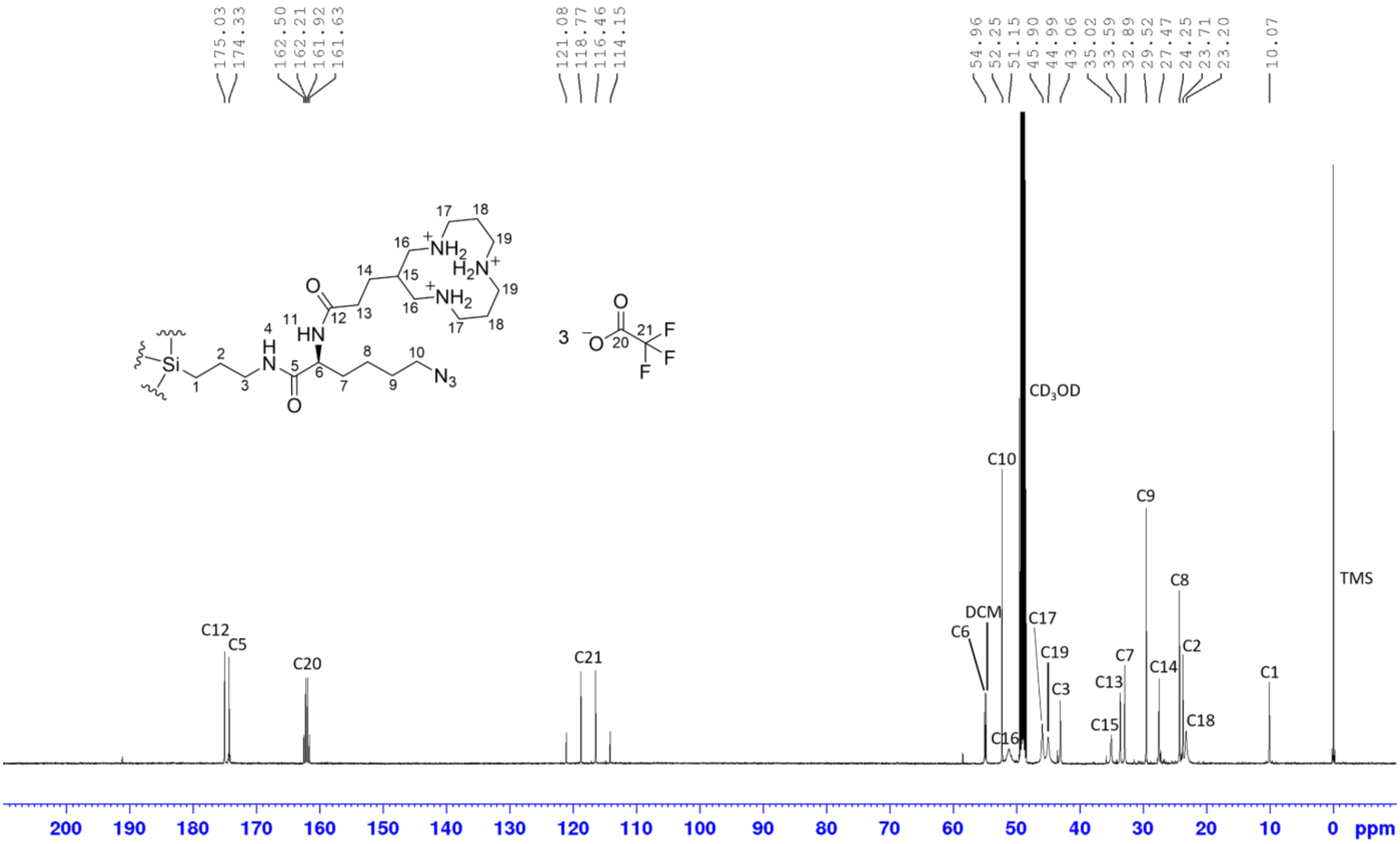

Figure S21. $^{13}C\{^1H\}$ NMR spectrum of **1** (CD_3OD, 126 MHz, 25 °C).

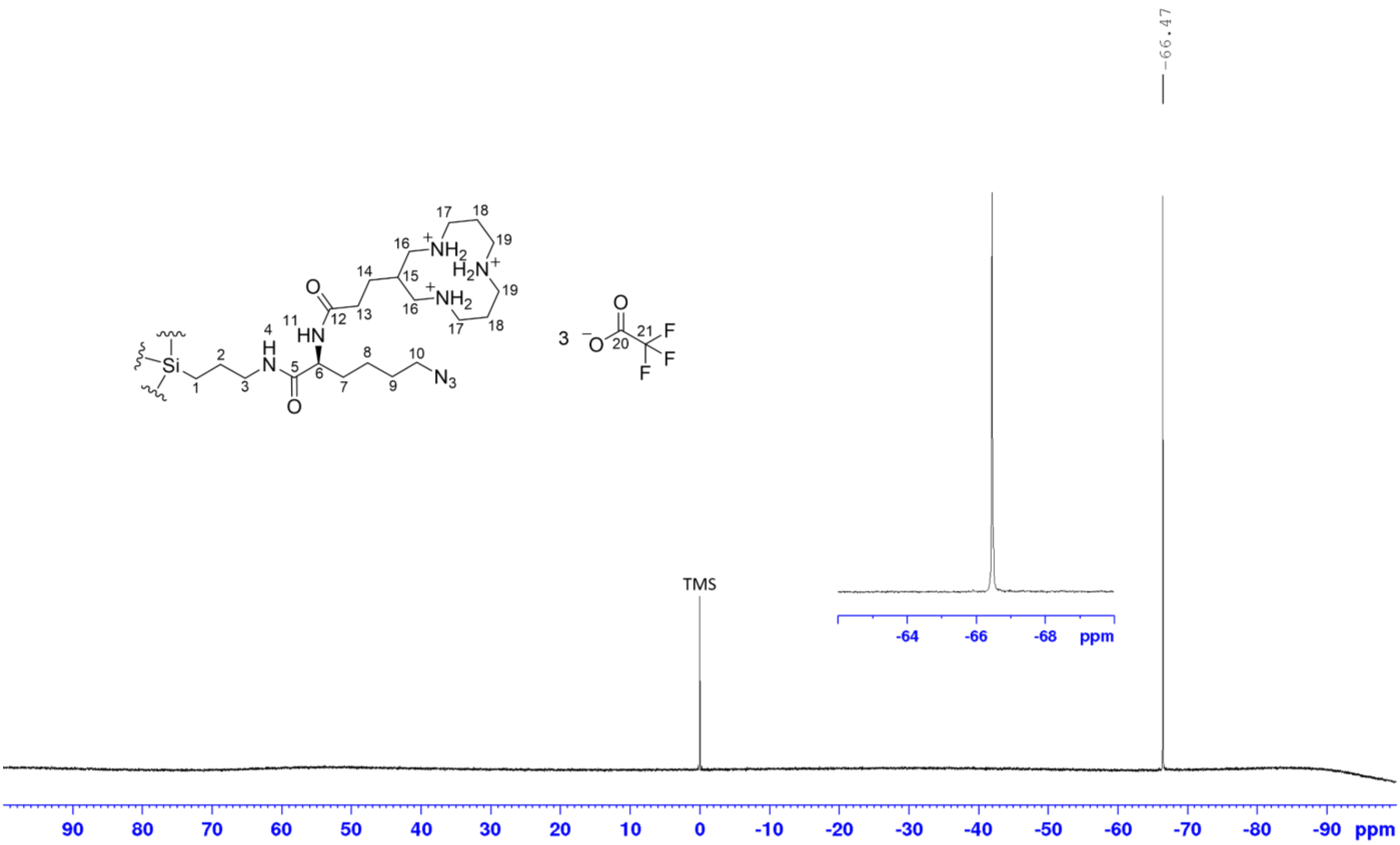

Figure S22. $^{29}Si\{^1H\}$ NMR spectrum of **1** (CD_3OD, 99 MHz, 25 °C).

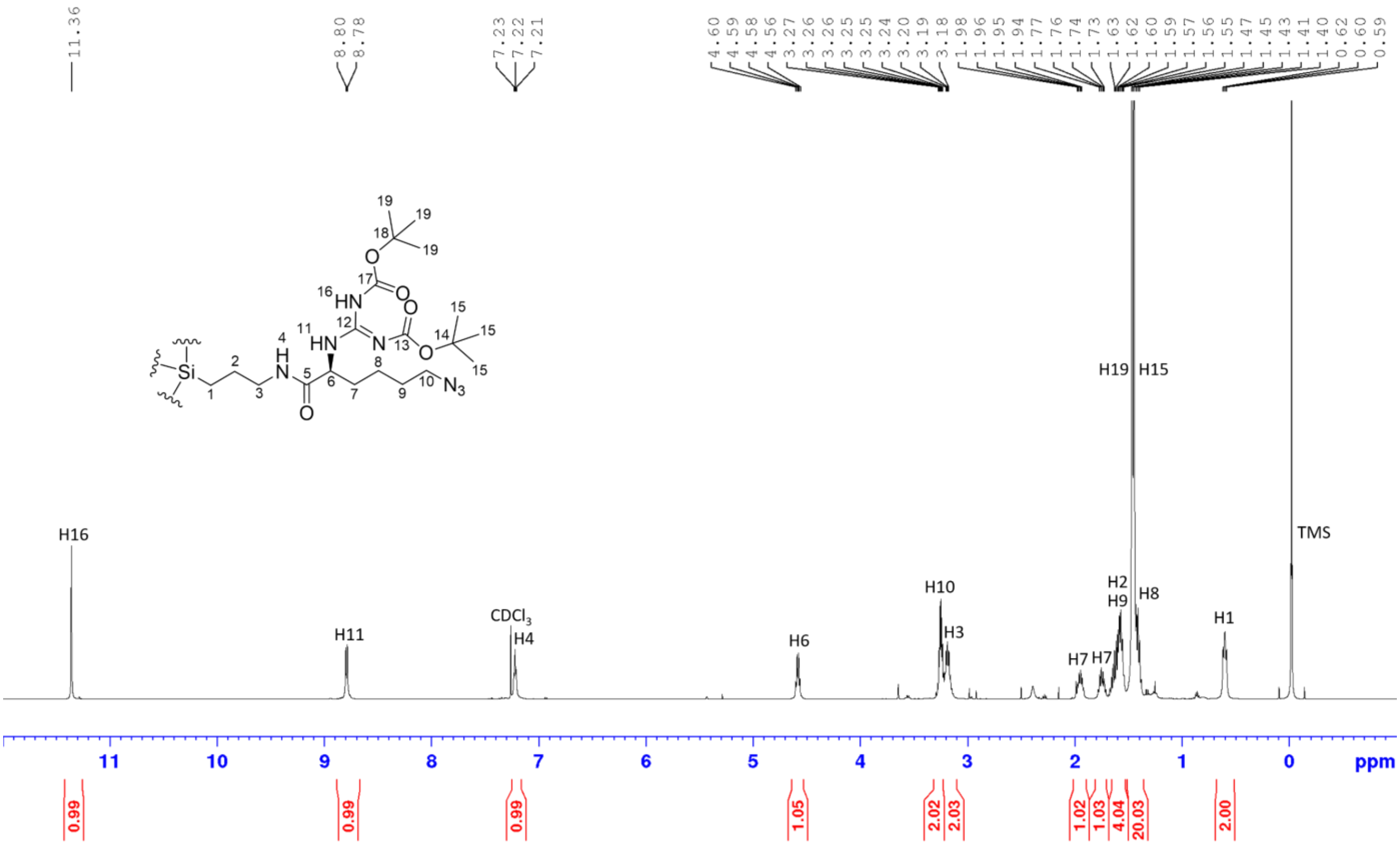

Figure S23. ^{1}H NMR spectrum of **13** ($CDCl_3$, 500 MHz, 25 °C).

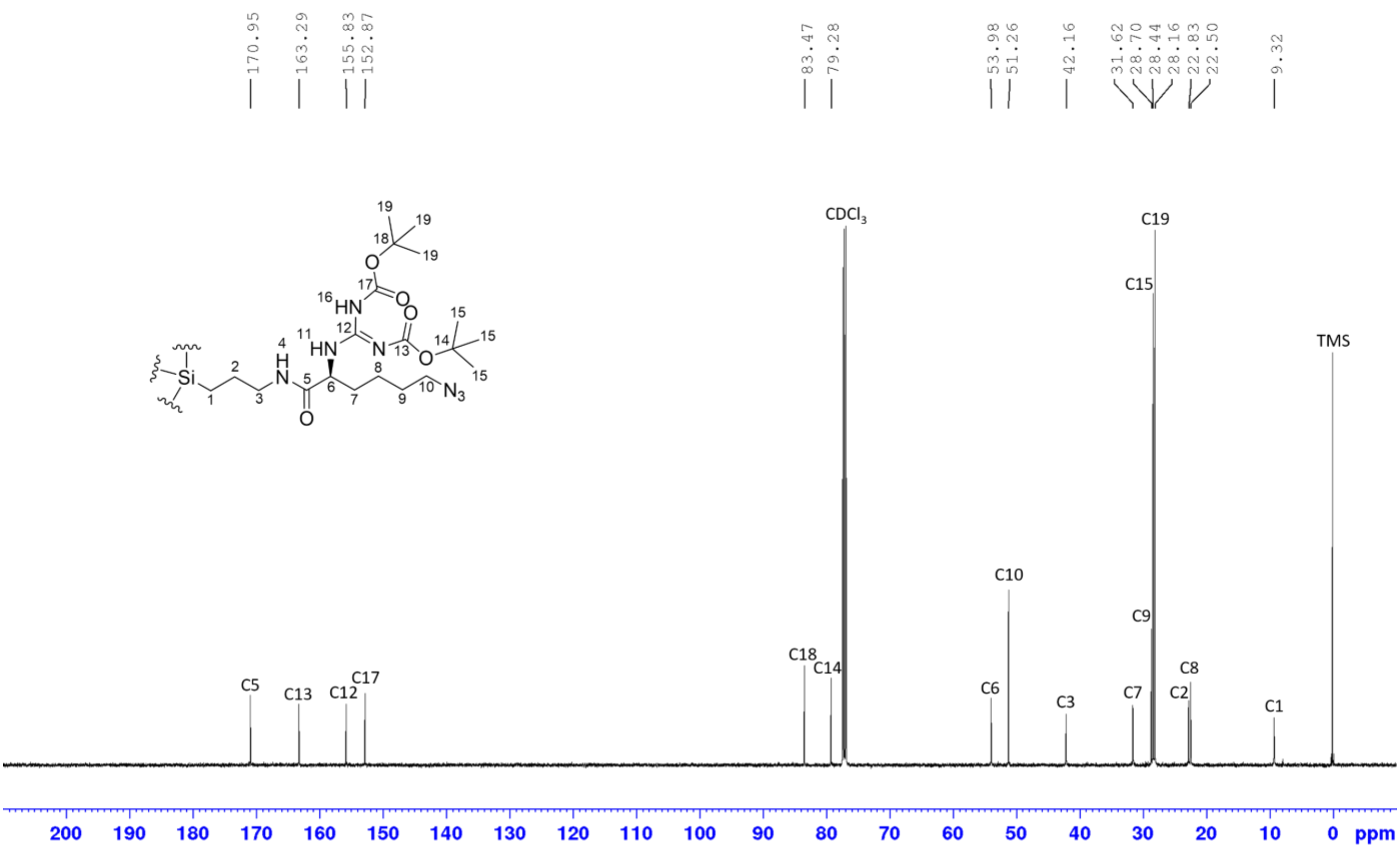

Figure S24. $^{13}C\{^{1}H\}$ NMR spectrum of **13** ($CDCl_3$, 126 MHz, 25 °C).

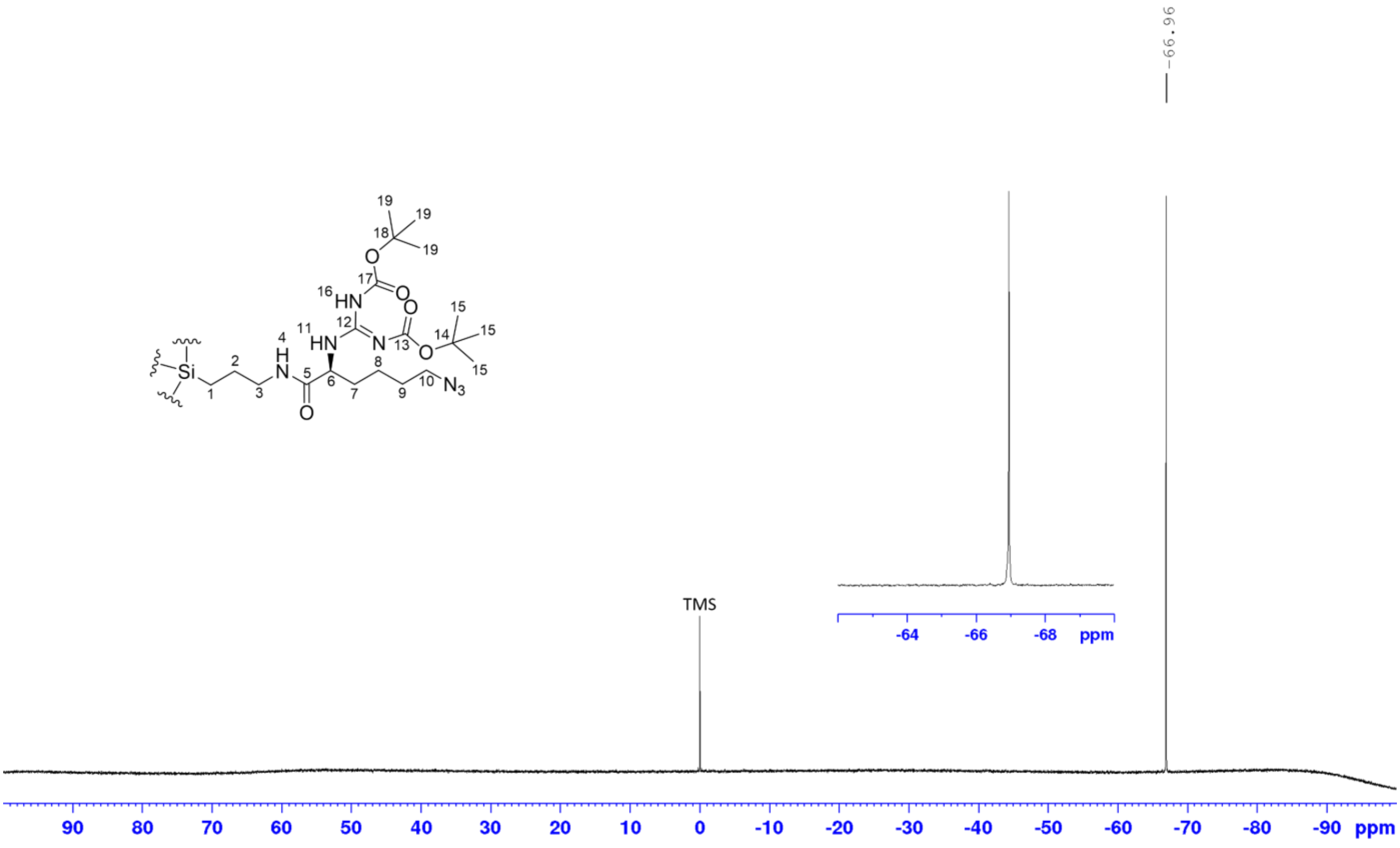

Figure S25. $^{29}Si\{^1H\}$ NMR spectrum of **13** ($CDCl_3$, 99 MHz, 25 °C).

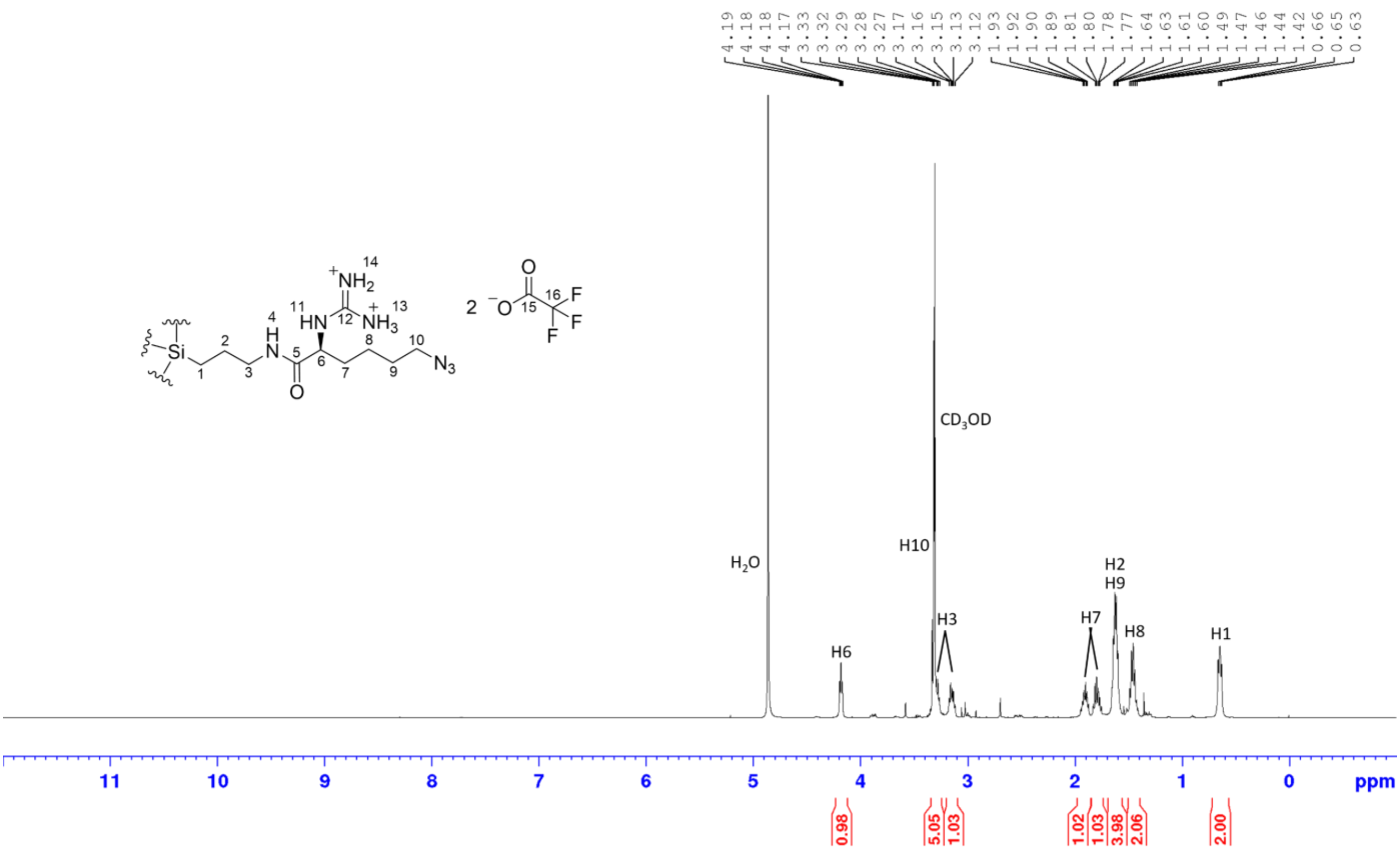

Figure S26. ^1H NMR spectrum of **2** (CD_3OD, 500 MHz, 25 °C).

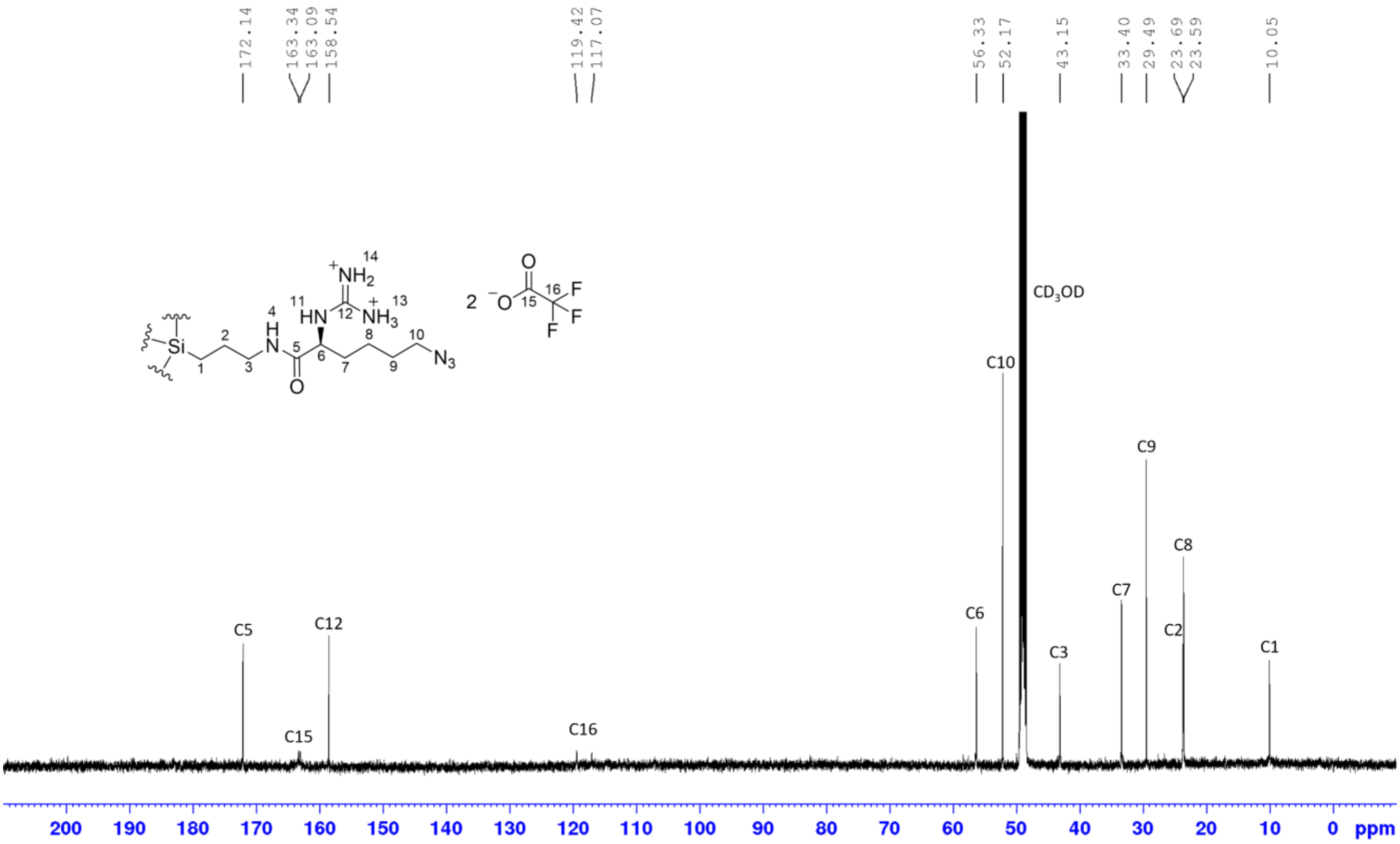

Figure S27. $^{13}C\{^1H\}$ NMR spectrum of **2** (CD_3OD, 126 MHz, 25 °C).

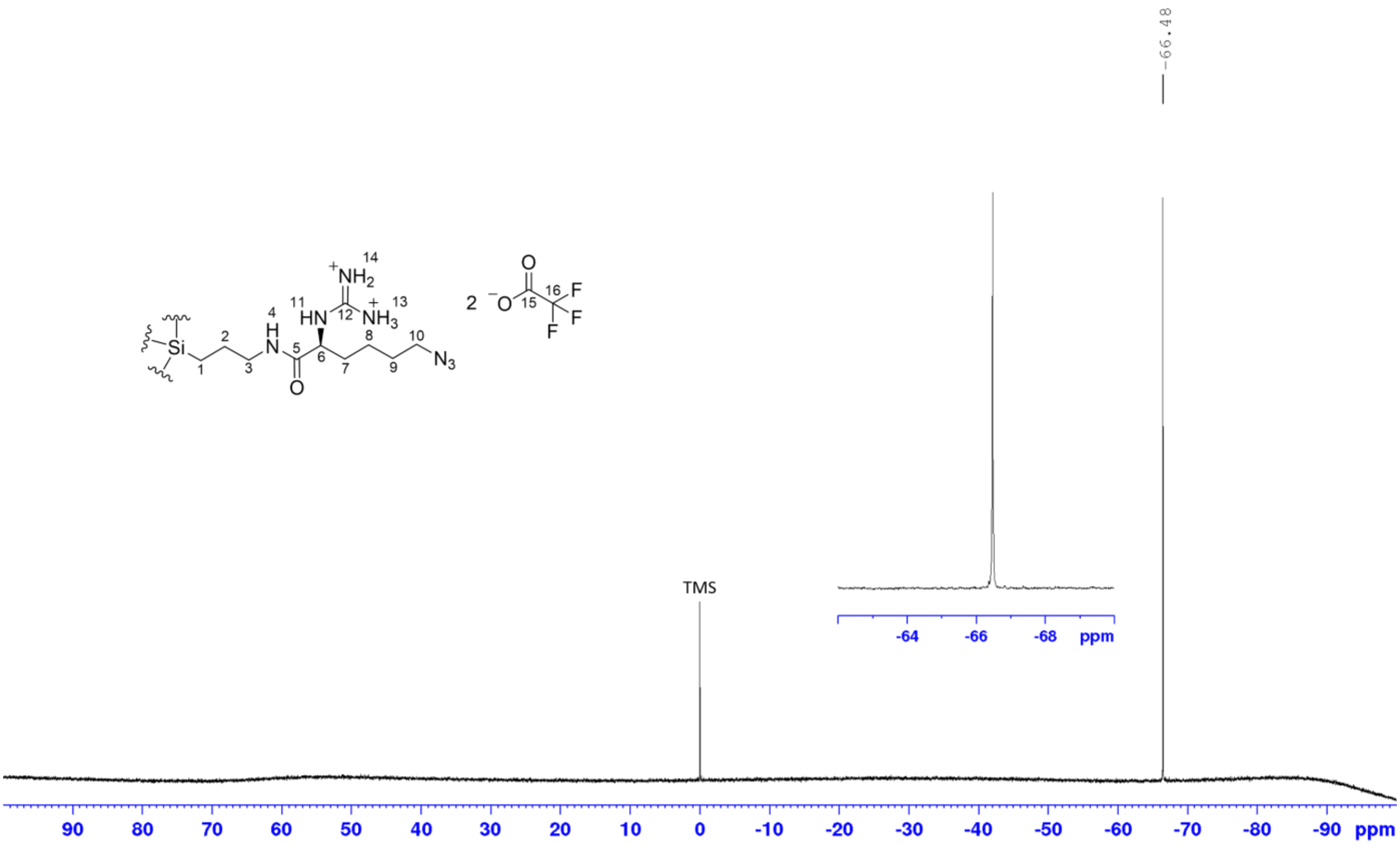

Figure S28. $^{29}Si\{^1H\}$ NMR spectrum of **2** (CD_3OD, 99 MHz, 25 °C).

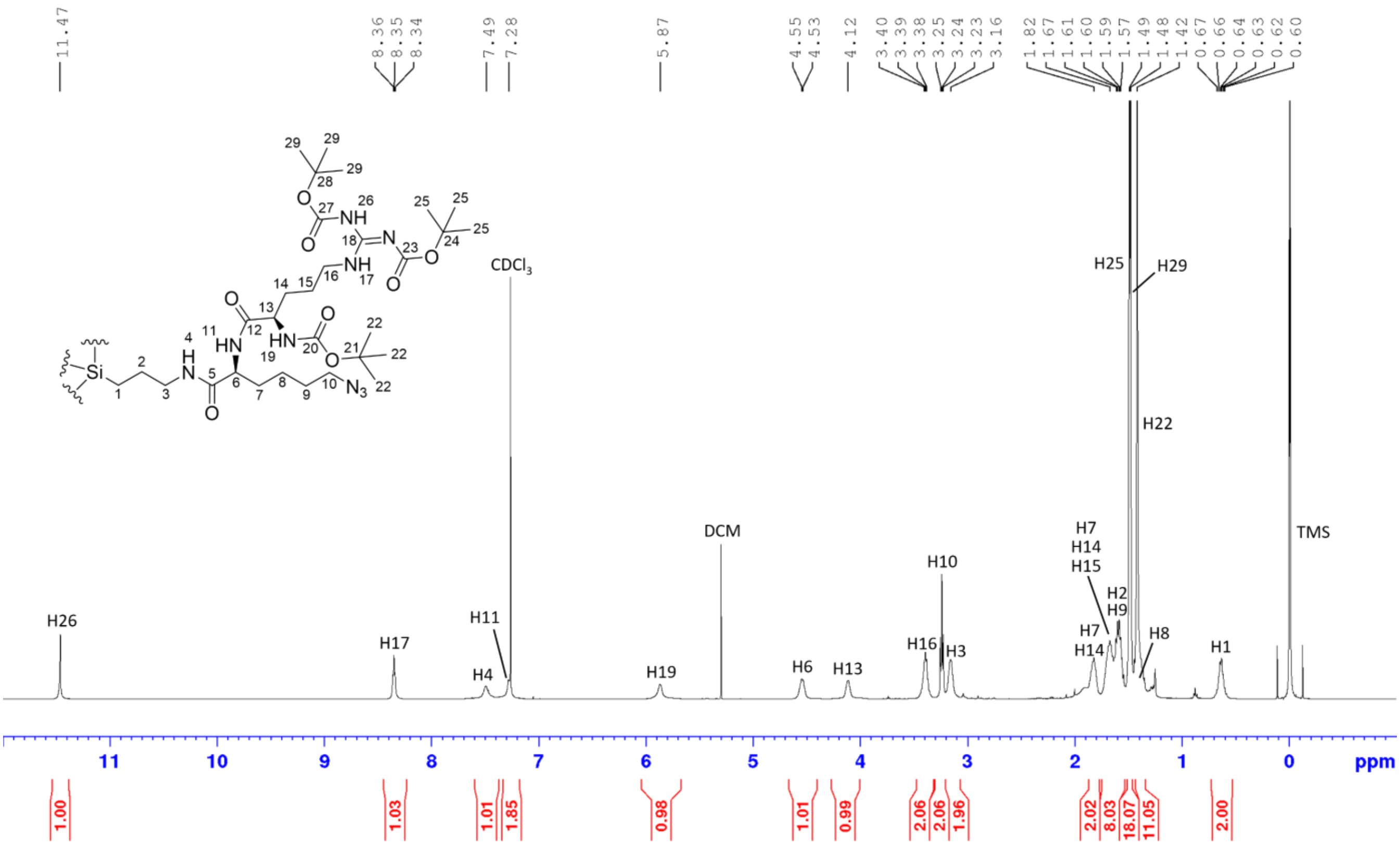

Figure S29. ^{1}H NMR spectrum of **15** ($CDCl_3$, 500 MHz, 25 °C).

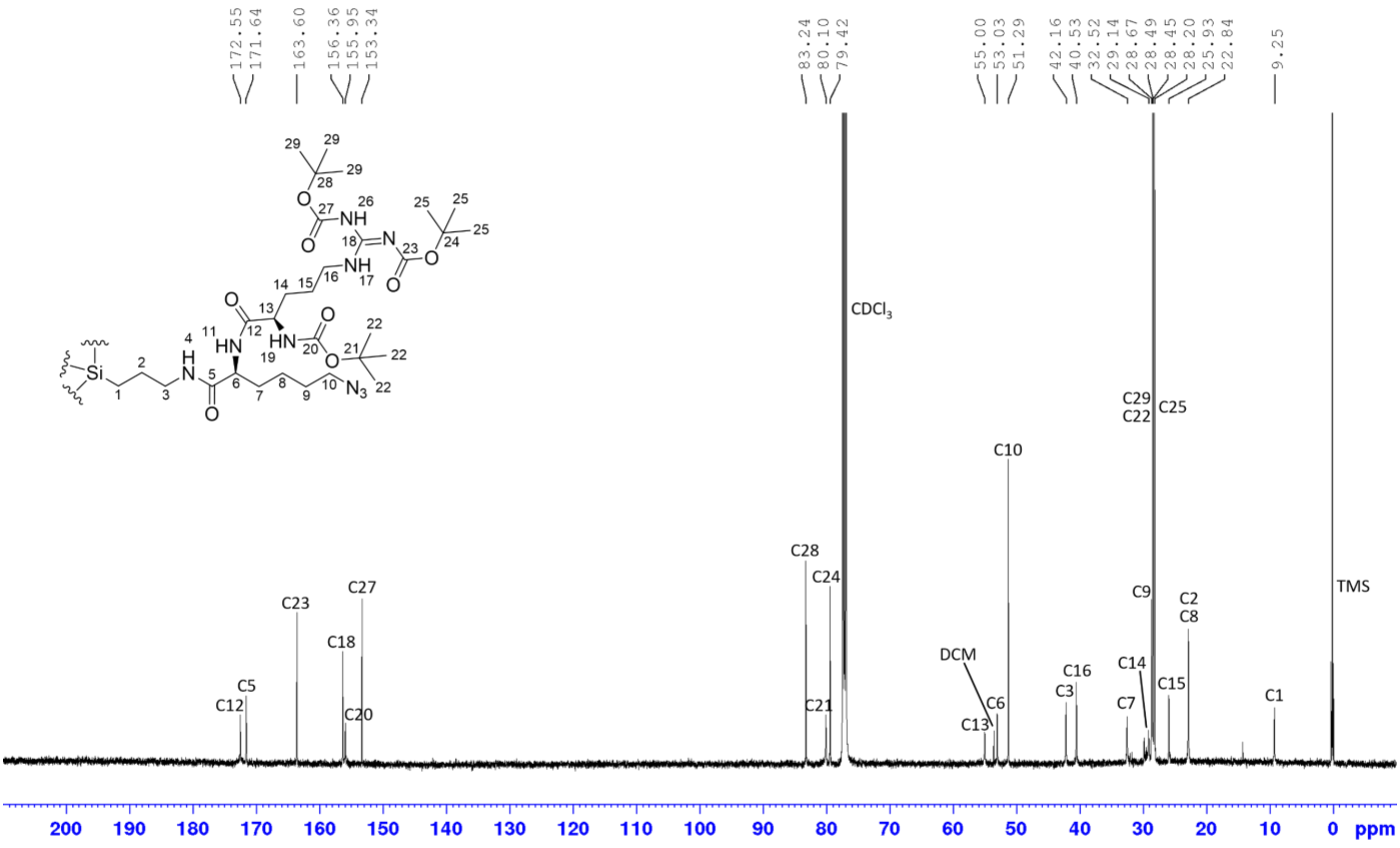

Figure S30. $^{13}C\{^{1}H\}$ NMR spectrum of **15** ($CDCl_3$, 126 MHz, 25 °C).

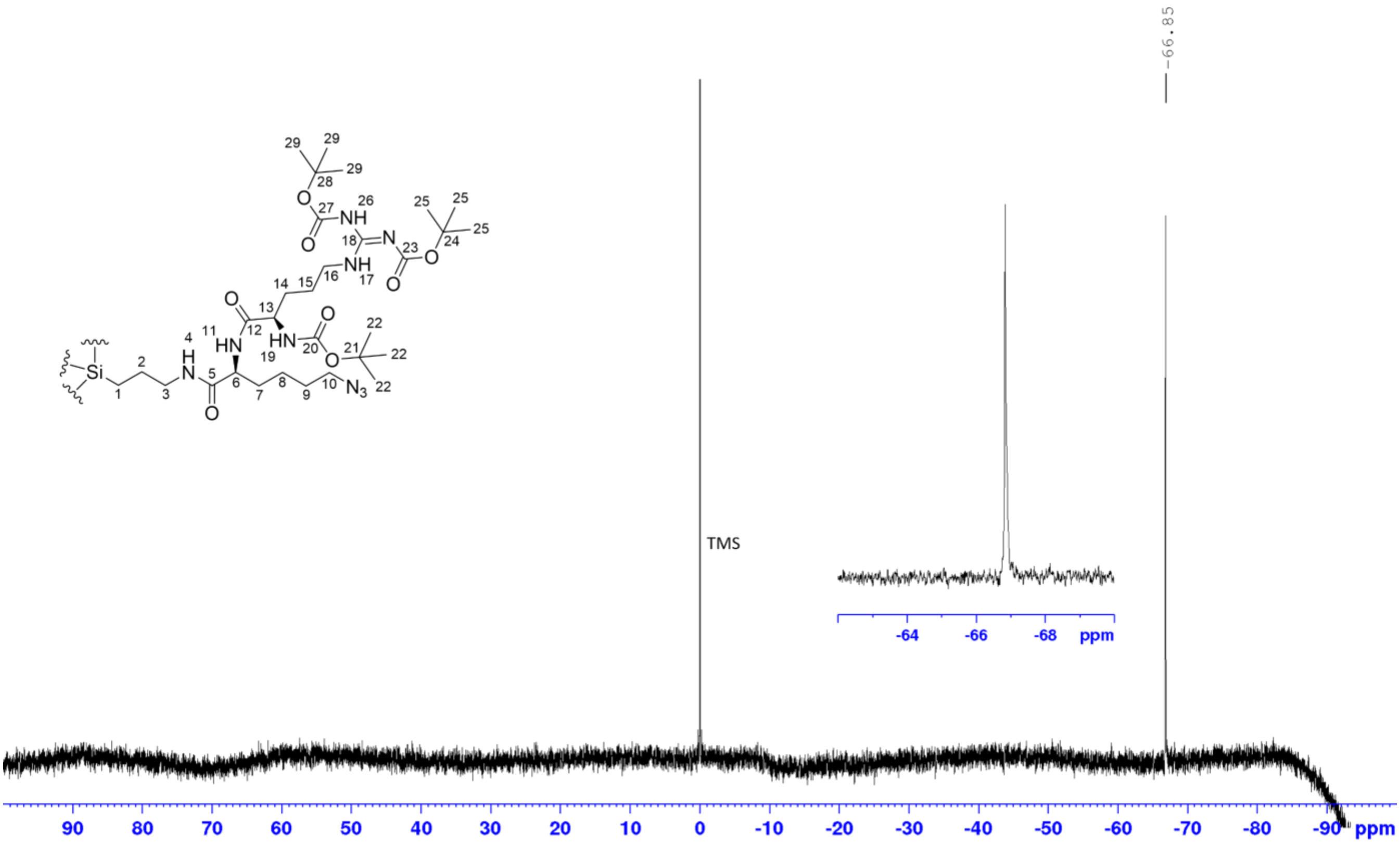

Figure S31. $^{29}Si\{^{1}H\}$ NMR spectrum of **15** ($CDCl_3$, 99 MHz, 25 °C).

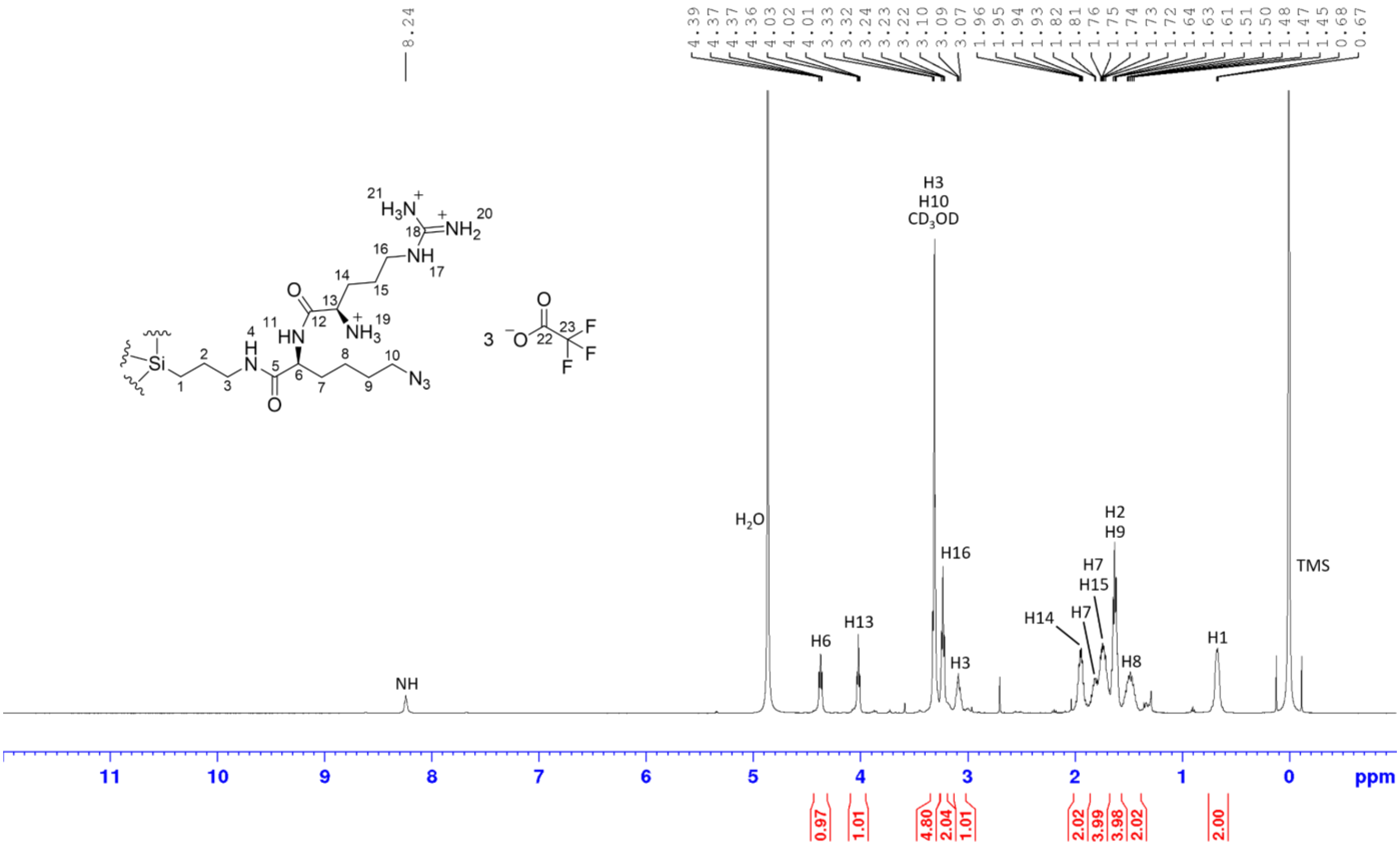

Figure S32. ^{1}H NMR spectrum of **3** (CD_3OD, 500 MHz, 25 °C).

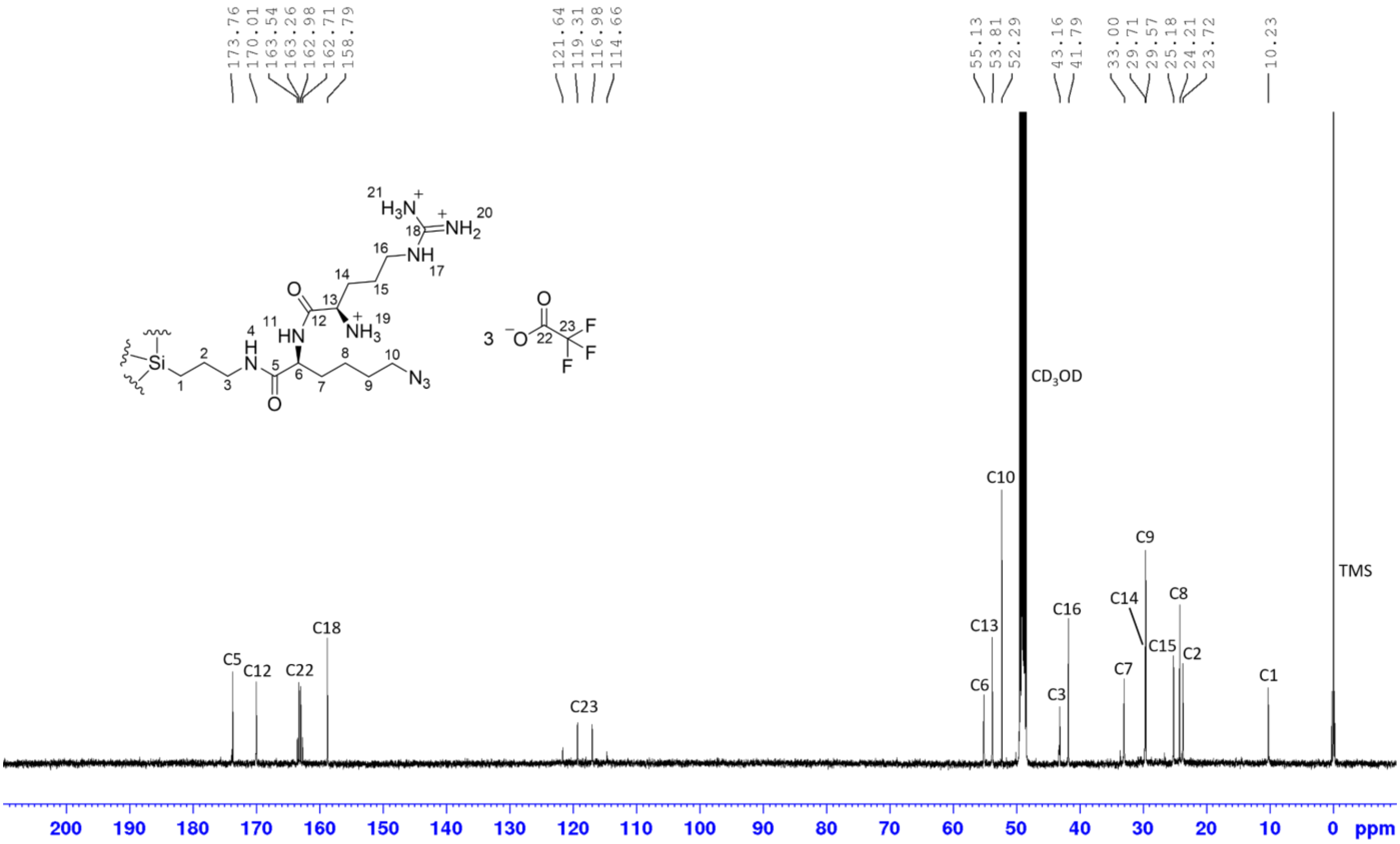

Figure S33. $^{13}C\{^1H\}$ NMR spectrum of **3** (CD_3OD, 126 MHz, 25 °C).

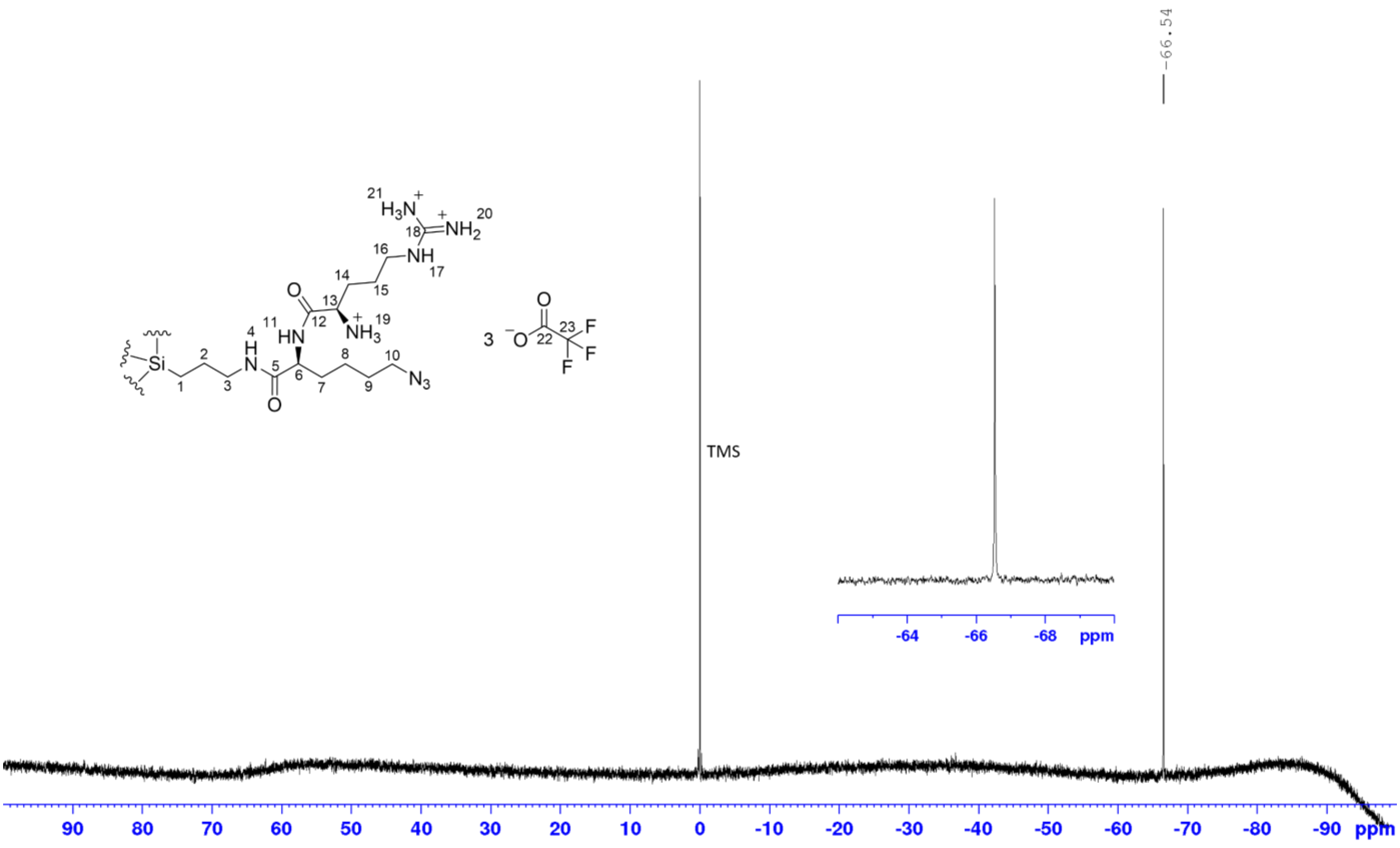

Figure S34. $^{29}Si\{^1H\}$ NMR spectrum of **3** (CD_3OD, 99 MHz, 25 °C).

3 Mass Spectra

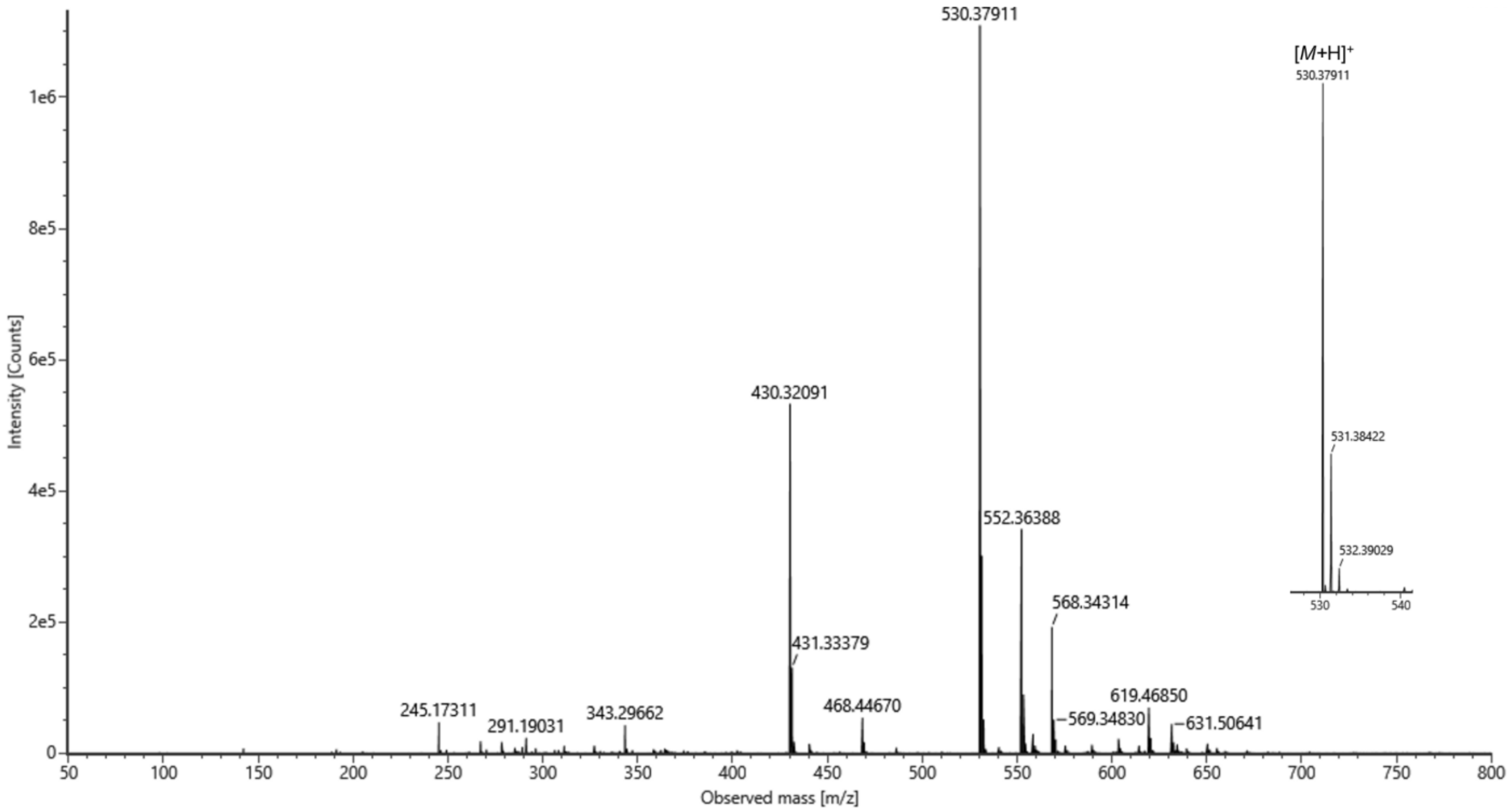

Figure S35. Mass spectrum of **6** (ESI+).

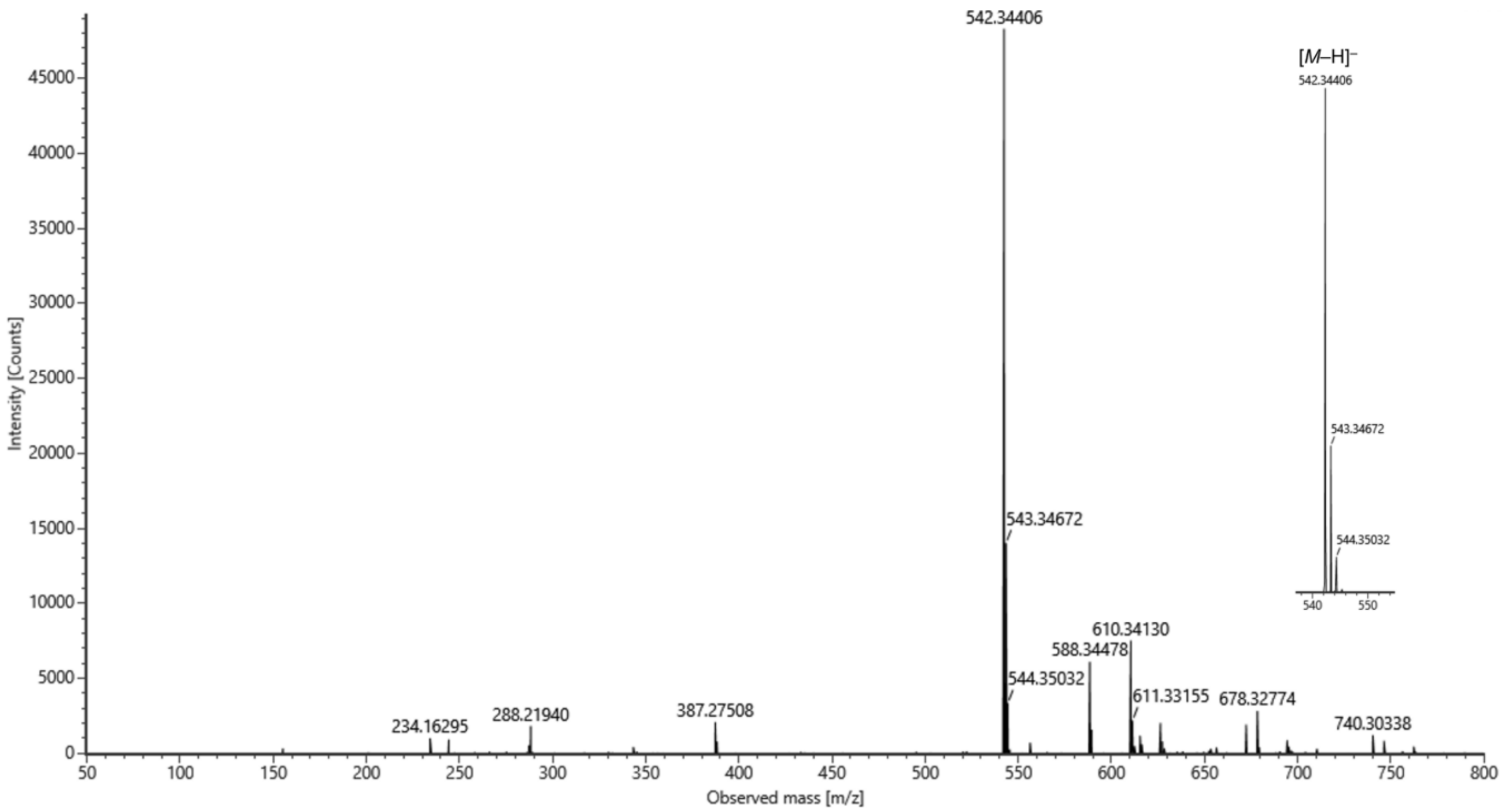

Figure S36. Mass spectrum of **4** (ESI–).

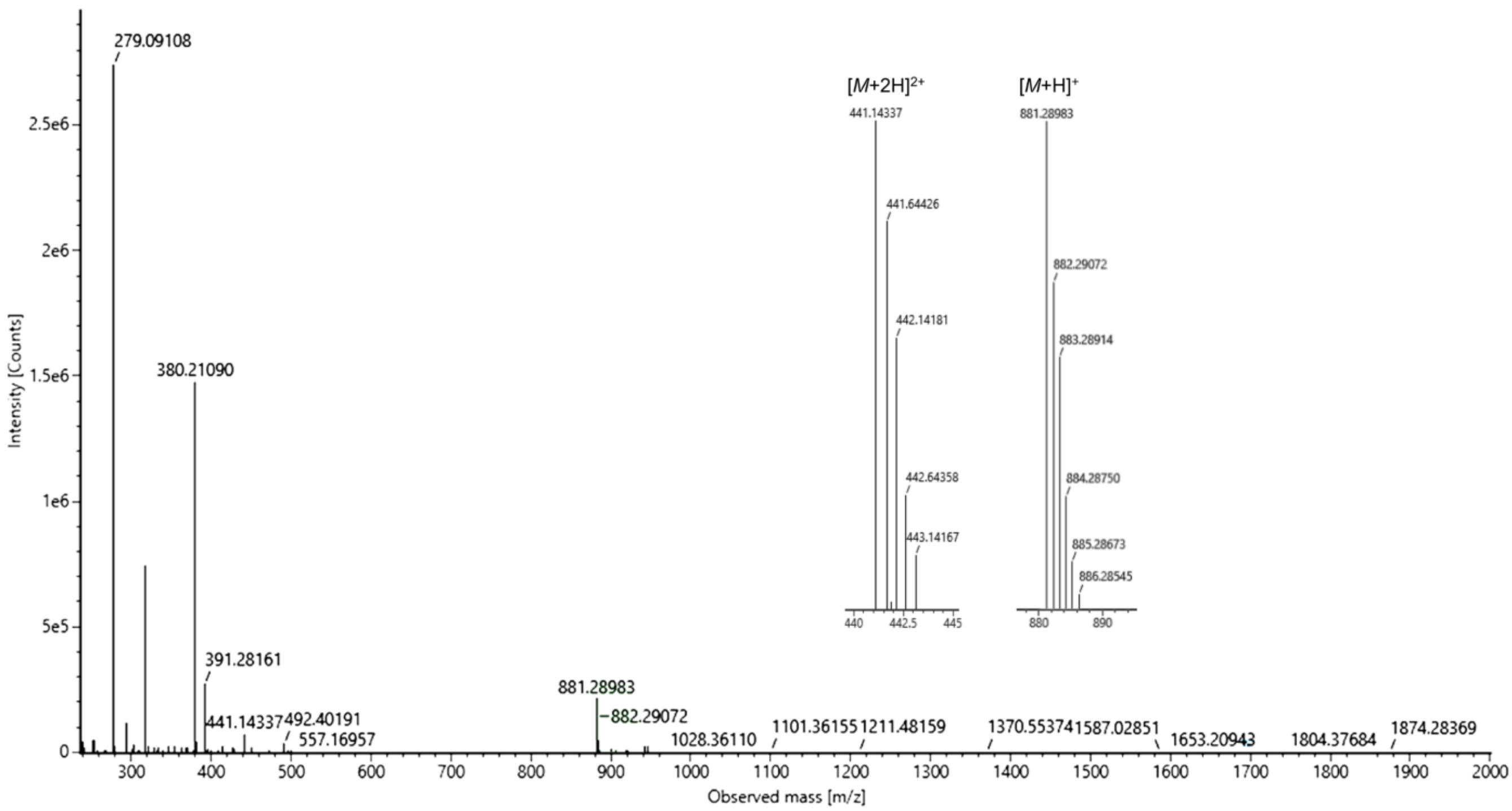

Figure S37. Mass spectrum of **7** (ESI+).

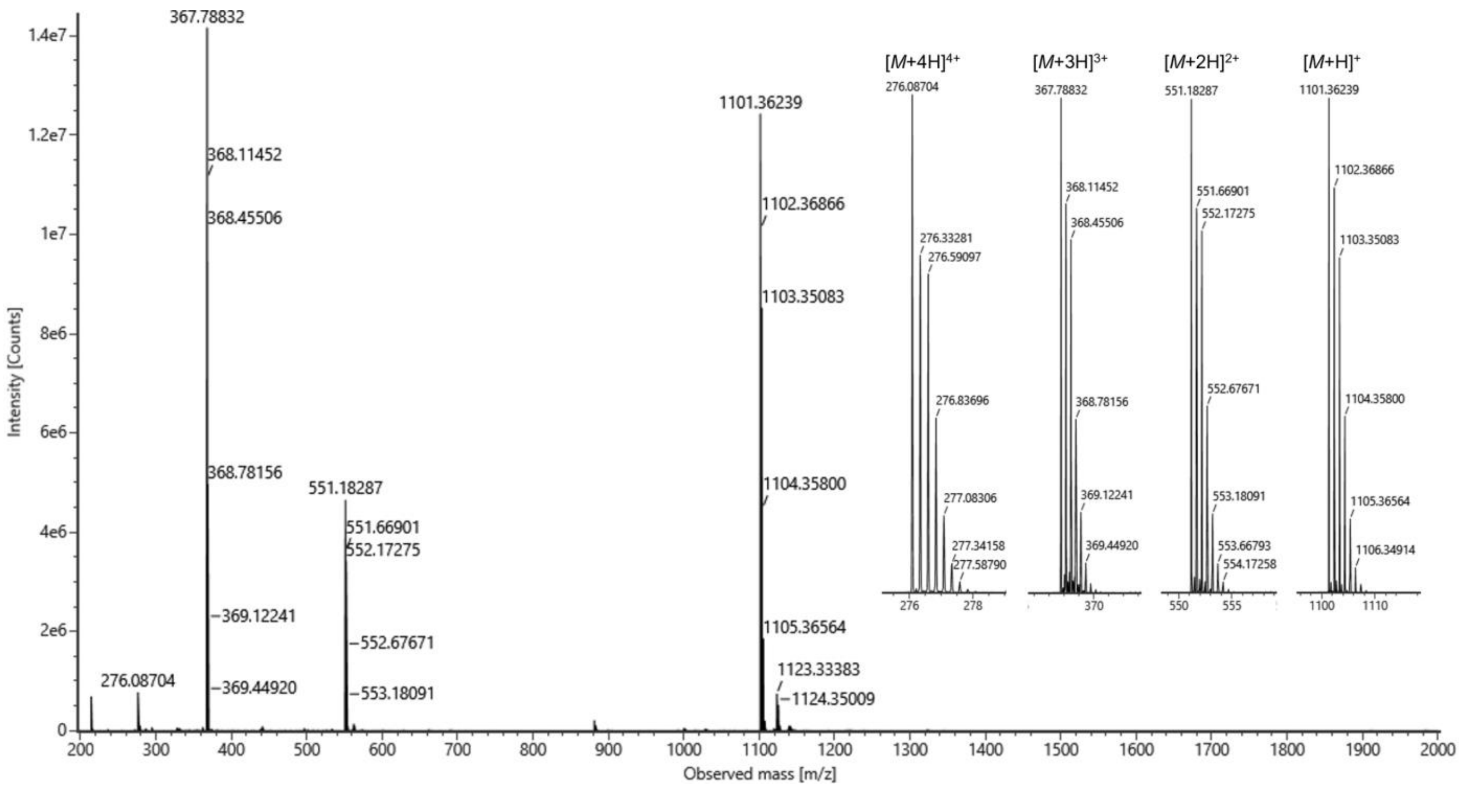

Figure S38. Mass spectrum of **16** (ESI+).

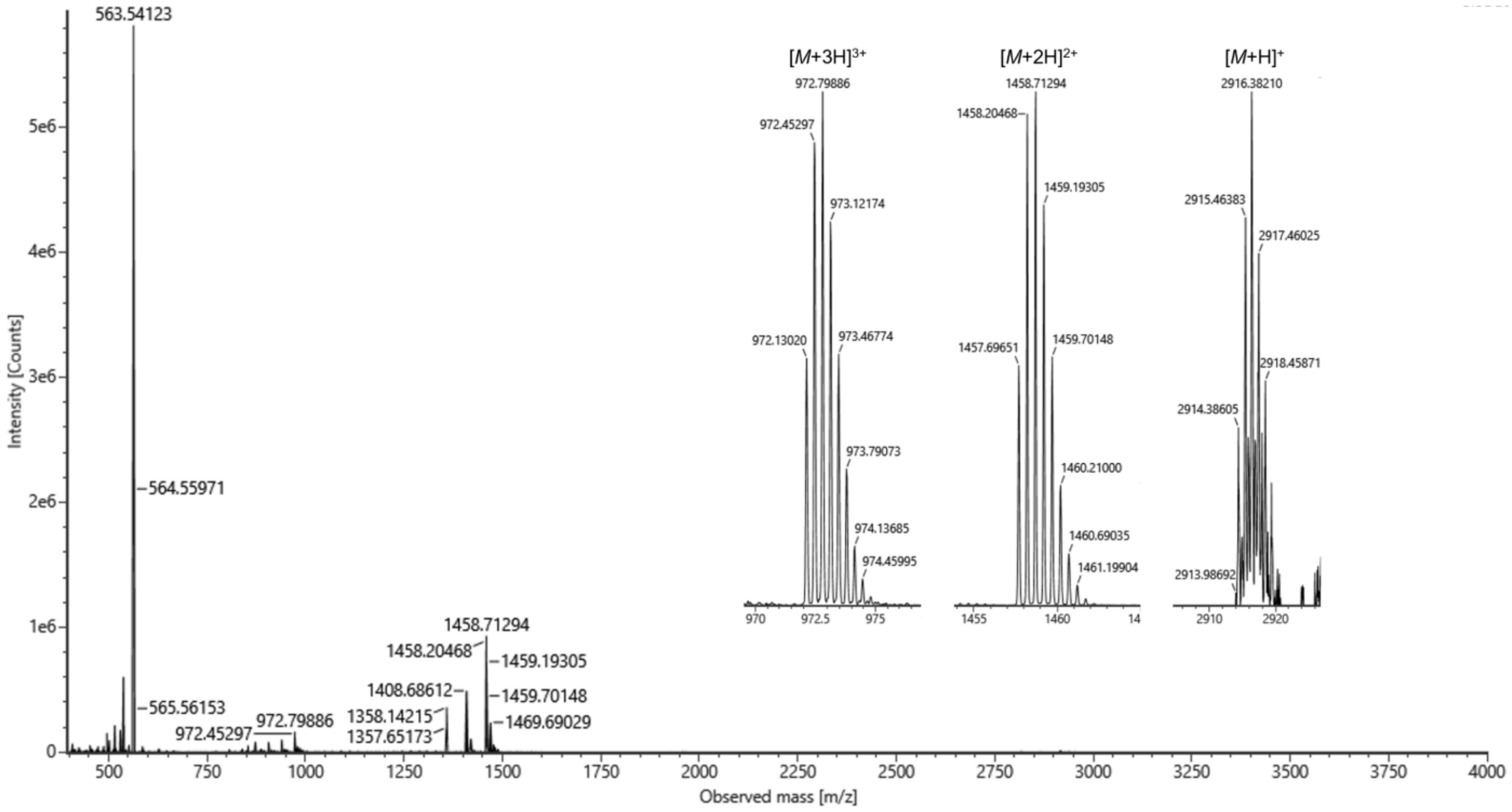

Figure S39. Mass spectrum of **8** (ESI+).

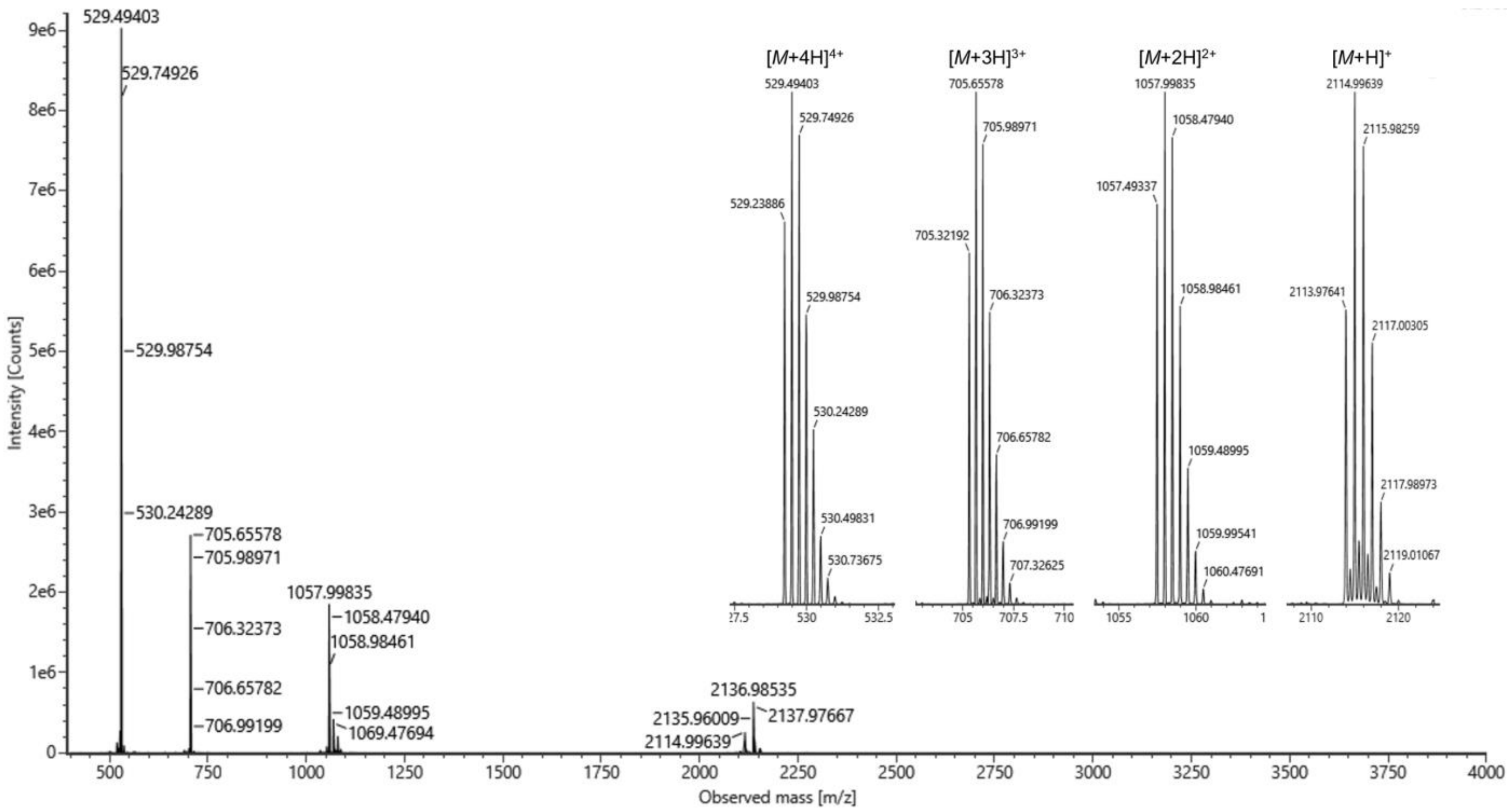

Figure S40. Mass spectrum of **10** (ESI+).

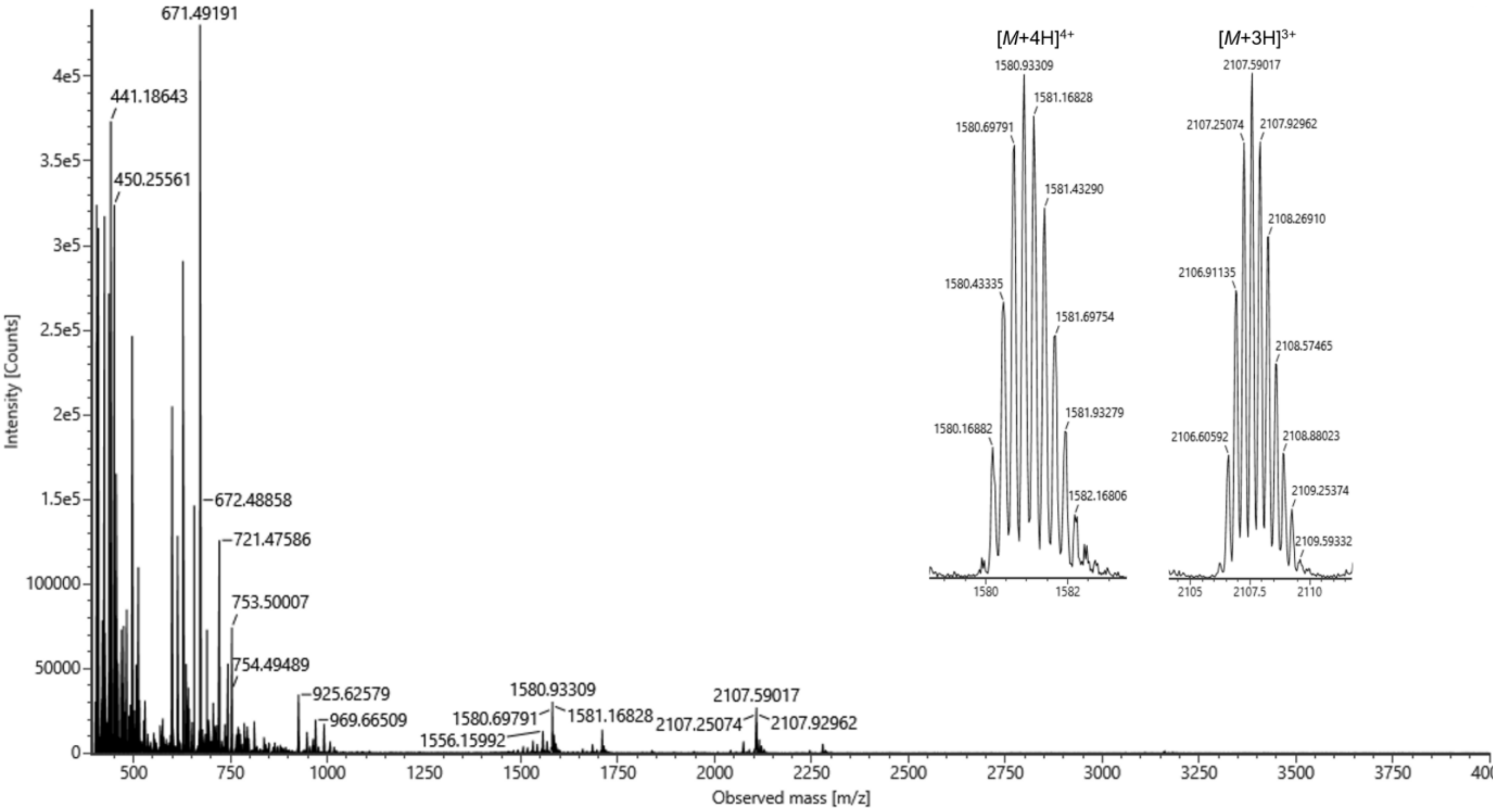

Figure S41. Mass spectrum of **11** (ESI+).

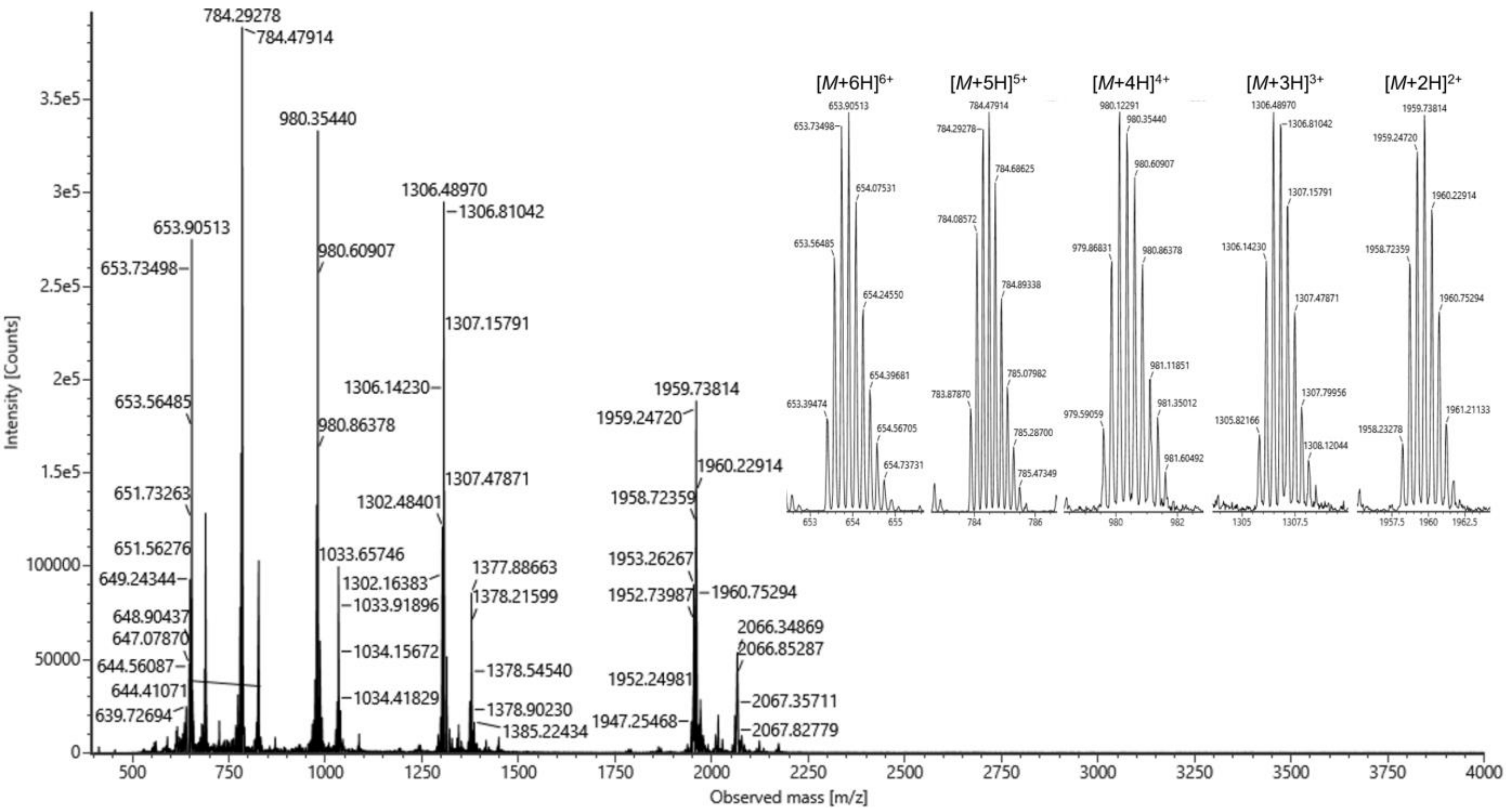

Figure S42. Mass spectrum of **1** (ESI+).

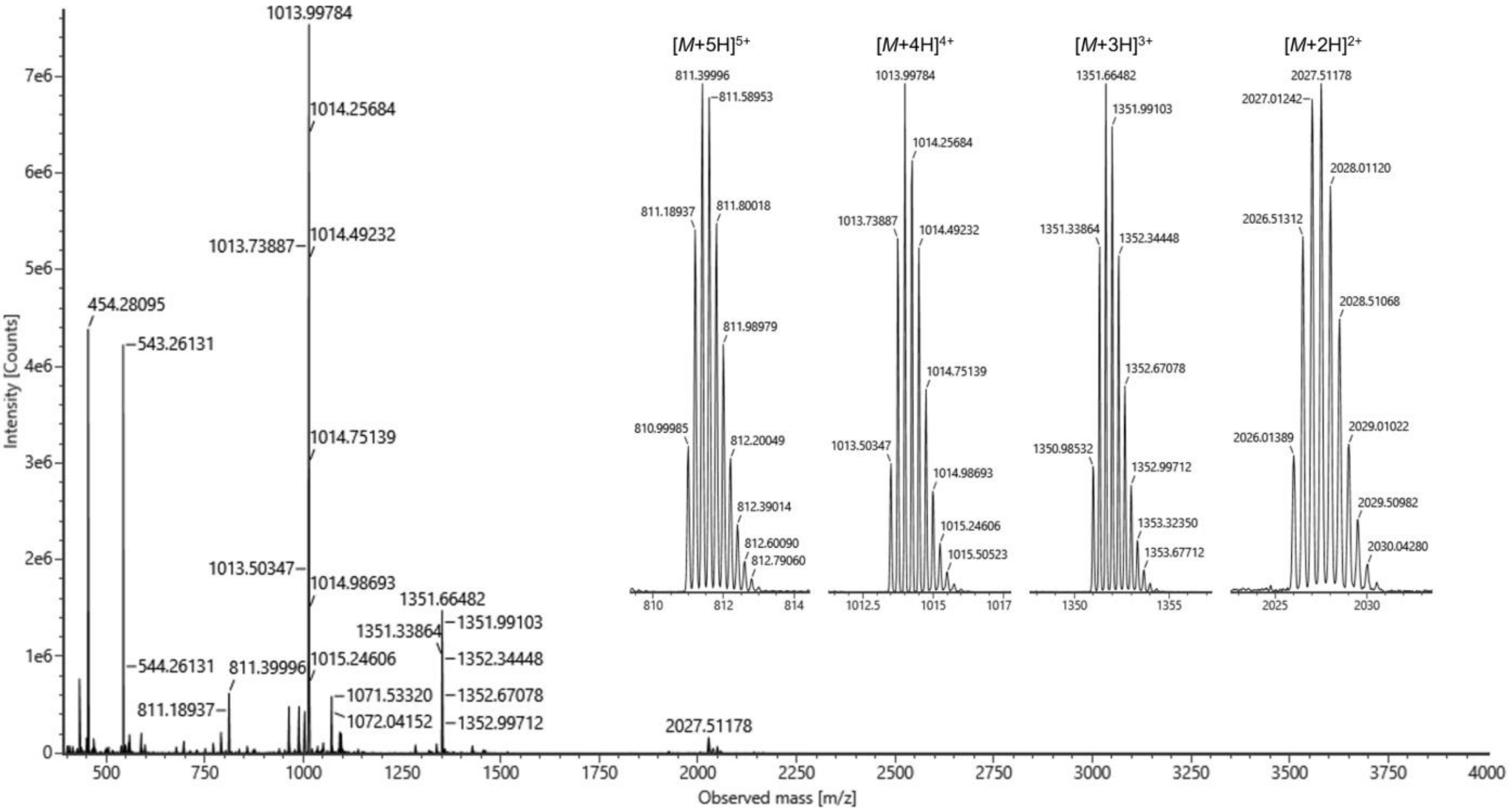

Figure S43. Mass spectrum of **13** (ESI+).

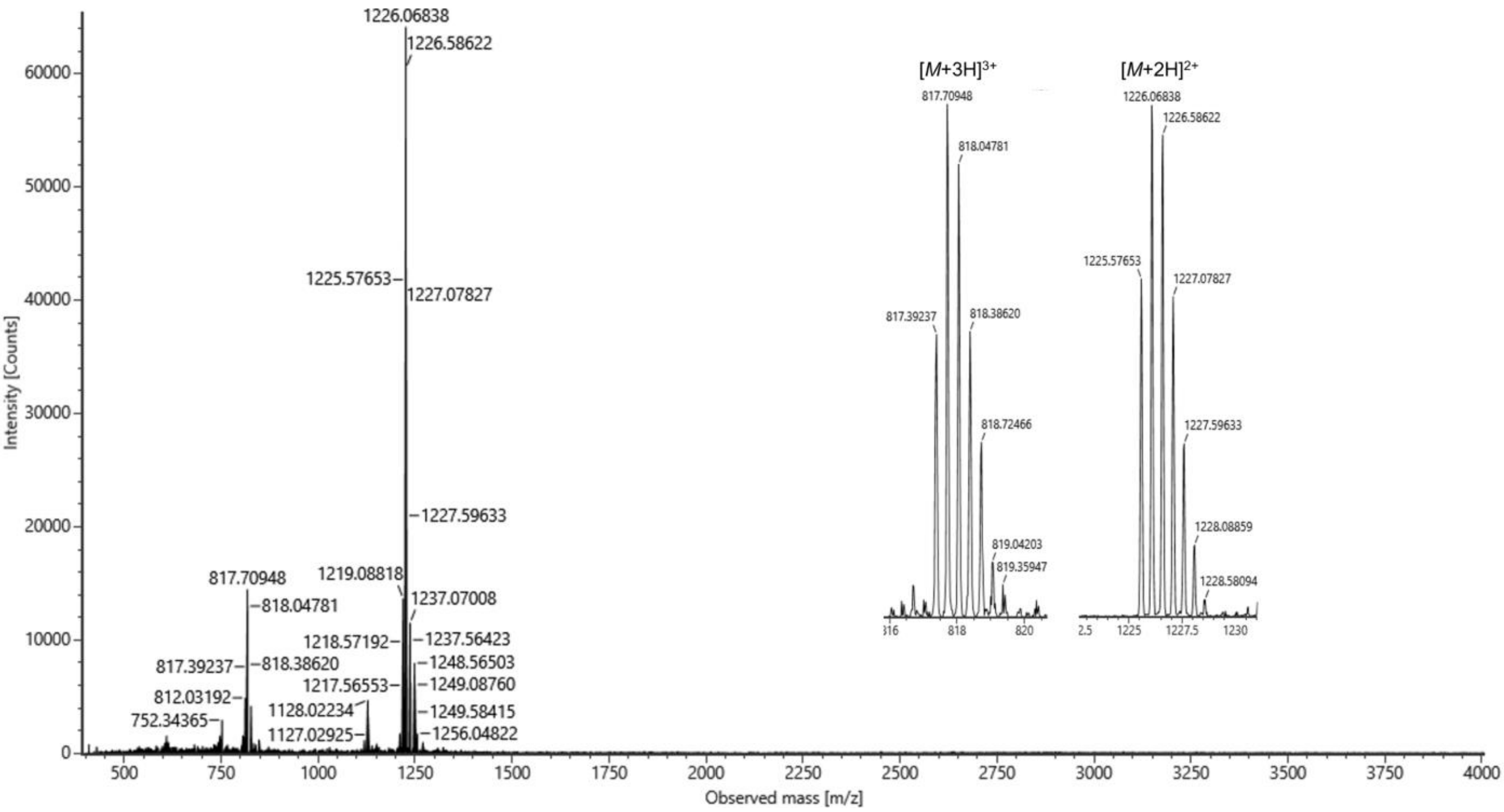

Figure S44. Mass spectrum of **2** (ESI+).

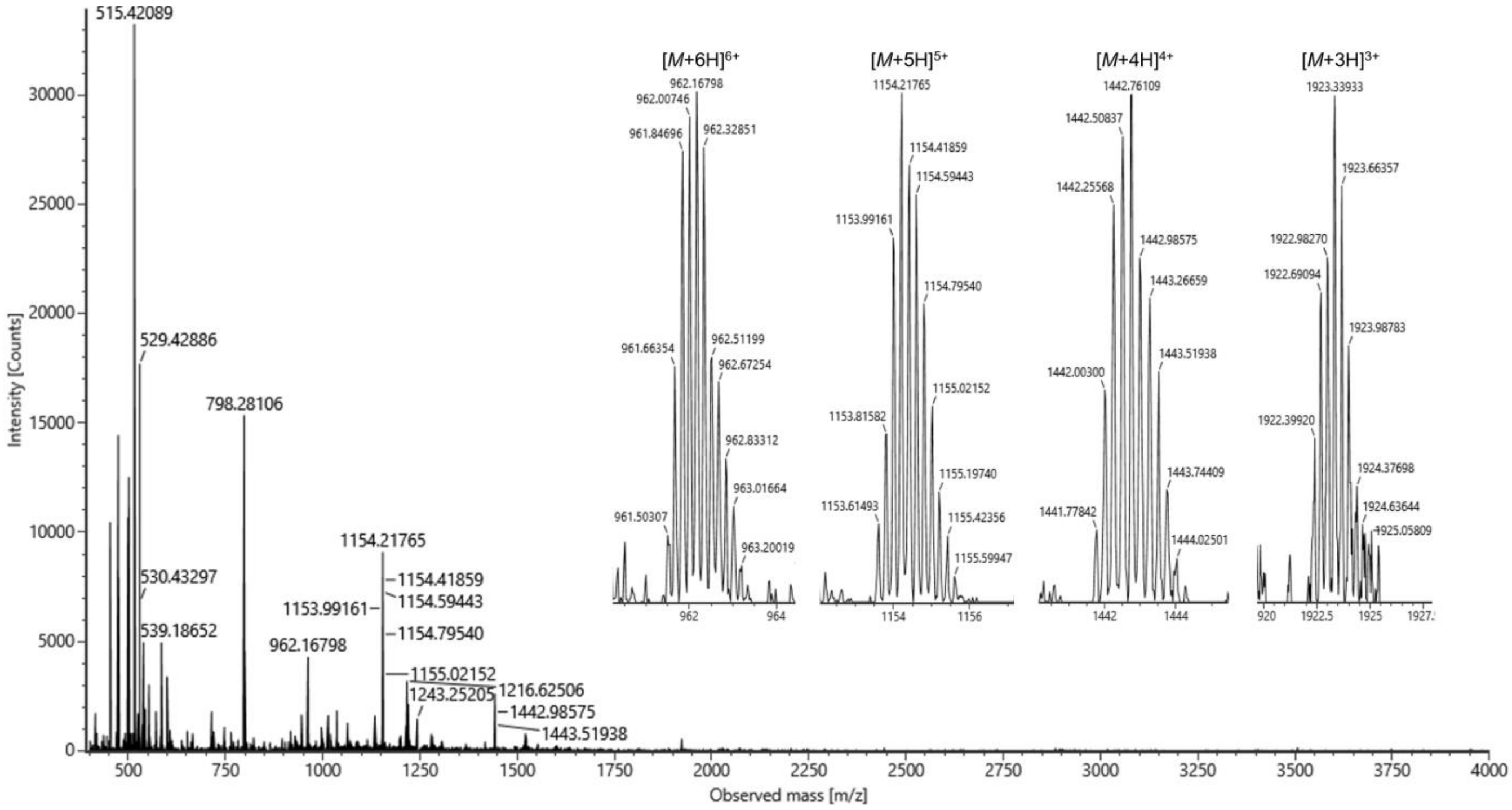

Figure S45. Mass spectrum of **15** (ESI+).

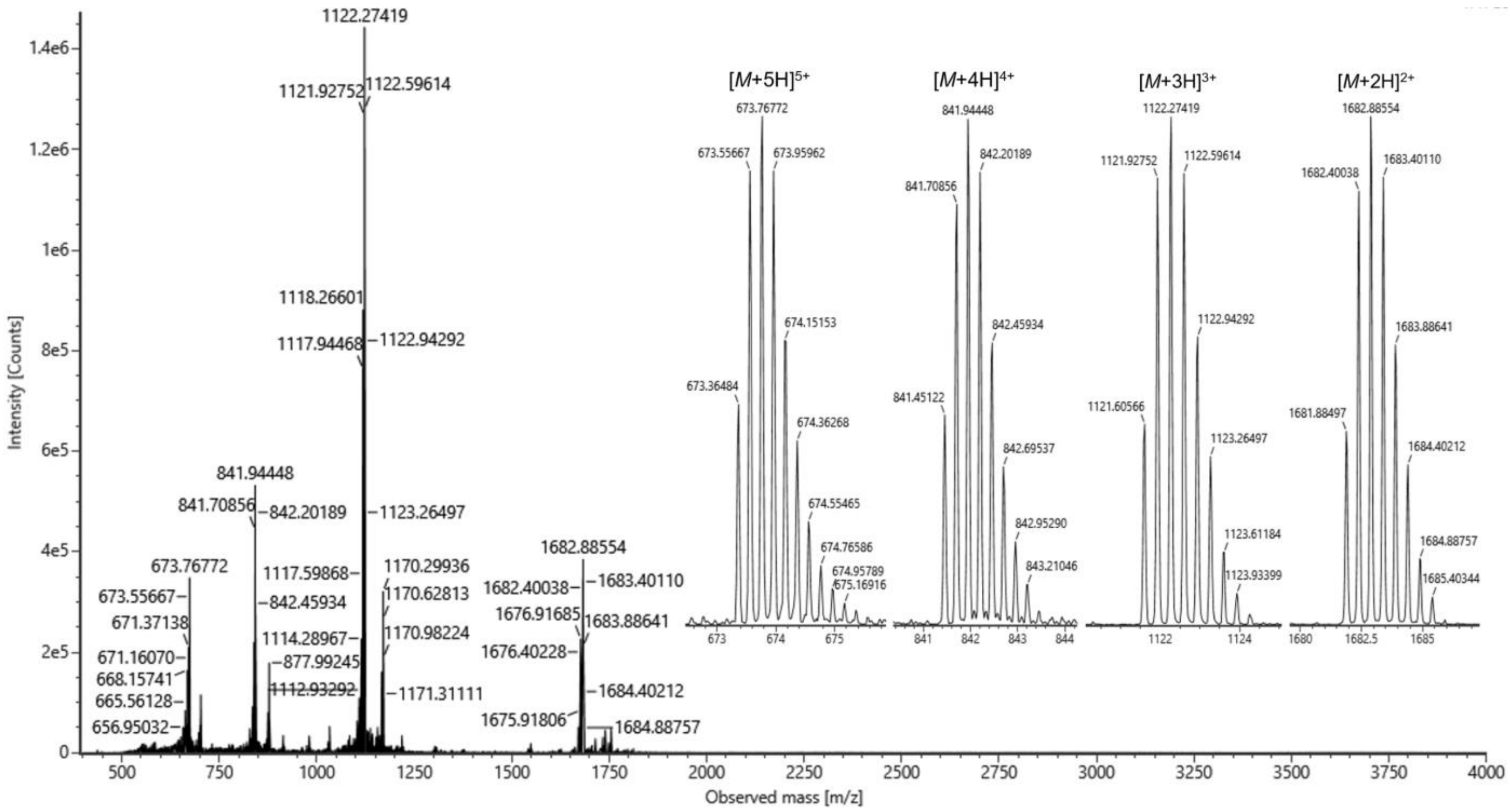

Figure S46. Mass spectrum of **3** (ESI+).
